# Curcumol Overcomes TRAIL Resistance of Non‐Small Cell Lung Cancer by Targeting NRH:Quinone Oxidoreductase 2 (NQO2)

**DOI:** 10.1002/advs.202002306

**Published:** 2020-10-15

**Authors:** Jing Zhang, Ye Zhou, Nan Li, Wan‐Ting Liu, Jun‐Ze Liang, Yue Sun, Wei‐Xia Zhang, Run‐Dong Fang, Sheng‐Ling Huang, Zheng‐Hua Sun, Yang Wang, Qing‐Yu He

**Affiliations:** ^1^ MOE Key Laboratory of Tumor Molecular Biology and Key Laboratory of Functional Protein Research of Guangdong Higher Education Institutes Institute of Life and Health Engineering College of Life Science and Technology Jinan University Guangzhou 510632 China; ^2^ The First Affiliated Hospital Jinan University Guangzhou 510632 China

**Keywords:** cellular thermal shift assay profiling, curcumol, non‐small cell lung cancer, quinone oxidoreductase 2, tumor‐necrosis‐factor‐related apoptosis‐inducing ligand resistance

## Abstract

Resistance to tumor‐necrosis‐factor‐related apoptosis‐inducing ligand (TRAIL) of cancer cell remains a key obstacle for clinical cancer therapies. To overcome TRAIL resistance, this study identifies curcumol as a novel safe sensitizer from a food‐source compound library, which exhibits synergistic lethal effects in combination with TRAIL on non‐small cell lung cancer (NSCLC). SILAC‐based cellular thermal shift profiling identifies NRH:quinone oxidoreductase 2 (NQO2) as the key target of curcumol. Mechanistically, curcumol directly targets NQO2 to cause reactive oxygen species (ROS) generation, which triggers endoplasmic reticulum (ER) stress‐C/EBP homologous protein (CHOP) death receptor (DR5) signaling, sensitizing NSCLC cell to TRAIL‐induced apoptosis. Molecular docking analysis and surface plasmon resonance assay demonstrate that Phe178 in NQO2 is a critical site for curcumol binding. Mutation of Phe178 completely abolishes the function of NQO2 and augments the TRAIL sensitization. This study characterizes the functional role of NQO2 in TRAIL resistance and the sensitizing function of curcumol by directly targeting NQO2, highlighting the potential of using curcumol as an NQO2 inhibitor for clinical treatment of TRAIL‐resistant cancers.

## Graphical abstract

This study discovered that curcumol sensitizes lung cancer cell to tumor‐necrosis‐factor‐related apoptosis‐inducing ligand (TRAIL)‐mediated apoptosis. Curcumol exhibits synergistic lethal effects with TRAIL on cancer cell by directly targeting quinone oxidoreductase 2 (NQO2) to induce reactive oxygen species, triggering ER stress‐C/EBP homologous protein (CHOP)‐death receptor 5 (DR5) signaling to combat TRAIL‐resistant cancer.

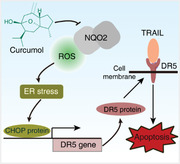

## 1 Introduction

Specifically targeting cancer cells via the extrinsic cell death machinery involving death receptors of the TNF superfamily has become an effective approach in anticancer therapy.^[^ ^1^ ^]^ Tumor‐necrosis‐factor‐related apoptosis‐inducing ligand (TRAIL) is able to induce canonical apoptotic cell death in cancer cells without causing toxicity, and therefore is considered as an attractive agent for cancer therapy.^[^ ^2^ ^]^ However, majority of cancer cell lines and primary tumors such as non‐small cell lung cancer (NSCLC) are TRAIL resistant,^[^ ^3^ ^]^ resulting in a universal treatment failure of late‐stage cancer patients by TRAIL.^[^ ^4^, ^5^ ^]^ Increasing evidences indicated that TRAIL death receptors (DRs) are functionally defective in different types of cancer cells.^[^ ^6^ ^]^ Therefore, re‐establishing TRAIL receptor in cancer cell is an effective strategy for developing biotherapeutic drugs to overcome TRAIL resistance.

Food‐source compounds are a major source of chemotherapeutic drugs for clinical cancer therapy, due to not only their potential anticancer effects but also their stability and safety for the clinical application.^[^ ^7^ ^]^ Curcumol is a guaiane‐type sesquiterpenoid hemiketal isolated from herbal medicine plant Rhizoma Curcumae, which has been reported to exhibit anti‐inflammatory^[^ ^8^ ^]^ and anticancer effects in multiple tumor cell lines, without causing side effects.^[^ ^9^ ^]^ In hepatic stellate cells, curcumol was reported to inducereceptor‐interacting protein kinase 1/receptor‐interacting protein kinase 3 (RIPK1/RIPK3) complex‐dependent necroptosis through c‐Jun N‐terminal kinase (JNK)‐reactive oxygen species (ROS) pathway.^[^ ^10^ ^]^ Despite these observations, the function and molecular mechanism of curcumol in TRAIL‐induced apoptosis are poorly understood.

The therapeutic effect of small‐molecule drugs is usually achieved through direct binding to protein targets, however, identification of target engagement by drugs in cells is often challenging.^[^ ^11^, ^12^ ^]^ Ligand‐induced alterations in protein thermal stability are often applied in investigating the biophysical interaction between target proteins and small molecules according to the melting transitions.^[^ ^13^ ^]^ This method is achieved in the context of cell lysates, which preserves the original environment and posttranslational modifications for the endogenous proteins. By coupling with high‐throughput proteomics, the drug target engagement can be measured and quantified. Stable isotope labelling (SILAC) can provide actual quantitation in a proteome‐wide scale in cell.^[^ ^14^, ^15^ ^]^ We here developed a precise and universally applicable target identification approach by combining SILAC proteomic technology with cellular thermal shift assay (CETSA) to globally analyze direct target proteins for small molecules.

To obtain clinically potential drugs that elicit synergistic lethal effects on TRAIL‐treated NSCLC cells, we performed a screening with a food‐source compound library. Curcumol emerged as the top candidate that exhibited synergistic lethal effects on TRAIL‐treated NSCLC in vivo and in vitro, with nontoxicity in monotherapy. SILAC‐based CETSA profiling was implemented to globally profile curcumol‐protein bindings, and NRH:Quinone Oxidoreductase 2 (NQO2) was identified as a key target of curcumol. Further mechanistic investigation revealed that curcumol blocked the active site Phe178 of NQO2, suppressed NQO2 activity to cause ROS generation, initiating endoplasmic reticulum (ER) stress‐C/EBP homologous protein (CHOP)‐death receptor (DR5) signaling for sensitizing NSCLC to TRAIL‐induced apoptosis, highlighting a critical role of NQO2 in TRAIL resistance and a synergistic lethal effect of curcumol/TRAIL in cancer therapy.

## 2 Results

### 2.1 Curcumol Sensitizes NSCLC Cells to TRAIL‐Induced Apoptosis In Vitro and In Vivo

**Figure 1:**
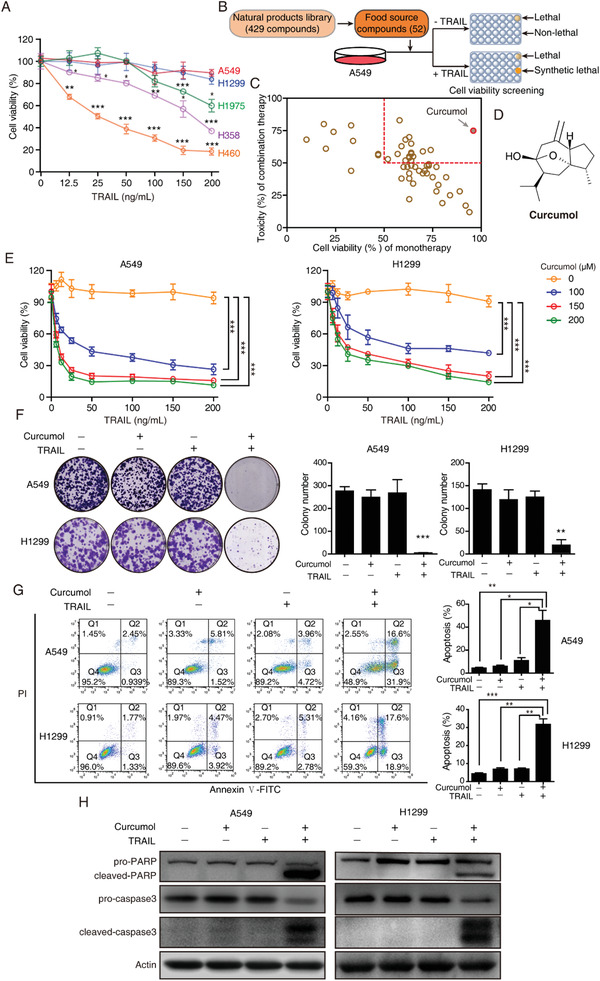
Food‐source drug library screening identifies curcumol as a potential sensitizer for TRAIL‐induced apoptosis. A) NSCLC cells were treated with TRAIL for 24 h by elevating concentrations (up to 200 ng mL^−1^), and the cell viability was quantified using WST‐1 assay. Bars, SEM; *n* = 3; *p < 0.05, **p < 0.01 compared with their control group (0 ng mL^−1^), respectively. B) Flow chart for food‐source drug library screening to identify compounds constituting a synergistic lethal effect with TRAIL. A total of 52 food‐source compounds were selected from a library of 429 natural product compounds according to literature study. A549 cells were treated with each selected compound (10 × 10^−5^ m) with or without TRAIL for 24 h, and cell viability was assessed. C) Curcumol emerged as the top hit to elicit synergistic lethality with TRAIL in A549 cells. D) Chemical structure of curcumol. E) A549 and H1299 cells incubated with increasing concentration of curcumol were combinationally treated with the indicated concentration of TRAIL for 24 h, then cell viability was quantified using WST‐1 assay. F–H) A549 and H1299 cells treated with indicated curcumol (15 × 10^−5^ m) and/or TRAIL (25 ng mL^−1^ for A549, 50 ng mL^−1^ for H1299) and their abilities to form colonies were analyzed F). The apoptotic cells with indicated treatment for 24 h were detected by Annexin V‐FITC/PI double staining assay G). The protein level of apoptosis‐associated proteins including pro‐PARP, cleaved‐PARP, pro‐caspase3, and cleaved‐caspase3 were detected by Western blotting H). Bars, SEM; *n* = 3; *p < 0.05, **p < 0.01, or ***p < 0.001.

Resistance to TRAIL treatment is frequently observed in clinical trials of NSCLC.^[^ ^16^ ^]^ We compared the cell viability of different NSCLC cell lines including A549, H1299, H1975, H358, and H460 in response to TRAIL challenge. Consistent with a previous study,^[^ ^17^ ^]^ A549 and H1299 showed strong resistance to TRAIL, while H460 was sensitive to TRAIL with ≈25 ng mL^−1^ IC50 (**Figure** **1**A). To identify synergistic lethal compounds associated with TRAIL treatment with high safety, we screened 52 food‐source compounds generated from a drug library containing 429 natural products (Figure 1B). To obtain the most potent and selective compounds, positive hits were defined as the compounds exhibiting high cytotoxic effects in combination treatment with TRAIL, but nontoxicity in single treatment (Figure 1C, Table S1, Supporting Information). As a result, curcumol emerged as a top hit to elicit remarkably synergistic lethal effects in the combination treatment with TRAIL (Figure 1D).

We then confirmed that stimulation with curcumol sensitized A549 and H1299 cells to TRAIL in a dose‐dependent manner (Figure 1E). In long‐term colony formation assay and short‐term Annexin V‐FITC/PI assay, individual treatment with curcumol (up to 20 × 10^−5^ m) or TRAIL (up to 200 ng mL^−1^) alone did not influence the cell growth and cell viability (Figure S1A–C, Supporting Information), but synergistic lethal effects were found in the combination of curcumol with TRAIL (Figure 1F,G). In addition, we found only a slight inhibition of cell viability by improving curcumol concentration until 40 × 10^−5^ m (Figure S1D, Supporting Information). The sensitivity of resistant NSCLC cells to TRAIL was correlated with an increase of extrinsic apoptosis, as evidenced by the presence of Annexin V positive, cleaved‐PARP, and cleaved‐caspase3 expression (Figure 1H), and the alteration in cell morphology (Figure S2A, Supporting Information). To further confirm curcumol/TRAIL‐induced apoptotic cell death, A549 and H1299 cells were, respectively, co‐incubated with curcumol/TRAIL in the presence of Z‐VAD‐FMK, a pan‐caspase inhibitor. The results showed that Z‐VAD‐FMK could effectively attenuate the curcumol/TRAIL‐induced cell death (Figure S2B, Supporting Information) and suppress the expression of cleaved‐caspase3 and cleaved‐PARP (Figure S2C, Supporting Information). Moreover, we found that curcumol alone or in combination with TRAIL did not influence p53 expression (Figure S2D,E, Supporting Information), suggesting that p53 may not be involved in the curcumol mediated synergistic lethal effects.

**Figure 2:**
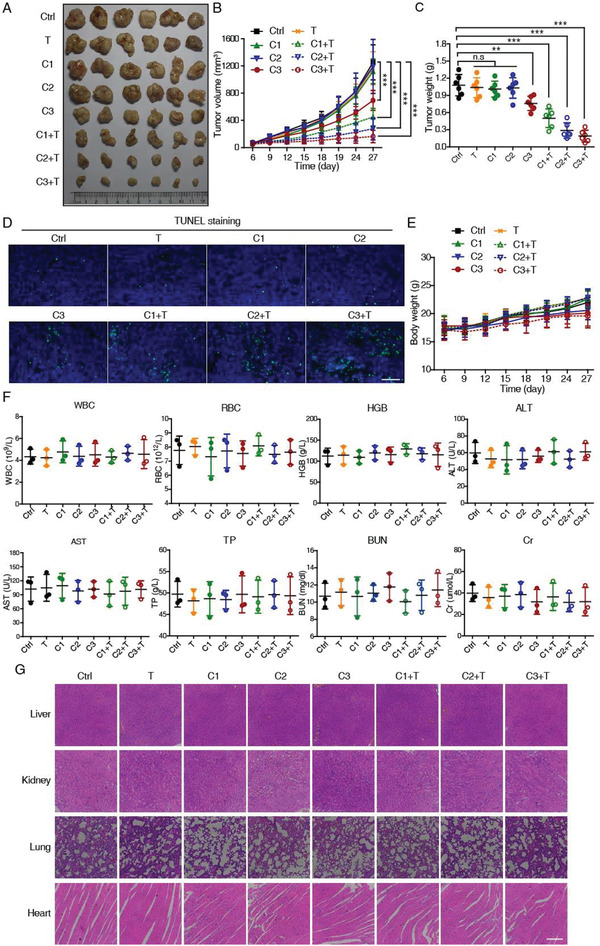
Synergistic anticancer effects of curcumol and TRAIL co‐treatment in subcutaneous tumor model. A) Representative photographs of the excised A549 tumors from control (Ctrl) and treatment groups. T, TRAIL (100 µg per mice); C1, curcumol (2 mg kg^−1^); C2, curcumol (10 mg kg^−1^); C3, curcumol (50 mg kg^−1^); C1+T, curcumol (2mg kg^−1^) + TRAIL (100 µg per mice); C2+T, curcumol (10 mg kg^−1^) + TRAIL (100 µg per mice); C3+T, curcumol (50 mg kg^−1^) + TRAIL (100 µg per mice). B) Tumor growth curves showing the synergistic anticancer effect of curcumol and TRAIL on tumor xenografts (*n* = 6). The tumor weight of the eight groups was compared C) and the apoptotic cells in tissues were determined by TUNEL assay D). Blue, DAPI; green, TUNEL; scale bar, 50 µm. E) Body weight of the nude mice in the treatment and control groups as indicated was monitored. F) No significant differences were detected in the blood chemistry among the groups; WBC, white blood cells; RBC, red blood cells; HGB, hemoglobin; ALT, alanine aminotransferase; AST, aspartate aminotransferase; TP, total protein; BUN, blood urea nitrogen; Cr, creatinine. G) Representative images of H&E staining of the livers, kidneys, lungs, and hearts; scale bar, 200 µm. Bars, SD; **p < 0.01; ***p < 0.001; n.s, not significant.

To evaluate the therapeutic efficacy of curcumol/TRAIL combination in vivo, we established murine xenografts using A549 cells, and tested the effect of curcumol at low (2 mg kg^−1^), medium (10 mg kg^−1^), and high dosage (50 mg kg^−1^), alone and in combination with TRAIL (100 µg per mice). Consistent with the in vitro findings, administration of curcumol alone had negligible effects on the burdened tumors until high dosage (50 mg kg^−1^), whereas co‐treatment with curcumol and TRAIL induced drastic anticancer effects in dose‐dependent manners (**Figure** **2**A), as indicated by tumor grow curve (Figure 2B) and tumor weights (Figure 2C). According to the TUNEL staining results (Figure 2D), combination of TRAIL and curcumol induced dramatic apoptosis of tumor cells over other groups. Importantly, the administration of curcumol/TRAIL together did not cause side effects to the mice, as evidenced by the lack of significant changes in body weight (Figure 2E), and blood biochemical and hematological parameters including WBC (white blood cells), RBC (red blood cells), HGB (hemoglobin), ALT (alanine aminotransferase), AST (aspartate aminotransferase), TP (total protein), BUN (blood urea nitrogen), and Cr (creatinine) (Figure 2F). Histological examination of vital organs, including the liver, kidney, lung, and heart, did not reveal any overt changes in morphology (Figure 2G). These data suggested that curcumol/TRAIL treatment exhibits a strong anticancer effect on NSCLC, with no toxic side effects on animals.

### 2.2 SILAC‐Based CETSA Profiling Identifies NQO2 as a Key Target of Curcumol

**Figure 3:**
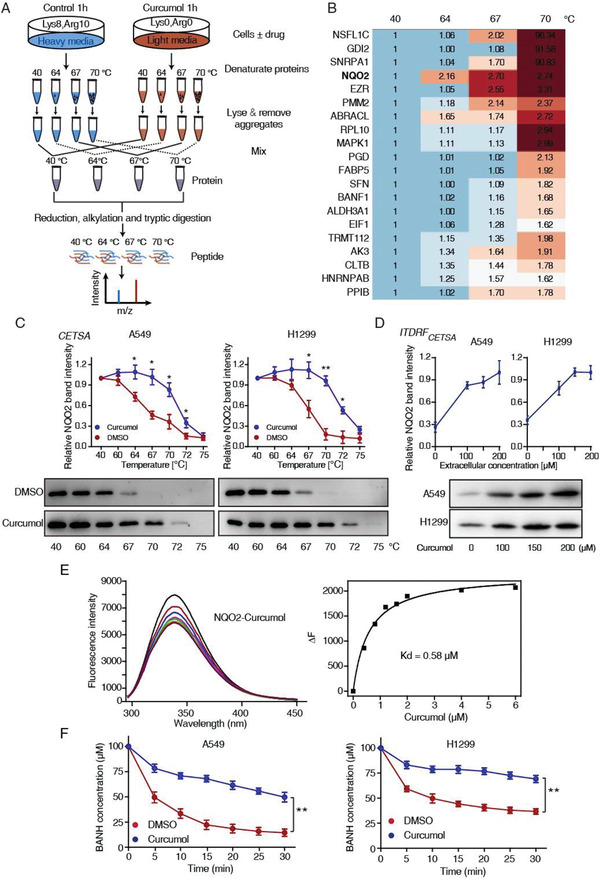
NQO2 is identified as a potential target of curcumol. A) Experimental design of the SILAC‐quantitative CETS profiling for identification of curcumol binding targets. B) Heatmap showed light/high ratio change in top 20 putative protein at different temperatures. C) CETSA curves comparing NQO2 thermal stability change between curcumol and control group in cell lysates. D) ^ITDRF^CETSA in lysates for NQO2 and curcumol at fixed temperature (67 °C). E) Fluorescence titration experiments determining the interaction between curcumol and NQO2. The fluorescence quenching at 330 nm verses curcumol concentration was fitted with Hill plot. F) Enzymatic activity of NQO2 in the presence or absence of curcumol was determined by measuring the absorbance of reduced BNAH. Bars, SEM; *n* = 3; *p < 0.05 or **p < 0.01.

We next set up a SILAC‐based CETSA profiling to identify the target protein that directly interacts with curcumol. The technology is based on the measurements of a remaining soluble target protein against the background of thermally denatured and precipitated proteins following a thermal alteration.^[^ ^18^ ^]^ In particular, its combination with SILAC allowed unambiguous identification of the target protein in local biological systems.^[^ ^19^ ^]^ To identify the target engagement of curcumol, A549 cells cultivated with “light media” or “heavy media” were treated with curcumol or DMSO for 1 h, and then denatured in increasing temperatures, the remained supernatants from “light” and “heavy” were mixed in 1:1 ratio and digested with trypsin, then subjected to mass spectrometry (MS) analysis (**Figure** **3**A). A total of 1147 proteins were identified (Table S2, Supporting Information); among the top 20 putative curcumol targets (Figure 3B), we focused our attention on NQO2, as its light/high ratio elevated in a temperature‐dependent manner. As confirmed by CETSA and isothermal dose‐response fingerprint analysis based CETSA (^ITDRF^CETSA) experiments, the thermal stability of NQO2 increased in curcumol‐treated group in temperature‐ and dose‐dependent manners (Figure 3C,D).

We then used fluorescence titration experiments to assess the direct physiological interaction between curcumol and NQO2 in vitro, and found that the addition of curcumol to the protein caused a significant fluorescence quenching with an equilibrium dissociation constant (Kd)  =  0.58  × 10^−6^ m (Figure 3E). Moreover, our BANH assay and Western blotting assay showed that curcumol could inhibit NQO2 enzymatic activity (Figure 3F), but not influenced the protein expression of NQO2 in both A549 and H1299 cells (Figure S3A, Supporting Information). In addition, we found that curcumol neither binds to quinone oxidoreductase NQO1 nor inhibits its enzyme activity (Figure S3B–D, Supporting Information), as indicated by fluorescence titration assay (Figure S3C, Supporting Information) and NQO1 enzyme activity detection (Figure S3D, Supporting Information). These results indicate that curcumol specifically binds to NQO2 and suppresses its enzymatic activity without affecting its expression level.

### 2.3 Curcumol Upregulates DR5 via Activating ROS‐ER Stress‐CHOP Signaling

**Figure 4:**
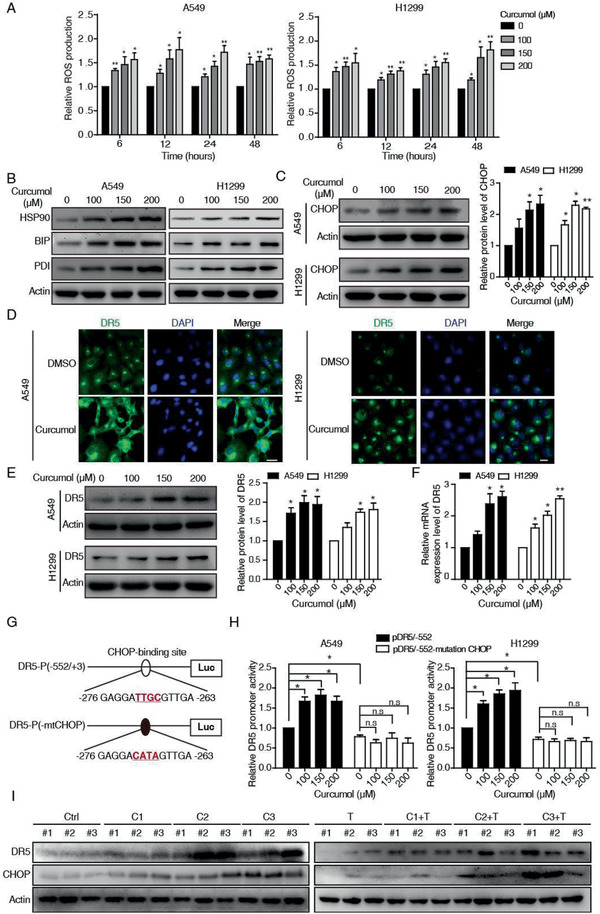
Curcumol induces ROS/ER stress and activates CHOP‐DR5 signaling. A) A549 and H1299 cells were incubated with indicated concentrations of curcumol for different time points, the intracellular ROS was detected by DHE assay. B) Both cell lines were treated with indicated concentrations of curcumol, western blotting was performed to determine ER stress‐related proteins including HSP90, BIP, and PDI, as well as CHOP C). D) Expression and cellular localization of DR5 change after curcumol treatment was analyzed by a fluorescence microscope; scale bar, 20 µm. E,F) Curcumol induced DR5 expression. Both A549 and H1299 cells were treated with elevating concentrations of curcumol for 24 h, and the expression of DR5 was determined by western blotting E) and qRT‐PCR F). G) Upper panel shows schematic structures of the DR5 promoter in the reporter plasmid containing CHOP binding sites. Lower panel shows the site‐specific mutations introduced in the reporter plasmid containing CHOP promoter. H) The luciferase activity in NSCLC cells transfected with pDR5‐WT or CHOP‐mutated pDR5 and then treated with curcumol (up to 20 × 10^−5^ m). I) Comparison of the expression levels of CHOP and DR5 among the tumors from mice treated with curcumol, TRAIL, or combination by western blotting analysis. Bars, SEM; *n* = 3; *p < 0.05 or **p < 0.01. n.s, not significant.

NQO2 is a flavin adenine mononucleotide (FAD)‐dependent quinone oxidoreductase that plays a critical role in cellular redox cycling and drug metabolism. It was reported that ROS could be generation when NQO2 directly binding with certain small molecule inhibitors.^[^ ^20^ ^]^ For example, imiquimod can target NQO2 and inhibit its activity, triggering ROS generation.^[^ ^21^ ^]^ We then hypothesized that curcumol may induce ROS‐ER stress in NSCLC cells. First, assay with dihydroethidium (DHE), a probe to detect cellular ROS including superoxide anion, hydrogen peroxide, and hydroxyl radical,^[^ ^22^ ^]^ showed that curcumol could induce ROS generation in a dose‐dependent manner (up to 20 × 10^−5^ m) (**Figure** **4**A). Interestingly, the level of ROS was increased moderately (fold change around 1.5) in each time points (up to 48 h), suggesting that curcumol stimulation could sustain a chronic ROS elevation in cancer cells (Figure 4A).

Recent studies have suggested that the ER stress induced by ROS plays an essential role in CHOP‐DR5 induction,^[^ ^4^, ^23^ ^]^ we thus investigated whether curcumol was capable to promote ER stress‐CHOP‐DR5 signaling. As shown in Figure 4B,C, ER‐stress associated markers including heat shock protein90 (HSP90), Immunoglobulin heavy chain binding protein (BIP), protein‐disulfide isomerase (PDI), and CHOP were elevated in both NSCLC cells treated with increasing concentrations of curcumol. Knockdown of CHOP could attenuate curcumol/TRAIL‐induced apoptosis in A549 and H1299, as supported by the rescue effect of cleaved‐PARP and cleaved‐caspase3 (Figure S4A,B, Supporting Information) and the accumulation of the Annexin‐V positive cell populations (Figure S4C, Supporting Information). These results suggest that CHOP is required for the anticancer effect of the curcumol/TRAIL treatment. As a target gene of CHOP, DR5 is critical for TRAIL‐induced apoptosis.^[^ ^24^ ^]^ Thus, we examined the effects of curcumol on DR5 expression. By using DR5 immunofluorescence staining and Western blotting analysis, we found that curcumol significantly increased the expression of DR5 in A549 and H1299 cells (Figure 4D,E). qRT‐PCR assay confirmed that the mRNA level of DR5 was promoted by the increasing treatment of curcumol (Figure 4F).

To further examine that the CHOP was required for curcumol‐induced DR5 transcriptional activation, we employed two luciferase reporter plasmids: pDR5/‐552 that contains a region (−552/+3) of the DR5 promoter sequence and a mutant version (pDR5/‐552‐mutation CHOP) that contains the same promoter sequence with mutation at the potential CHOP‐binding site (Figure 4G). NSCLC cells transfected with these plasmids were subjected to curcumol treatment, the results revealed that curcumol treatment increased DR5 promoter activity in A549 and H1299 cells transfected with the pDR5/‐552 plasmid but not in those transfected with the pDR5/‐552‐mutation CHOP (Figure 4H). These results indicate that CHOP directly mediates the curcumol‐induced upregulation of DR5. Functionally, knockdown of DR5 expression could attenuate the curcumol/TRAIL‐induced apoptosis (Figure S4D–F, Supporting Information). Moreover, upregulation of DR5 and CHOP was observed in the tumor xenograft tissues treated with increasing dosages of curcumol or in combination with TRAIL (Figure 4I). Taken together, curcumol activated the CHOP‐DR5 signaling to enhance the sensitivity of cancer cells to TRAIL.

### 2.4 ROS Regulated by NQO2 Sensitizes Cancer Cells to TRAIL‐Induced Apoptosis

**Figure 5:**
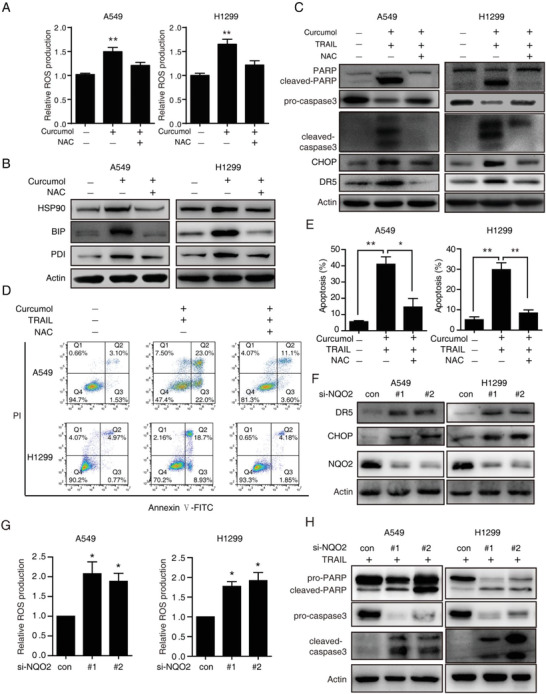
ROS regulated by NQO2 sensitizes cancer cell to TRAIL‐induced apoptosis. A–E) NSCLC cells were pretreated with or without 1 × 10^−3^ m NAC for 2 h, and then treated with or without curcumol and TRAIL cotreatment for 24 h, the intracellular ROS was detected A), ER stress‐related proteins B) and apoptosis‐related proteins C) were determined by western blotting, and apoptotic cells were analyzed by flow cytometry D,E). F,G) NSCLC cells were transfected with siRNA against NQO2 or scramble siRNA (si‐con) for 24 h, and the expression of DR5, CHOP, and NQO2 were determined by western blotting assay F) and cellular ROS was detected by DHE assay G). H) A549 and H1299 cells incubated with TRAIL were treated with si‐NQO2, then the cell apoptosis was determined by western blotting. Bars, SEM; *n* = 3; *p < 0.05 or **p < 0.01.

To determine whether ROS‐ER stress signaling is essential for the anticancer effect of curcumol/TRAIL in NSCLC, we introduced ROS scavenger *N*‐acetyl‐cysteine (NAC) to reduce the ROS levels enhanced by curcumol (**Figure** **5**A), and found that curcumol‐induced ER stress could be suppressed by NAC, as indicated by the expression of HSP90, BIP, and PDI (Figure 5B). Blockade of ROS could also prevent cancer cells from apoptosis that was induced by the co‐treatment of curcumol/TRAIL, as indicated by cleaved‐PARP, cleaved‐caspase3, and Annexin V‐FITC/PI assays (Figure 5C–E).

Interestingly, the expression of CHOP and DR5 enhanced by curcumol/TRAIL could also be reduced by NAC (Figure 5C), suggesting that ROS is a critical mediator in ER stress‐CHOP‐DR5 signaling. We then knocked down NQO2 using two specific si‐RNAs against NQO2, resulting in the significant upregulation of DR5, CHOP, and cellular ROS (Figure 5F,G). Moreover, knockdown of NQO2 in both A549 and H1299 cells could enhance apoptotic death in the presence of TRAIL (Figure 5H). Taken together, these results indicate that ROS regulated by NQO2 is a critical mediator in curcumol/TRAIL‐induced ER stress and apoptosis.

### 2.5 NQO2 is Associated with Poor Prognosis in Human NSCLC Cancer

**Figure 6:**
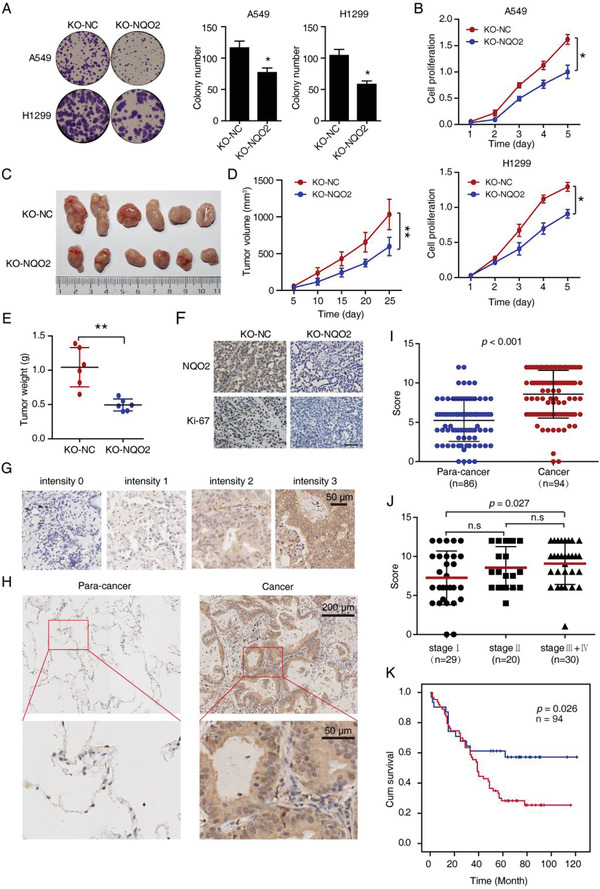
Clinical significance of NQO2 in NSCLC. A,B) The colony formation and cell proliferation assays were performed to compare the indicated cell lines with or without NQO2 knockout. Bars, SEM; *n* = 3; *p < 0.05. C) Subcutaneous tumor growth in nude mice after injection of indicated cells. Tumor volumes were measured every 5 days (*n* = 6 per group), and the growth curves and tumor weight were summarized in the below charts D,E). Bars, SD; *n* = 6; **p < 0.01. F) Ki‐67 staining of tumors and IHC staining for NQO2 protein in these excised tissues. Scale bar, 50 µm. G) The NQO2 protein levels in 94 NSCLC tissues were analyzed by IHC staining. H) The images represented differential staining intensities of NQO2. I) Representative results of IHC staining for NQO2 expression in cancer tissues and paired nontumor tissues. The relative NQO2 expression levels were analyzed based on scores, and the results were shown as means ± SD. J) The correlation between NQO2 expression and the clinical stage were analyzed between stage I, stage II, and stage Ш/IV. n.s, not significant. K) Kaplan‐Meier analysis of overall survival rate in 94 NSCLC patients according to their NQO2 expression levels.

In the basis of the fact that NQO2‐mediated cellular redox signaling is essential for sensitizing cancer cell to TRAIL‐induced apoptosis, we hypothesized that NQO2 plays a critical role in tumorigenesis and chemoresistance in NSCLC. Firstly, we generated NQO2 knockout (KO‐NQO2) cell lines with A549 and H1299 using Crispr/Cas9 system. Colony formation assay and WST‐1 assay showed that cell growth was slower in KO‐NQO2 cells than the corresponding control groups (**Figure** **6**A,B). To examine the effect of NQO2 in vivo, we used a BALB/C nude mouse model with xenografts formed by NQO2 knockout cells and the corresponding control groups. The KO‐NQO2 group exhibited significant smaller tumor volume and tumor weight than the control group (Figure 6C–E). Ki‐67 staining further confirmed that deletion of NQO2 suppressed cancer proliferation in vivo (Figure 6F).

We then investigated the clinical significance of NQO2 in NSCLC with a tissue microarray containing 94 NSCLC patient tissue samples by performing immunohistochemistry (IHC) staining (Figure 6G). IHC staining showed that NQO2 was significantly higher in NSCLC cancer tissues as compared with their adjacent normal tissues (Figure 6H,I). In addition, NQO2 score at clinical stages III and IV was higher than stage I (*p* = 0.027, Figure 6J), and there existed a significant correlation between tumor NQO2 expression level and the pathologic T stage (*p* = 0.037, Table S3, Supporting Information). Kaplan–Meier survival curve showed that patients with low NQO2 expression had a longer survival than the patients with high NQO2 expression (Figure 6K). Taken together, these results indicate that NQO2 may serve as a prognostic indicator for patients with NSCLC.

### 2.6 Curcumol Binds to NQO2 Near Its Active Sites

**Figure 7:**
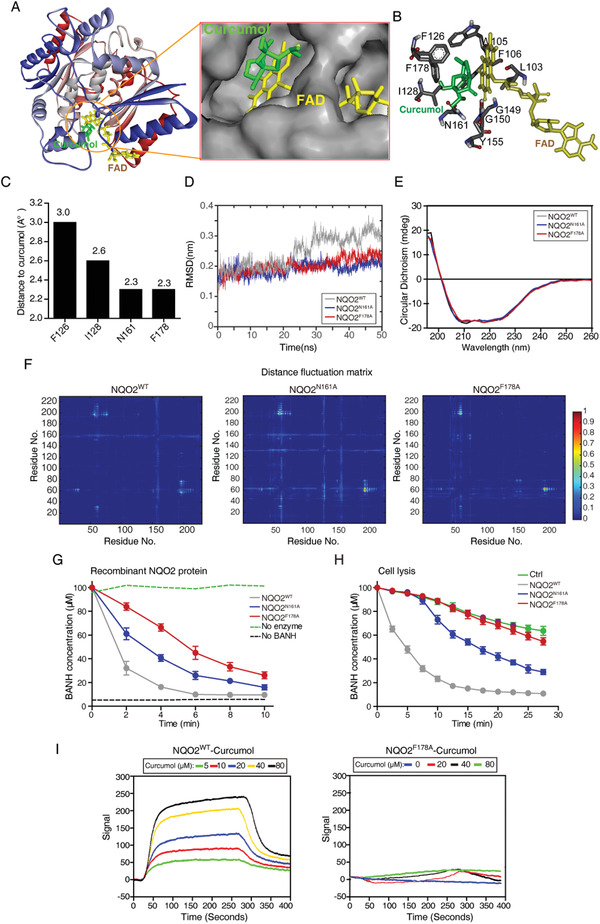
Curcumol binds near to NQO2 active pocket. A) Schematic representation of the interactions between NQO2 (PDB:1ZX1) and curcumol analyzed by AutoDock. B) Predicted interacting amino acids in NQO2 based on in silico docking assays. C) The distances between curcumol and predicted interacting amino acids were plotted. D) The time evolution of RMSD values for four NQO2 protein and mutants, wild type (gray), NQO2^N161A^ (blue), and NQO2^F178A^ (red). E) Secondary structures of NQO2^WT^ and mutants (NQO2^N161A^ and NQO2^F178A^) were determined by CD spectrometry. F) Distance fluctuation matrices for NQO2 in all different mutation states. The magnitude of pairwise distance fluctuations is color coded from blue (small fluctuations) to red (large fluctuations). G) In vitro NQO2 enzymatic activity detection. In the presence of substrate K3 (menadione, 10 × 10^−5^ m), co‐substrate BNAH (10 × 10^−5^ m), and recombinant mutant NQO2 proteins (100 ng), the decrease of BNAH fluorescence was measured by fluorimetry. H) Cellular NQO2 enzymatic activity detection. NQO2‐KO cells was reintroduced with control, NQO2^WT^, NQO2^N161A^, or NQO2^F178A^, respectively, and the NQO2 reaction progress in the presence of substrate K3, BNAH, and A549 cell lysate (2.5 µg) with indicated forms of NQO2 mutant was determined by measuring the absorbance of reduced BANH. I) Representative surface plasmon resonance (SPR) sensorgram for the association of curcumol (0‐80 × 10^−6^ m) to immobilized NQO2^WT^ and NQO2^F178A^.

Knowing the direct inhibitory effect of curcumol on NQO2, we next investigated the interaction between NQO2 and curcumol in detail. By performing molecular docking with AutoDock, the pose with the lowest binding free energy and the highest scoring orientation was selected for further analysis (Figure S4A,B, Supporting Information). In silico docking analysis revealed that curcumol, with two carbocyclic rings, binds adjacent to the isoalloxazine rings of FAD through hydrophobic bonding interactions with Phe126, Ile128, Asn161, Phe178 amino acid residues in the catalytic pocket of NQO2 (**Figure** **7**A,B). Among these four potential binding sites, Asn161 and Phe178 showed spatially closer to curcumol with distance of 2.3 Å (Figure 7C), suggesting a higher possibility for curcumol bonding.

We then explored the dynamic stability alteration of NQO2 protein by mutating the two amino acid residues individually. We first simulated the NQO2 carbon skeleton structure with different mutations, and the results showed that mutation of Asn161 or Phe178 did not significantly change the root mean square fluctuation (RMSF) values and the minimum distances between amino acids (Figure 7D and Figure S5C, Supporting Information). Circular dichroism (CD) spectrometric analysis also confirmed that mutating each of the two residues did not significantly influence the secondary structure of NQO2 protein (Figure 7E). Then, we subjected the recombinant mutant proteins NQO2^N161A^ and NQO2^F178A^ to fluorescence titration experiments, and found that either one of the two mutants did not interact with curcumol (Figure S5D,E, Supporting Information). These results indicate that either Asn161 or Phe178 residue in NQO2 protein is structurally equally important in the contribution to the curcumol binding.

To ask which residue is more critical for curcumol binding influencing the protein activity, we analyzed the molecular dynamics (MD) with simulation trajectories of the two mutants, and found that the two mutants of NQO2 displayed distinct fluctuation, as compared to wild type (WT) (Figure 7F). We then tested the enzymatic activity of the two NQO2 mutants in purified recombinant proteins and A549 cell lysates, and found that the F178A mutation showed more inhibitory effect than the N161A mutation in both assays (Figure 7G,H). Moreover, SPR assay revealed the curcumol binding with NQO2^WT^ in a dose‐dependent manner; however, the recombinant NQO2^F178A^ protein presented no significant signal along with increasing concentrations of curcumol (Figure 7I). Taken together, our data verified that Phe178 is an important amino acid residue for both NQO2 enzymatic activity and its binding with curcumol.

### 2.7 NQO2 Mediates TRAIL Resistance In Vivo by Modulating ROS Level

**Figure 8:**
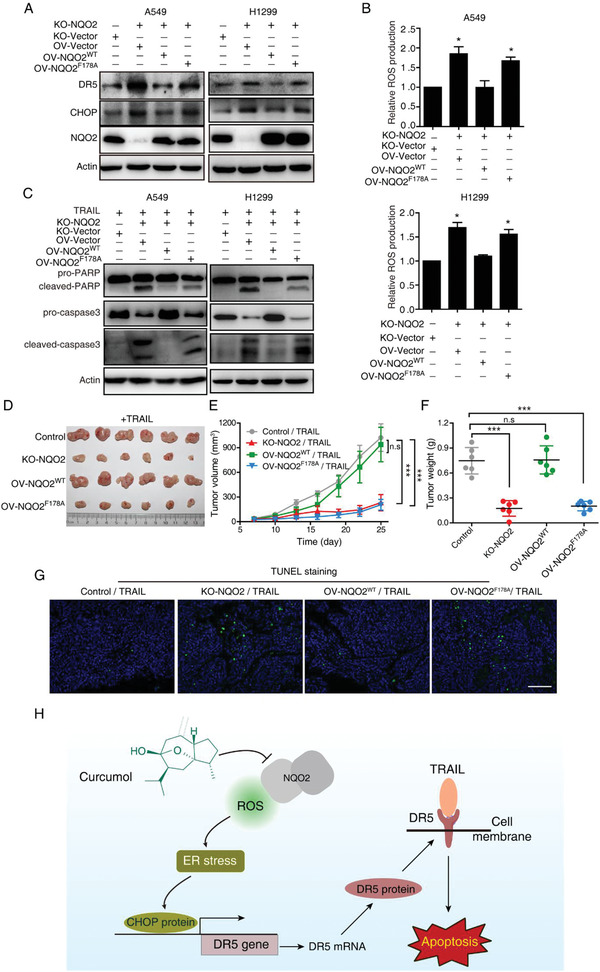
Deactivated NQO2 sensitizes cancer cell to TRAIL‐induced apoptosis. A) NQO2‐KO cells were reintroduced with NQO2^WT^ or NQO2^F178A^, in the presence of TRAIL, the expression of CHOP and DR5 was detected by immunoblotting and the ROS level was detected by DHE assay. Bars, SEM; *n* = 3; *p < 0.05. C) Western blotting analysis of apoptosis‐related proteins in the indicated cells treated with TRAIL. D) Xenograft experiment was performed in BALB/c nude mice injected with A549 expressing indicated proteins, and administrated with TRAIL 6 days later. Xenograft tumor growth E) and tumor weight F) were statistically plotted. Bars, SD; *n* = 6; ***p < 0.001. n.s, not significant. G) The tumor tissues excised from each group were subjected to TUNEL staining, Scale bar, 50 µm. H) Working model illustrating that curcumol sensitizes TRAIL‐induced apoptosis by targeting NQO2.

To evaluate whether NQO2 inhibition efficiently sensitizes cancer cells to TRAIL‐induced apoptosis, we re‐expressed NQO2^WT^ and NQO2^F178A^ in KO‐NQO2 A549 and H1299 cells. As shown in **Figure** **8**A, knockout of NQO2 upregulated DR5, CHOP, and cellular ROS level, and these effects could be rescued by the re‐expression of NQO2^WT^ but not NQO2^F178A^ (Figure 8A,B). Consistently, cells with KO‐NQO2 and NQO2^F178A^ re‐expression were sensitive to TRAIL‐induced apoptosis, while KO‐NQO2 cells with NQO2^WT^ re‐expression showed resistance to TRAIL (Figure 8C).

To examine the effect of NQO2^F178A^ in vivo, A549 cells with KO‐NQO2 and re‐expression of NQO2^WT^ or NQO2^F178A^ were subcutaneously implanted into the nude mice and treated with TRAIL. The results showed that, similar to KO‐NQO2, NQO2^F178A^ group exhibited more sensitive to TRAIL treatment, as indicated by the tumor growth curve, tumor weights, and TUNEL staining (Figure 8D–G). In conclusion, curcumol targets NQO2 and engages a ROS‐driven ER stress‐CHOP‐DR5‐dependent pathway to overcome TRAIL resistance of NSCLC (Figure 8H).

## 3 Discussion

Curcumol is a major sesquiterpenoid hemiketal compound isolated from the herbal medicine plant *Rhizoma Curcumae*. Inspired by its remarkable anti‐inflammatory activity,^[^ ^8^ ^]^ we identified curcumol as a novel safe sensitizer for TRAIL‐resistant NSCLC cells. In combination with TRAIL, curcumol exhibits synergistic lethal effects on NSCLC via interacting with NQO2 to activate ROS‐CHOP‐DR5 signaling.

Identification of drug targets has always been a hot spot and challenge in clinical cancer research. Current identification strategies, such as stability of proteins from rates of oxidation (SPROX), drug affinity responsive target stability (DARTS), and activity‐based protein profiling (ABPP), can only be suitable for part of specific compounds.^[^ ^25^ ^]^ Taking the advantage of the cellular thermal shift assay, we first integrated it into SILAC‐based proteomics to measure the melting transitions of drug target engagement. From this, we precisely identified that curcumol directly binds to NQO2 with residue Phe178 as the primary functional ligand, sustaining an oxidative stress and activating ROS‐CHOP‐DR5 signaling to overcome TRAIL resistance in cancer therapy of NSCLC.

NQO2 was reported to catalyze the metabolic reduction of quinones and provides cellular protection against exogenous toxic agents in cancer cells.^[^ ^26^ ^]^ For example, NQO2 was reported to catalyze the reduction of endogenous biological substrates estrogen quinones and melatonin, playing a critical role in cellular detoxification.^[^ ^27^ ^]^ However, Miettinen et al. reported that NQO2 is an off‐target for acetaminophen, by which it adopts acetaminophen as a substrate to derive ROS generation, suggesting that NQO2‐mediated metabolism can induce ROS burst when appropriate substrates are available.^[^ ^20^ ^]^ Several other exogenous substrates, including resveratrol^[^ ^28^ ^]^ and quercetin,^[^ ^29^ ^]^ exhibit prooxidant roles by inhibiting the enzyme activity of NQO2. Imiquimod, a small‐molecule ligand of Toll‐like receptor‐7, is able to interact with NQO2 and inhibit its activity, which subsequently triggers ROS burst.^[^ ^21^ ^]^ Our current experiments demonstrated that curcumol can also interact with NQO2 directly to induce ROS signaling.

The isoalloxazine ring of FAD and surrounding amino acid residues are critical for the catalytic pocket of NQO2.^[^ ^30^ ^]^ A previous study proposed that imatinib and nilotinib may compete with FAD for suppressing the enzymatic activity of NQO2.^[^ ^31^ ^]^ However, our docking analysis showed that curcumol bound adjacent to the catalytic pocket but not influenced FAD. Two residues, Asn161 and Phe178, play roles in the attachment with curcumol (Figure 7C and Figure S5E, Supporting Information). However, mutation of Phe178 exhibited more profound inhibitory effects on the enzymatic activity of NQO2 (Figure 7G,H), suggesting that Phe178 is more critical for the detainment of substrates that enter the catalytic pocket.

NQO2 was reported to participate in numerous human diseases, including cancer^[^ ^32^ ^]^ and neurodegenerative diseases.^[^ ^33^ ^]^ Previous study showed that loss of NQO2 displayed slower proliferation and G1 phase cell accumulation in prostate cancer.^[^ ^34^ ^]^ The regulation of NQO2 on ROS led us to postulate its oncogenic role in NSCLC. Here, we demonstrated that knock out of NQO2 inhibited cell growth in vivo and in vitro (Figure 6A–F). IHC staining in 94 pairs of NSCLC tissues further confirmed an inverse correlation between NQO2 and overall survival (Figure 6G–K), suggesting NQO2 might have therapeutic properties based on its clinical significance in NSCLC. We further focused on the role of NQO2 in CHOP‐DR5 signaling and how it mediates TRAIL resistance. The cellular ROS level is a critical mediator for DR5 expression, our in vivo and in vitro results showed that cell lines with NQO2 deletion or inactivated mutation (F178A) exhibited ROS generation, which was an essential signal for sensitizing cancer cells to TRAIL exposure via activating CHOP‐DR5 pathway (Figure 8).

It is gratifying to observe that curcumol‐treatment causes synergistic lethal effects with TRAIL on NSCLC. Considering its long history of usage in daily diet, we believe that curcumol may serve as an ideal candidate drug in chemoprevention. Curcumol belongs to the family of sesquiterpenoid with large members. It remains to be explored whether the NQO2 activation by curcumol is shared by other sesquiterpenoids with even higher potency and better selectivity. Interestingly, we found that curcumol stimulation induced chronic oxidative stress and ROS generation, which may not be sufficient enough to activate intrinsic cell death but properly triggered CHOP‐DR5 signaling (Figure S1B,C, Supporting Information, Figure 4). This could explain why curcumol at the current low dose had nontoxic effect on cancer cells but showed synergistic lethal effects in combination with TRAIL.

Recent studies have shown that DR5 upregulation by chemotherapy agents could overcome TRAIL resistance,^[^ ^24^, ^35^ ^]^ and ER stress can induce the increase of DR5 expression in a wide spectrum of human cancers via transactivation of the transcription factor CHOP.^[^ ^36^ ^]^ CHOP binds to 5’ untranslated region of DR5 promoter to initiate DR5 expression in response to ER stress inducers. This finding provides a clue for developing TRAIL‐sensitizing agents. Many ROS inducers, such as thapsigargin,^[^ ^37^ ^]^ proteasome inhibitors (MG132 and ninlaro),^[^ ^38^ ^]^ and phytocompounds^[^ ^39^ ^]^ were reported to exhibit anticancer effects in combination with TRAIL. However, their stability and safety for the clinical application remain to be the obstacle. Seeking novel chemotherapeutics from food‐source compounds for synergistic treatment with TRAIL is a promising strategy to guarantee the safety and effectiveness. In this study, we successfully identified curcumol as a potential TRAIL‐receptor agonist from a food‐source compound library. Our in vitro and in vivo studies provided solid evidences that curcumol directly regulates NQO2 through its catalytic site to induce CHOP‐DR5 signaling, exerting synergistic lethal effects with TRAIL on NSCLC.

Cancer cell resistance to TRAIL‐induced apoptosis remains a significant factor in the failure of clinical trials, suggesting that a TRAIL‐comprising therapy will only be effective when a potent TRAIL sensitizer is applied. Based on our data, we propose that curcumol directly suppresses NQO2 enzyme activity to cause ROS generation and ER stress, triggering DR5 expression via CHOP dependent manner. This discovery highlights the potential of using curcumol to target NQO2 for clinical treatment of TRAIL‐resistant cancers.

## 4 Experimental Section

##### In Vivo Tumorigenicity Experiments

BALB/c nude mice (5‐week‐old) were subcutaneously inoculated with A549 cell (1 × 10^6^). After solid tumor reached ≈5 mm diameter, the tumor‐bearing mice were randomly divided into eight groups (*n* = 6 per group). The mice of TRAIL treatment groups were intratumorally injected with TRAIL (100 µg per mouse) every 3 days, and the mice of curcumol treatment groups were intragastrically treated with curcumol dissolved in corn oil (100 µL per injection) every 2 days. Mice were treated for a period of 15 days. The eight groups of mice were assigned to receive the following treatments: control group, vehicle only; TRAIL group, TRAIL only; three curcumol groups, curcumol of low (2 mg kg^−1^), medium (10 mg kg^−1^), and high (50 mg kg^−1^) dosage, respectively; three curcumol/TRAIL combination treatment groups, TRAIL (100 µg per mouse) plus curcumol with 2, 10, and 50 mg kg^−1^ dosage, respectively. The tumor diameters and mice body weight were measured every 3 days, and the volumes were calculated according to the following formula: Volume (mm^3^) = length × (width)^2^/2. At the end of the experiment, blood was collected for determining biochemical and hematological parameters, tumors were harvested for Western blotting and TUNEL assay, organs including liver, kidneys, lung, and heart were harvested for histologic analyses.

To investigate the role of NQO2 in cell proliferation, the established NQO2‐KO or NQO2‐WT A549 cells (1 × 10^6^) were subcutaneously injected into dorsal flank of each mice, and the tumors were measure every 3 days. For the experiment of comparing the effect of NQO2‐F178A and NQO2‐WT in TRAIL treatment, the indicated cell lines were subcutaneously injected into the axilla of each mice. After solid tumor reached ≈5 mm diameter, each group was administrated with TRAIL (100 µg per mice) every 3 days for a period of 15 days. All the animal experiments were approved by the Committee on the Use of Live Animals in Teaching and Research, Jinan University (Project number: 20181211‐03/20191125‐06).

##### Cellular Thermal Shift Assay

CETSA was performed according to Attarha et al.^[^ ^40^ ^]^ described with minor modifications. Cells were treated with curcumol (20 × 10^−5^ m) or DMSO for 1 h and then harvested, and suspended in PBS supplemented with protease inhibitor cocktail (Sigma, St. Louis, MO, USA) and PMSF (Beyotime). The respective lysates were divided into 50 µL aliquots and then heated at the indicated temperature for 3 min and followed by cooling for 5 min at room temperature. Then the samples were lysed with three freeze–thaw cycles using liquid nitrogen and a 25 °C water bath. To separate the soluble fractions, the heated lysates were centrifuged at 16 000 × *g* for 15 min at 4 °C. Soluble proteins in the supernatants were analyzed by Western blot analysis, and the intensity of bands was normalized to the lowest temperature bands. ^ITDRF^CETSA experiments were performed with different concentration of curcumol at constant temperature of 67 °C.

##### SILAC‐Based Quantitative Proteomics

A549 cells were labeled as previously described.^[^ ^14^ ^]^ The “light (Lys0, Arg0)” labeled A549 cells were treated with 20 × 10^−5^ m curcumol for 1 h, and the “heavy (Lys8, Arg10)” labeled A549 cells were treated with DMSO, then proteins were extracted based on CETSA at temperatures 40, 64, 67, and 70 °C. A pair of DMSO and curcumol treated samples at the same temperature were mixed at equal amounts. Then the mixed protein samples were subjected to the in‐solution digestion as previously described.^[^ ^15^, ^41^ ^]^ The digested peptides were desalinated using a MonoTIPTM C18 Pipette Tip (GL Sciences, Tokyo, Japan) and then analyzed with an Orbitrap Fusion Lumos mass spectrometer (Thermo Fisher Scientific) as previously described.^[^ ^42^ ^]^

The raw MS data files were searched against UniProt‐Swiss Human database (2017_03 Release) using Proteome Discoverer v2.1 (Thermo Fisher Scientific). Search parameters: 1) quantification methods, SILAC 2 plex (Arg10, Lys8); 2) MS tolerance, 10 ppm; acid; 3) digestion, trypsin; 4) protein FDR, 0.01; 5) dynamic modifications, oxidation (+15.995 Da) of methionine, deamination (+0.984 Da) of Gln and Asn, and acetyl (+42.011 Da) of the N‐terminus; 6) static modifications, carbamidomethyl (Cys, +57.021 Da).

##### Molecular Docking and Dynamics Simulation

Curcumol binding to NQO2 structure (PDB: 1ZX1) was modeled by AutoDock v4.2 and AutoDock Tools MGL v1.5.6. For protein preparation, all water molecules were removed, hydrogen atoms were added, and the NQO2 with bound FAD was utilized for docking. The curcumol ligand was generated by Chemdraw and Chem3D, and minimized using the MM2 force field. The prepared protein and ligand structures were saved in the PDBQT file format. The dimension of the grid box was calculated by AutoGrid program, and the grid box size was set to 90 × 90 × 90 with a spacing of 0.375 Å. The Lamarckian genetic algorithm was performed for ligand conformational searching. During the docking process, NQO2 was rigid, curcumol was flexible, trials of 500 runs were set, and other parameters were maintained at their default setting. The conformer with the lowest binding free energy and the highest scoring orientation was used for further analysis, and the results were visualized and rendered by PyMOL.

The mutation formats of NQO2 were generated by modifying the corresponding sequences and homology modeling methods (Discovery Studio, v.16). MD simulations and energy minimization of NQO2^WT^, NQO2^N161A^, and NQO2^F178A^ proteins were conducted by the Gromacs (v. 2019.1) software with GROMOS96 force field in explicit solvent model. To provide a stable conformation, energy minimized with steepest descent method consisted of up to 50 000 steps. The solvated system was equilibrated as follows: The canonical ensembles equilibration phase and the isobaric–isothermal ensembles equilibration phase were performed for 100 ps. After these equilibration procedures, MD simulations were performed at these three proteins for 50 ns, respectively. The results of these simulations were analyzed by calculating the root‐mean‐square deviation (RMSF). Minimum distance and distance fluctuation matrix of these protein structures were acquired from each MD trajectory on the time interval 20–50 ns.^[^ ^43^ ^]^

##### Protein Expression and Purification

Full‐length human NQO1, NQO2, or mutation‐NQO2 was cloned into the GST fusion vector pGEX‐4T‐1. The expression plasmids were transfected into *Escherichia coli* BL21 and the cells grown in LB medium with ampicillin (100 µg mL^−1^) at 37 °C, until the optical density at 600 nm (OD600) reached 0.6–0.8. To induce protein expression, 0.5 × 10^−3^ m isopropyl‐*β*‐d‐thiogalactoside (IPTG) was added and continued at 37 °C for an additional 6 h culture. Bacteria were harvested by centrifugation, resuspended in binding buffer (20 × 10^−3^ m Na_3_PO_4_·12 H_2_O, 0.5 m NaCl, pH = 7.4) and lysed by a constant systems cell disruptor (Constant Systems Ltd). Protein purification was carried out by GST‐affinity column (Glutathione Sepharose 4B, GE Healthcare). The column was washed with elution buffer (10 × 10^−3^ m GSH, 50 × 10^−3^ m Tris‐HCl), and then elution buffer was exchanged to binding buffer, the protein was concentrated to 5 mg mL^−1^ with centrifugal filter (10 K, Merck Millipore). The fusion proteins were digested with thrombin (GE Healthcare) in PBS (pH = 7.4) overnight at 25 °C. The cleaved GST‐tag was removed by GST‐affinity column.

##### NQO2 Enzymatic Activity Assays

Inhibition of NQO2 enzymatic activity was determined as previously described.^[^ ^26^ ^]^ Collected cells were resuspended in reaction buffer (50 × 10^−3^ m Tris/HCl, pH 7.5, 1 × 10^−3^ m n‐octyl‐*β*‐d‐glucopyranoside) and homogenized on ice. 2.5 µg of freshly prepared cell homogenates or 100 ng recombinant NQO2 were added to 50 µL reaction buffer with varying concentrations of test compounds and rested for 5 min at room temperature. Enzymatic reactions were initiated by adding 100 µL of reaction buffer containing 15 × 10^−5^ m of 1‐benzyl‐1,4‐dihydronicotinamide BNAH as co‐substrates along with 15 × 10^−5^ m menadione. Absorbance of the samples was measured using a plate reader with excitation at 340 nm and emission at 465 nm for 30 min at room temperature and normalized to a spontaneous decay in BNAH fluorescence in the absence of cell homogenate.

##### Fluorescence Spectroscopy

Fluorescence spectra were collected at 37 °C by using a fluorescence spectrometry (Hitachi F7000). Instrument parameters: excitation wavelength, 280 nm; emission wavelength start, 290 nm; emission wavelength end, 500 nm; scan speed, 1200 nm min^−1^; and PMT voltage, 630 V. Both purified proteins (4 × 10^−6^ m) and curcumol (2.4 × 10^−3^ m stock solution) samples were dissolved in PBS (pH = 7.4). The aliquots of curcumol were gradually added to 1.5 mL NQO2 solution, and the fluorescence spectra were recorded. The resulting titration data were fitted with the Hill plot equation *y* = *V*_max_ × *x*_n_/(k_n_ + *x*_n_), and the dissociation constant (K_d_) was calculated by Origin 8.5 software.^[^ ^44^ ^]^

##### Circular Dichroism (CD) Spectroscopy

To compare the secondary structure of WT and mutants NQO2, CD experiments were performed with a CD spectrometer (Chirascan, Applied Photophysics Ltd., Leatherhead, UK) at room temperature. CD data were collected for 5 × 10^−6^ m NQO2^WT^, NQO2N^161A^, and NQO2^F178A^ in PBS (pH 7.4) using a quartz cuvette with a 0.1 cm optical path length. The scanning wavelength was 195–260 nm at a 100 nm min^−1^ scan rate with 1 nm bandwidth. Each scan was measured three times and the secondary structures of proteins were analyzed by CDPro software.

##### Surface Plasmon Resonance (SPR)

To examine the binding of curcumol to NQO2, SPR was performed on an OpenSPR system (Nicoya Lifesciences Inc., Kitchener, Canada). In brief, protein was immobilized on sensor chips. Subsequently, curcumol solution was introduced into the sensor chip with PBS as the running buffer.

##### Immunohistochemistry

IHC assays were performed as previously described using anti‐NQO2 antibodies (1:2000).^[^ ^45^ ^]^ A human tissue microarray containing 94 NSCLC patient tissues samples, including 94 cancer tissues and 84 adjacent normal tissues, is randomly chosen from the Biobank of National Engineering Center for Biochip at Shanghai (Outdo Biotech, Shanghai, China). The experiment was approved by Committee on the Shanghai Outdo Biotech Company (Project number: T19‐0210). NQO2 immunostaining was evaluated based on scores representing the percentage of positively stained tumor cells and the staining intensity. The scale of positively stained cells: 0, 0–10%; 1, 10–30%; 2, 30–50%; 3, 50–80%; and 4, 8–100% and the staining intensity: 0, no staining; 1, slight staining; 2, moderate staining; 3, strong staining. Scores for the staining proportion and the staining intensity were multiple. The NQO2 expression level was considered high (>6) or low (≤6) based on the final scores.

##### Statistical Analysis

Two‐tailed Student's tests, one‐way ANOVA analysis, two‐way ANOVA analysis, and Chi‐square test were performed using GraphPad Prism software v.5.01. All in vitro experiments were repeated at least three times, and the values were presented as the mean ± SEM, apart from animal experiments using mean ± SD. The differences with *p < 0.05, **p < 0.01, or ***p < 0.001 were considered statistically significant. Survival analysis was performed using the Kaplan–Meier method with the log‐rank test by IBM SPSS Statistics v.19 (SPSS Inc., Chicago, IL).

Other methods are provided in Supplementary Materials and Methods, Supporting Information, including the following: 1) reagents and chemicals; 2) cell lines and culture conditions; 3) cell viability assay; 4) flow cytometric analysis; 5) immunoblotting; 6) luciferase reporter assay; 7) confocal; 8) qRT‐PCR analysis; and 9) transfection and generation of stable cell lines.

## Conflict of Interest

The authors declare no conflict of interest.

## Supporting information

Supporting information
