# Supplementary material for: Curcumol Overcomes TRAIL Resistance of Non‐Small Cell Lung Cancer by Targeting NRH:Quinone Oxidoreductase 2 (NQO2)
Source: Adv Sci (Weinh). 2020 Oct 15;7(22):2002306. doi: 10.1002/advs.202002306 (PMC7675185; doi:10.1002/advs.202002306)
Supplement: Supplementary file 1 — Supporting information [file ADVS-7-2002306-s001.pdf]

© 2020 Wiley-VCH GmbH

ADVANCED SCIENCE
Open Access

Supporting Information

for *Adv. Sci.,* DOI: 10.1002/advs.202002306

Curcumol Overcomes TRAIL Resistance of Non-Small Cell Lung Cancer by Targeting NRH:Quinone Oxidoreductase 2 (NQO2)

Jing Zhang, Ye Zhou, Nan Li, Wan-Ting Liu, Jun-Ze Liang, Yue Sun, Wei-Xia Zhang, Run-Dong Fang, Sheng-Ling Huang, Zheng-Hua Sun, Yang Wang, and Qing-Yu He**

Curcumol Overcomes TRAIL Resistance of Non-Small Cell Lung Cancer by Targeting NRH:Quinone Oxidoreductase 2 (NQO2)

Jing Zhang[1,2], Ye Zhou[1], Nan Li[1], Wan-Ting Liu[1], Jun-Ze Liang[1], Yue Sun[1], Wei-Xia Zhang[1], Run-Dong Fang[1], Sheng-Ling Huang[2], Zheng-Hua Sun[1], Yang Wang[1]*, Qing-Yu He[1,2,]*

Supplementary materials and methods

1) Reagents and chemicals

Recombinant TRAIL was purchased from PeproTech and Sino Biological Inc. (Beijing, China), curcumol (purity ≥ 98%) from Must Bio-Technology Co. (Chengdu, China), Z-VAD-FMK from Selleck (Houston, TX, USA). *N*-acetyl-L-cysteine (NAC) and dihydroethidium (DHE) were purchased from Beyotime (Jiangsu, China). Antibodies: pro-PARP, cleaved-PARP, pro-caspase3, cleaved-caspase3, CHOP, BIP and PDI were purchased from Cell Signaling Technology (Danvers, MA, USA). DR5, NQO2, HSP90, p53, β-actin and the HRP-conjugated goat anti-rabbit/mouse secondary antibody were purchased from Proteintech (Wuhan, Hubei, China).

2) Cell lines and culture conditions

Cell lines including A549, NCI-H1299, NCI-H460, NCI-H358 and NCI-H1975 were obtained from the American Type Culture Collection (Manassas, VA, USA). All cell lines were maintained in complete DMEM (Thermo Fisher Scientific, Waltham, MA, USA) supplemented with 10% fetal bovine serum (FBS, Gibco-Invitrogen Corporation, CA), 1% penicillin/streptomycin (GBCBIO Technologies, Guangzhou, China) and 10 μg/mL ciprofloxacin.

3) Cell viability assay

Cytotoxicity of curcumol, TRAIL, or combination treatment of curcumol and TRAIL was evaluated by WST-1 assay (Beyotime) according to the manufacturer's instructions. To analyze long-term survival (Clonogenic assay), cells were seeded in 12-well or 6-well plates at a density of 1000 or 2000 cells per well, correspondently, and cultured for 4 days, then, cells were exposed to various concentrations of curcumol or/and TRAIL for another 8 days. The colonies were stained with 1% crystal violet, and the number of colonies were counted using ImageJ software (version 1.44I).

4) Flow cytometric analysis

For apoptosis detection, apoptotic cells were stained with Annexin V-FITC/PI apoptosis detection kit (Vazyme Biotech, Nanjing, China), and the apoptotic cells were determined by C6 flow cytometry (BD Biosciences, San Diego, CA). For ROS detection, cells were stained with DHE (0.5 mg/ml, Beyotime) for 20 min at 37 °C. The absorbance was measured with a multifunctional microplate reader Spark 10M (Tecan, Synergy-HT).

5) Immunoblotting

Protein extractions were subjected to SDS-PAGE, followed by transfer onto PVDF membranes (BIO-RAD, Hercules, CA, USA). The membranes were incubated with appropriate primary and secondary antibodies. The gel bands were visualized with the ECL reagent (BIO-RAD), and the images were obtained using a Tannon 5200-Multi (Tanon Science & Technology Co.,Ltd, Shanghai, China).

6) NQO1 enzymatic activity assay

For NQO1 enzymatic activity determination, 5 μg of freshly prepared cell homogenates or 50 ng

recombinant NQO1 were added to 50 μL reaction buffer (50 mM Tris/HCl, pH 7.5, 1 mM n-octyl-β-D-glucopyranoside) with varying concentrations of test compounds and rested for 5 min at room temperature. Enzymatic reactions were initiated by adding 100 μL of reaction buffer containing 300 μM NADH (Beyotime) as co-substrates along with 300 μM MTT and 150 μM menadione, then the absorbance of the samples was measured at 560 nm for 30 min using a plate reader. Dicoumarol (Targetmol, Boston, MA, USA) was used as positive control.

7) Luciferase reporter assay

Cells were co-transfected with renilla luciferase plasmid pRL-TK and firefly luciferase plasmid pGL3-Enhancer containing the DR5 promoter region. After 24 h of transfection, the cells were treated with curcumol or DMSO for another 24 h. The luciferase activity was measured by using dual-luciferase reporter assay (Promega, Madison, WI, USA) according to the manufacturer's instructions. Firefly luciferase activity was normalized to renilla luciferase activity.

8) Confocal

NSCLC cells were plated on glass coverslips for 24 h, then treated with curcumol or DMSO. These cells were fixed with 4% paraformaldehyde, permeabilized with 0.1% Triton X-100, incubated with primary antibody against human DR5 (CST) and subsequently incubated with the FITC-conjugated secondary antibodies (CST). Cellular nuclei were stained with DAPI (Beyotime), and fluorescences were directly visualized and recorded with a Zeiss LSM710 confocal microscope (Jena, Germany).

9) qRT-PCR analysis

Total RNA was extracted using TRIzol reagent (Thermo Fisher Scientific) and cDNA was generated using the PrimeScript Reverse Transcriptase (Takara Biomedical Technology, Beijing, China).

qPCR was performed using a SYBR green mixture (Bio-Rad) and Applied Biosystems StepOne Real-Time PCR System (Thermo Fisher Scientific). Primers are listed in Table S4.

10) Transfection and generation of stable cell lines

We generated vectors expressing shRNA sequences targeting CHOP and DR5 in the lentiviral pLKO.1 vector, respectively. To establish stable cell lines with NQO2 knockout, we selected effective sgRNA sequences for NQO2 knockout with CRISPR/Cas9. Stable cell lines with expression of wild-type or mutant NQO2 were established based on retrovirus vectors. Briefly, 293T cells were co-transfected with indicated expression vectors and virus skeleton vectors by using Lipofectamine 3000 (Thermo Fisher Scientific). A549 and H1299 cells were infected with lentiviral supernatants, and were selected under 1 μg/mL puromycin. Stably transfected cells were validated by immunoblotting analysis.

Supplementary Figures

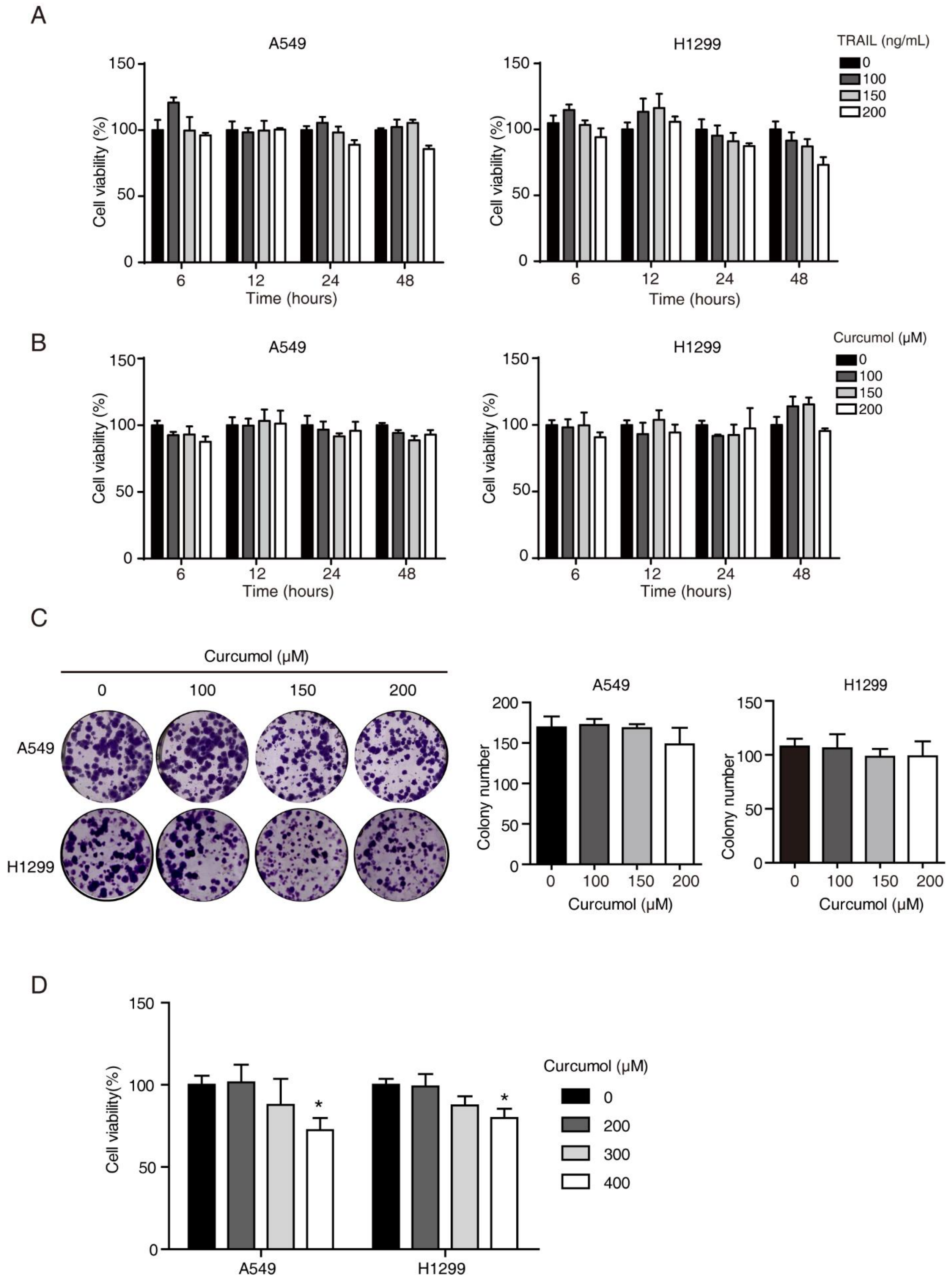

Figure S1. The cytotoxicity of curcumol or TRAIL in NSCLC. (A, B) A549 and H1299 cells were incubated with elevating concentrations of TRAIL (up to 200 ng/mL) or curcumol (up to 200 μM) for indicated time points (up to 48 h), the cell viability was determined by WST-1 assay. (C) A549 and H1299 cells were treated with indicated concentrations of curcumol, and their abilities to form colonies were statistically presented. (D) A549 and H1299 cells were incubated with higher concentrations (200, 300 and 400 μM) of curcumol for 48 h, the cell viability was then determined by WST-1 assay. Bars, SEM; N = 3; **P* < 0.05.

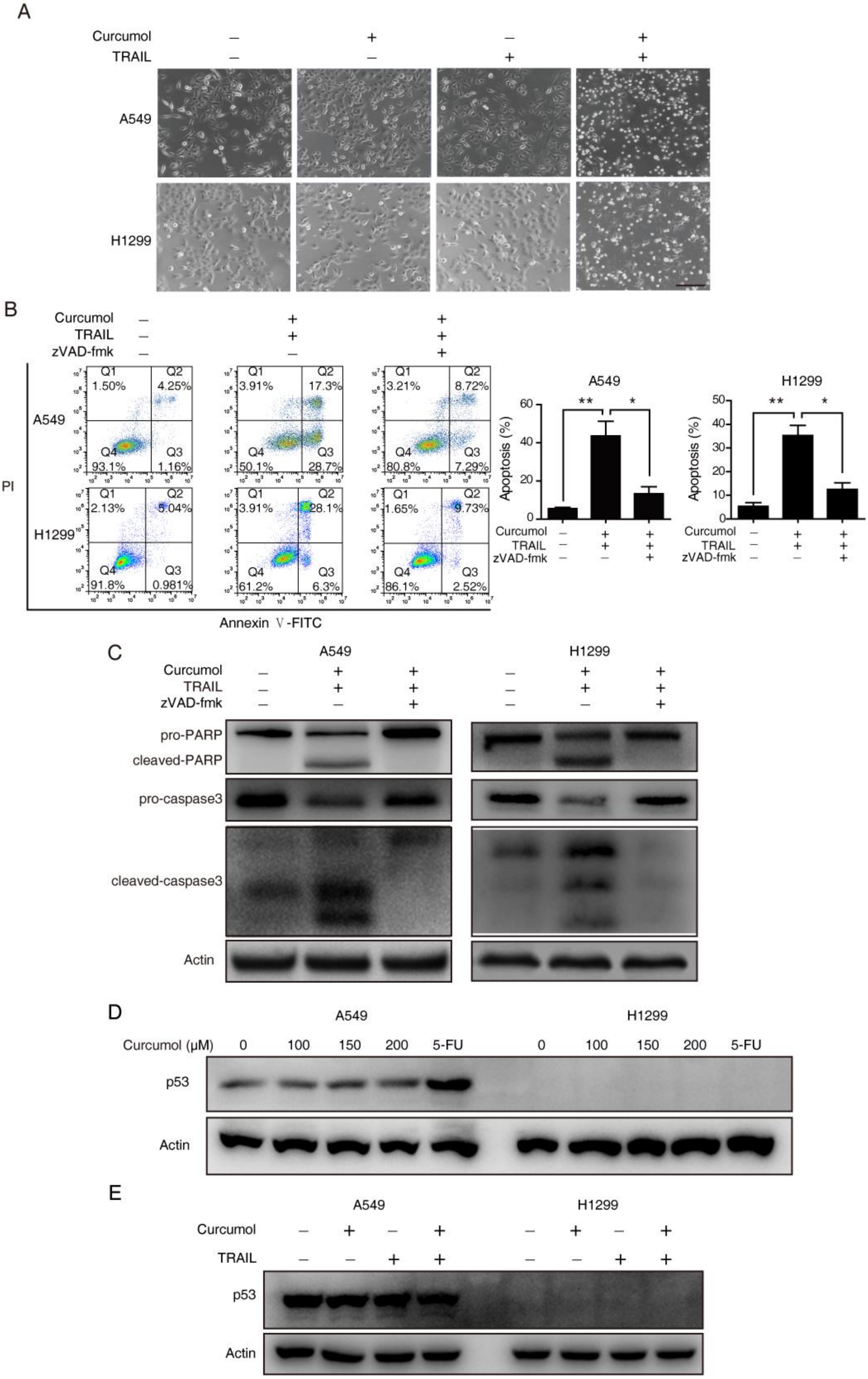
A
Curcumol
TRAIL
A549
H1299
B
Curcumol
TRAIL
zVAD-fmk
PI
Annexin Ⅴ-FITC
Q1 1.50%
Q2 4.25%
Q4 93.1%
Q3 1.16%
Q1 3.91%
Q2 17.3%
Q4 50.1%
Q3 28.7%
Q1 3.21%
Q2 8.72%
Q4 80.8%
Q3 7.29%
Q1 2.13%
Q2 5.04%
Q4 91.8%
Q3 0.981%
Q1 3.91%
Q2 28.1%
Q4 61.2%
Q3 6.3%
Q1 1.65%
Q2 9.73%
Q4 86.1%
Q3 2.52%
Apoptosis (%)
C
pro-PARP
cleaved-PARP
pro-caspase3
cleaved-caspase3
Actin
D
Curcumol (μM) 0 100 150 200 5-FU
p53
E

Figure S2. Curcumol enhances TRAIL-induced apoptosis. (A) A549 and H1299 cells were treated with curcumol (150 μM) and/or TRAIL (25 ng/mL for A549, 50 ng/mL for H1299) as indicated for 24 h, and then cell morphology was examined under a phase contrast microscope. Scale bar, 100μm. (B, C) NSCLC cells were pretreated with or without 10 μM pan-caspase inhibitor Z-VAD-FMK for 1 h, and then treated with curcumol and TRAIL as indicated for 24 h, respectively, the apoptotic cells were stained with Annexin V/PI and analyzed by flow cytometry (B) and the expression of apoptosis-related proteins were detected by immunoblotting (C). (D) A549 and H1299 cells were treated with curcumol at increasing concentrations (0, 100, 150, and 200 μM) and with positive control 5-FU (20 μM) for 24 h. The p53 expression level was determined by Western blotting assay and actin was used as loading control. (E) A549 and H1299 cells treated with indicating curcumol (150 μM) and / or TRAIL (25 ng/mL for A549, 50 ng/mL for H1299) for 24 h and the protein level of p53 was detected by Western blotting. Bars, SEM; N = 3; **P* < 0.05, ***P* < 0.01.

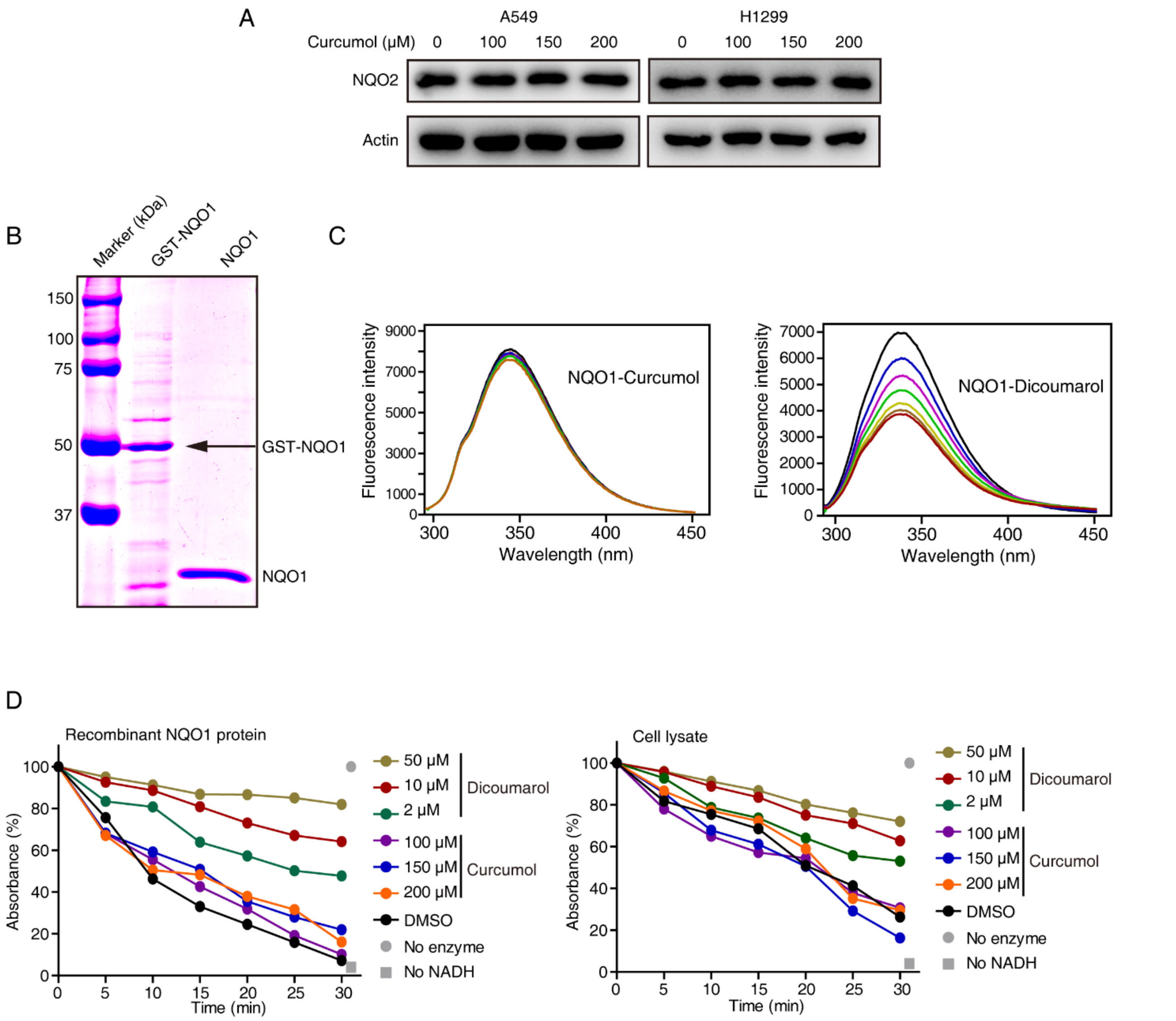

Figure S3. Role of curcumol on NQO2 expression and the effect of curcumol on NQO1 binding. (A) A549 and H1299 cells were incubated with indicated concentrations of curcumol, the NQO2 expression was determined by immunoblotting. (B) The recombinant NQO1 protein generated by pGEX-4T-1 plasmid was purified by GST-tag, the uncut GST-NQO1 and the cut NQO1 were displayed by coomassie brilliant blue staining. (C) The interaction between NQO1 and curcumol or dicoumarol (positive control inhibitor) was determined by fluorescence titration experiments. (D) Enzymatic activity of recombinant NQO1 protein (left panel) and NQO1 protein in cell lysate (right panel) in the presence of curcumol or dicoumarol was determined by measuring the absorbance at 560 nm.

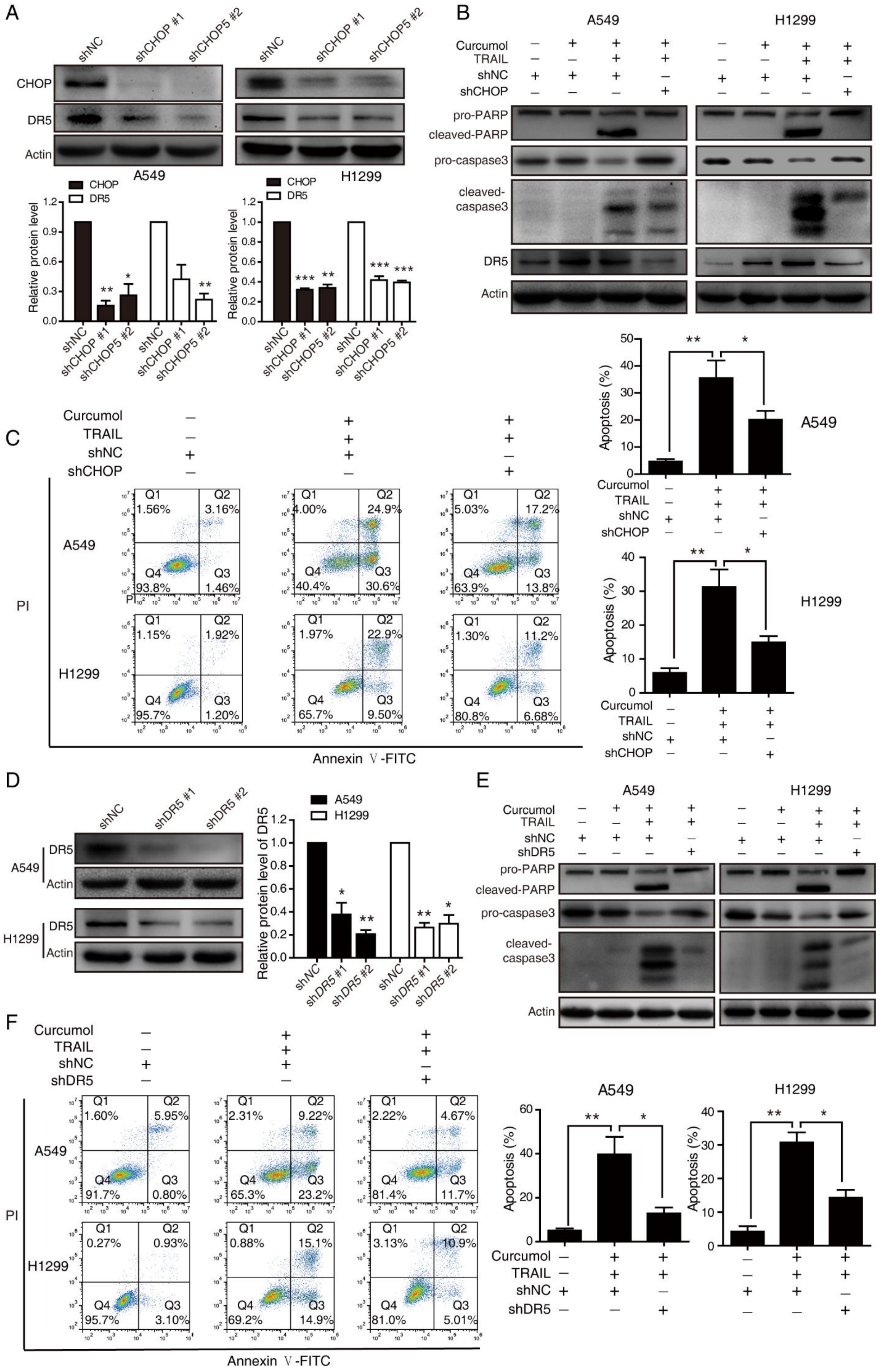
A
shNC
shCHOP #1
shCHOP5 #2
CHOP
DR5
Actin
A549
H1299
CHOP
DR5
Relative protein level
B
Curcumol
TRAIL
shNC
shCHOP
pro-PARP
cleaved-PARP
pro-caspase3
cleaved-caspase3
Actin
Apoptosis (%)
C
PI
Annexin Ⅴ-FITC
D
shDR5 #1
shDR5 #2
Relative protein level of DR5
E
shDR5
F

Figure S4. Role of CHOP and DR5 in curcumol/TRAIL-induced apoptosis. (A) A549 and H1299 cell lines with stably knockdown of DR5 or corresponding scramble, the expression of CHOP and DR5 were analyzed by Western blotting. (B, C) A549 and H1299 with silenced DR5 were treated with curcumol/TRAIL for 24 h, respectively, the expression of apoptosis-related proteins was detected by immunoblotting (B), and the apoptotic cells were analyzed by Annexin V/PI assays (C). (D) Western blot analysis detected the effects of CHOP silencing in indicated cell lines. (E, F) A549 and H1299 with silenced CHOP were treated with curcumol/TRAIL for 24 h, respectively, the expression of apoptosis-related proteins was detected by immunoblotting (E), and the apoptotic cells were analyzed by Annexin V/PI assays (F).

A

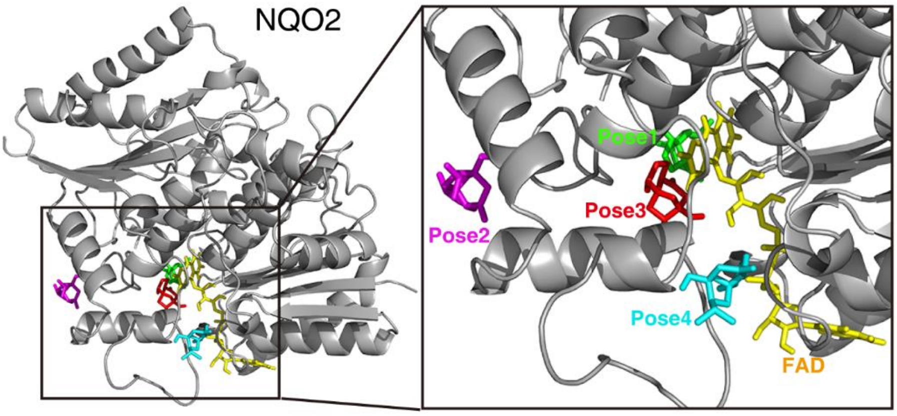

B

Poses	Conformations	Binding Energy (kJ/mol)
Pose1 (Green)	463/500	-7.18
Pose2 (Pink)	12/500	-7.12
Pose3 (Red)	16/500	-6.58
Pose4 (Cyan)	9/500	-5.26

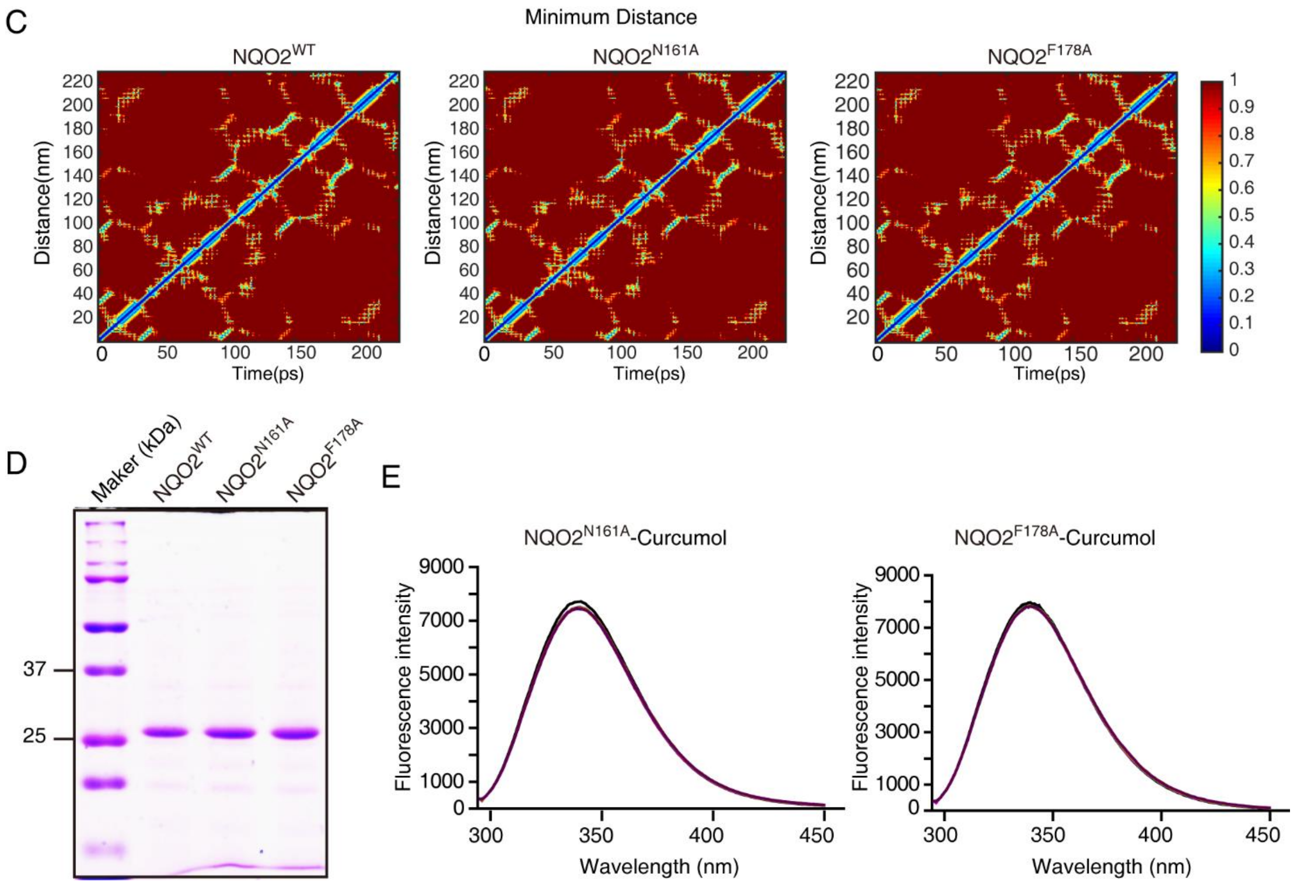

Figure S5. NQO2 mutants in curcumol interaction. (A) Lamarckian genetic algorithm (LGA) search method was applied to establish the best pose of curcumol in NQO2. Global optimization generated 500 randomly initial conformations for curcumol, and formed four conformer clusters with different orientation. The four poses of curcumol on NQO2 with different orientation were

colored, Pose1, Green; Pose2, Pink; Pose3, Red; Pose4, Cyan. The conformation score and binding energy for each pose were listed in (B). (C)Minimum distance matrices for the different mutation states of the NQO2 protein. (D) Coomassie brilliant blue staining analyzed the expression of purified NQO2 and its mutants. (E) Fluorescence titration experiments revealed no interaction between curcumol and NQO2 mutants with either Asn161 or Phe178 residue mutated.

Supplementary Tables

Table S1. Candidate compounds identified from the drug screening. Cell viability was assessed in A549 cells treated with each selected compound combining with or without TRAIL. See separate pdf file.

Table S2. Protein identifications and quantifications based on CETSA-Proteomics. See separate pdf file.

Table S3. Correlation between NQO2 expression and clinicopathological parameters in 94 cases of NSCLC cancer.

Characteristics	n	Low NQO2	High NQO2	P value
Age				0.726
<60	43	14 (32.56%)	29 (67.44%)	
≥60	51	17 (33.33%)	34 (66.67%)	
Gender				0.265
Male	53	20 (37.74%)	33 (62.26%)	
Female	41	11 (26.83%)	30 (73.17%)	
**T-stage**				**0.037**
T1/2	62	23 (37.10%)	39 (62.90%)	
T3/4	23	3 (13.04%)	20 (86.96%)	
**Nodal status**				0.668
N0	34	11 (32.35%)	23 (67.65%)	
N1	50	14 (28.00%)	36 (72.00%)	

Table S4. Sequence of primers for qPCR, vector construction and oligonucleotides. See separate pdf file.

Table S1. Candidate compounds identified from the drug screen.
Cell viability was assessed in A549 cells treated with each selected compound combining with or without TRAIL.

NO.	Drug name	Cell viability of monotherapy				Cell viability of combination therapy				Toxicity of combination therapy			
		Rep.1	Rep.2	Rep.3	Mean	Rep.1	Rep.2	Rep.3	Mean	Rep.1	Rep.2	Rep.3	Mean
	DMSO	1.02	0.99	0.98	1	1.02	0.99	0.98	1	-0.02	0.01	0.02	0
1	Curcumol	0.96	0.95	0.98	0.96	0.29	0.26	0.2	0.25	0.71	0.74	0.8	0.75
2	Neosperidin dihydrochalcone	0.91	0.93	0.98	0.94	0.82	0.94	0.88	0.88	0.18	0.06	0.12	0.12
3	Echinatin	0.91	0.97	0.89	0.92	0.85	0.87	0.77	0.83	0.15	0.13	0.23	0.17
4	Phlorizin dihydrate	0.97	0.87	0.83	0.89	0.78	0.69	0.69	0.72	0.22	0.31	0.31	0.28
5	Isovanillin	0.75	0.81	0.96	0.84	0.83	0.74	0.68	0.75	0.17	0.26	0.32	0.25
6	Mandelic acid	0.82	0.74	0.9	0.82	0.58	0.69	0.56	0.61	0.42	0.31	0.44	0.39
7	2-(4-Hydroxyphenyl)ethanol	0.8	0.64	0.96	0.8	0.78	0.84	0.81	0.81	0.22	0.16	0.19	0.19
8	Panaxatriol	0.6	0.96	0.77	0.78	0.77	0.59	0.65	0.67	0.23	0.41	0.35	0.33
9	Rebaudioside A	0.77	0.74	0.8	0.77	0.64	0.66	0.86	0.72	0.36	0.34	0.14	0.28
10	Coixol	0.77	0.61	0.93	0.77	0.43	0.56	0.6	0.53	0.57	0.44	0.4	0.47
11	L-Carnitine inner salt	0.75	0.71	0.79	0.75	0.67	0.55	0.61	0.61	0.33	0.45	0.39	0.39
12	2-Methoxycinnamic acid	0.74	0.58	0.9	0.74	0.33	0.45	0.42	0.4	0.67	0.55	0.58	0.6
13	3,4,5-Trimethoxycinnamic acid	0.73	0.58	0.88	0.73	0.51	0.57	0.48	0.52	0.49	0.43	0.52	0.48
14	Macranthoidin B	0.73	0.65	0.81	0.73	0.56	0.47	0.5	0.51	0.44	0.53	0.5	0.49
15	Synephrine	0.72	0.55	0.89	0.72	0.61	0.58	0.55	0.58	0.39	0.42	0.45	0.42
16	(-)-Bilobalide	0.72	0.71	0.73	0.72	0.56	0.43	0.57	0.52	0.44	0.57	0.43	0.48
17	Mogroside V	0.71	0.62	0.8	0.71	0.58	0.63	0.47	0.56	0.42	0.37	0.53	0.44
18	Orcinol	0.7	0.54	0.86	0.7	0.52	0.58	0.64	0.58	0.48	0.42	0.36	0.42
19	Ginkgolide C	0.7	0.64	0.76	0.7	0.44	0.45	0.34	0.41	0.56	0.55	0.66	0.59
20	Synephrine HCl	0.63	0.55	0.86	0.68	0.49	0.57	0.62	0.56	0.51	0.43	0.38	0.44
21	Glycyrrhizic acid ammonium salt	0.67	0.57	0.72	0.66	0.57	0.57	0.45	0.53	0.43	0.43	0.55	0.47
22	Nardosinone	0.64	0.61	0.69	0.65	0.62	0.76	0.6	0.66	0.38	0.24	0.4	0.34
23	20R-Ginsenoside Rg2	0.67	0.54	0.72	0.65	0.5	0.37	0.48	0.45	0.5	0.63	0.52	0.55
24	Jujuboside A	0.67	0.55	0.72	0.64	0.61	0.44	0.51	0.52	0.39	0.56	0.49	0.48
25	Pseudoginsenoside F11	0.67	0.55	0.72	0.64	0.5	0.32	0.44	0.42	0.5	0.68	0.56	0.58
26	Helicid	0.64	0.59	0.69	0.64	0.38	0.56	0.5	0.48	0.62	0.44	0.5	0.52

27	Shionone	0.64	0.59	0.69	0.64	0.33	0.53	0.43	0.43	0.67	0.47	0.57	0.57
28	Angelic acid	0.69	0.53	0.69	0.64	0.44	0.32	0.29	0.35	0.56	0.68	0.71	0.65
29	Vitexin-2"-O-rhamnoside	0.67	0.66	0.57	0.63	0.56	0.64	0.66	0.62	0.44	0.36	0.34	0.38
30	Rebaudioside C	0.67	0.51	0.72	0.63	0.72	0.84	0.78	0.78	0.28	0.16	0.22	0.22
31	Oxysophocarpine	0.69	0.61	0.59	0.63	0.58	0.53	0.63	0.58	0.42	0.47	0.37	0.42
32	Sakakin	0.67	0.5	0.72	0.63	0.33	0.55	0.47	0.45	0.67	0.45	0.53	0.55
33	7-hydroxy-4-methyl-8-nitrocoumarin	0.69	0.6	0.59	0.63	0.51	0.41	0.46	0.46	0.49	0.59	0.54	0.54
34	Glucosylvitexin	0.68	0.61	0.58	0.63	0.22	0.46	0.22	0.3	0.78	0.54	0.78	0.7
35	Columbianadin	0.64	0.52	0.69	0.62	0.62	0.46	0.51	0.53	0.38	0.54	0.49	0.47
36	Sophoricoside	0.65	0.64	0.55	0.61	0.52	0.43	0.49	0.48	0.48	0.57	0.51	0.52
37	Methyl hesperidin	0.66	0.47	0.71	0.61	0.42	0.54	0.48	0.48	0.58	0.46	0.52	0.52
38	Ethyl coumarin-3-carboxylate	0.66	0.46	0.71	0.61	0.27	0.48	0.21	0.32	0.73	0.52	0.79	0.68
39	Hordenine	0.6	0.54	0.65	0.6	0.45	0.56	0.58	0.53	0.55	0.44	0.42	0.47
40	Methyl syringate	0.62	0.47	0.67	0.59	0.7	0.64	0.52	0.62	0.3	0.36	0.48	0.38
41	Macranthoidin A	0.63	0.45	0.68	0.59	0.38	0.31	0.42	0.37	0.62	0.69	0.58	0.63
42	Sinapine thiocyanate	0.62	0.46	0.67	0.58	0.2	0.18	0.13	0.17	0.8	0.82	0.87	0.83
43	Ethyl Vanillate	0.6	0.45	0.65	0.57	0.39	0.47	0.43	0.43	0.61	0.53	0.57	0.57
44	Perillartine	0.58	0.49	0.48	0.52	0.42	0.6	0.39	0.47	0.58	0.4	0.61	0.53
45	4-(4-Hydroxyphenyl)-2-butanone	0.56	0.38	0.46	0.47	0.39	0.55	0.38	0.44	0.61	0.45	0.62	0.56
46	Nodakenin	0.56	0.4	0.46	0.47	0.4	0.43	0.46	0.43	0.6	0.57	0.54	0.57
47	trans-4-phenylbut-3-en-2-one	0.43	0.28	0.33	0.34	0.24	0.48	0.27	0.33	0.76	0.52	0.73	0.67
48	Isochlorogenic acid C	0.43	0.23	0.33	0.33	0.58	0.5	0.6	0.56	0.42	0.5	0.4	0.44
49	Elemicin	0.42	0.27	0.32	0.33	0.13	0.26	0.24	0.21	0.87	0.74	0.76	0.79
50	Liensinine perchlorate	0.23	0.13	0.37	0.24	0.38	0.29	0.11	0.26	0.62	0.71	0.89	0.74
51	Daurisoline	0.28	0.22	0.11	0.2	0.35	0.47	0.35	0.39	0.65	0.53	0.65	0.61
52	Xanthoxylin	0.28	0.11	0.18	0.19	0.22	0.12	0.26	0.2	0.78	0.88	0.74	0.8

Table S2. Protein identifications and quantifications based on CETSA-Proteomics.

NO.	Accession	Gene name	MW(kDa)	Abundance Ratio:Light (Curcumol)/ Heavy (DMSO)				Ratio change			
				40 ℃	64 ℃	67 ℃	70 ℃	40 ℃/40 ℃	64 ℃/40 ℃	67 ℃/40 ℃	70 ℃/40 ℃
1	P50453	SERPINB9	42.40	0.56	0.81	0.57	100.00	1.00	1.46	1.01	179.21
2	P30043	BLVRB	22.12	0.73	0.67	0.34	100.00	1.00	0.93	0.47	137.74
3	Q9UBQ7	GRHPR	35.67	0.81	0.80	0.84	100.00	1.00	0.99	1.04	123.76
4	O75369	FLNB	278.16	0.85	0.83	0.94	100.00	1.00	0.98	1.10	117.79
5	Q01518	CAP1	51.90	0.89	0.99		100.00	1.00	1.11	0.00	111.86
6	P49458	SRP9	10.11	0.93	1.10	1.07	100.00	1.00	1.19	1.16	108.11
7	P35270	SPR	28.05	0.94	0.93	0.63	100.00	1.00	1.00	0.67	106.95
8	Q9UNZ2	NSFL1C	40.57	1.04	1.10	2.09	100.00	1.00	1.06	2.02	96.34
9	Q14244	MAP7	84.05	1.04	0.70	1.22	100.00	1.00	0.68	1.18	96.15
10	P50395	GDI2	50.66	1.09	1.09	1.18	100.00	1.00	1.00	1.08	91.58
11	P09661	SNRPA1	28.42	1.10	1.14	1.87	100.00	1.00	1.04	1.70	90.83
12	P13645	KRT10	58.83	5.52	11.35	100.00	100.00	1.00	2.06	18.11	18.11
13	P60903	S100A10	11.20	1.07	0.90	100.00	6.71	1.00	0.84	93.72	6.29
14	Q13813	SPTAN1	284.54	1.08	1.03	1.49	5.83	1.00	0.96	1.38	5.41
15	Q02818	NUCB1	53.88	0.11	0.67	0.40	0.39	1.00	6.17	3.65	3.59
16	P15311	EZR	69.41	0.86	0.90	2.19	2.85	1.00	1.05	2.55	3.31
17	Q9Y5S9	RBM8A	19.89	0.66	1.65		2.02	1.00	2.49	0.00	3.05
18	P28482	MAPK1	41.39	0.88	0.98	0.99	2.62	1.00	1.11	1.13	2.99
19	P37235	HPCAL1	22.31	0.78	1.44	1.38	2.28	1.00	1.86	1.79	2.94
20	P27635	RPL10	24.60	0.96	1.07	1.12	2.82	1.00	1.11	1.17	2.94
21	Q9Y6N5	SQRDL	49.96	0.82	0.60	0.68	2.39	1.00	0.72	0.83	2.90
22	P16083	NQO2	25.92	0.49	1.06	1.33	1.34	1.00	2.16	2.70	2.74
23	Q9P1F3	ABRACL	9.06	0.49	0.81	0.85	1.33	1.00	1.65	1.74	2.72
24	O00244	ATOX1	7.40	0.96	0.20	0.29	2.39	1.00	0.20	0.30	2.49
25	O15305	PMM2	28.08	1.16	1.37	2.47	2.74	1.00	1.18	2.14	2.37
26	P63241	EIF5A	16.83	1.39	1.37	2.68	3.22	1.00	0.98	1.93	2.32
27	P19338	NCL	76.61	1.28	1.22	1.99	2.90	1.00	0.96	1.56	2.28
28	Q14232	EIF2B1	33.71	1.08	1.64	1.14	2.38	1.00	1.52	1.06	2.21

29	P38919	EIF4A3	46.87	1.20	2.94		2.64	1.00	2.45	0.00	2.20
30	P05787	KRT8	53.70	0.73	0.68	1.14	1.58	1.00	0.94	1.57	2.18
31	P05783	KRT18	48.06	0.79	0.76	2.84	1.70	1.00	0.96	3.60	2.15
32	P52209	PGD	53.14	0.82	0.83	0.84	1.75	1.00	1.01	1.02	2.13
33	Q9C0C2	TNKS1BP1	181.79	0.35	0.63	0.60	0.73	1.00	1.81	1.72	2.10
34	P61978	HNRNPK	50.98	1.03	0.98	0.71	2.11	1.00	0.96	0.69	2.06
35	Q9UI30	TRMT112	14.20	1.01	1.16	1.36	1.99	1.00	1.15	1.35	1.98
36	P07900	HSP90AA1	84.66	1.20	1.01	3.04	2.34	1.00	0.84	2.52	1.94
37	O43670	ZNF207	50.75	0.73	1.29	1.58	1.41	1.00	1.77	2.16	1.93
38	Q15102	PAFAH1B3	25.73	1.21	1.09	1.59	2.33	1.00	0.90	1.31	1.92
39	Q01469	FABP5	15.16	1.52	1.53	1.59	2.91	1.00	1.01	1.05	1.92
40	Q9UIJ7	AK3	25.57	0.66	0.89	1.09	1.27	1.00	1.34	1.64	1.91
41	Q92688	ANP32B	28.79	1.35	1.24	1.27	2.46	1.00	0.92	0.94	1.82
42	P31947	SFN	27.77	1.38	1.38	1.50	2.50	1.00	1.00	1.09	1.82
43	Q16851	UGP2	56.94	0.83	0.80	0.86	1.50	1.00	0.97	1.03	1.81
44	O00273	DFFA	36.52	0.71	4.60	0.98	1.27	1.00	6.53	1.39	1.81
45	O60832	DKC1	57.67	0.88	1.69	1.15	1.59	1.00	1.91	1.30	1.81
46	P09497	CLTB	25.19	0.64	0.87	0.92	1.14	1.00	1.35	1.44	1.78
47	P23284	PPIB	23.74	1.04	1.06	1.76	1.85	1.00	1.02	1.70	1.78
48	P10809	HSPD1	61.05	1.18	1.18	1.14	2.02	1.00	1.00	0.96	1.72
49	P36405	ARL3	20.46	1.11	0.30	1.24	1.88	1.00	0.27	1.12	1.69
50	O75531	BANF1	10.06	0.81	0.83	0.94	1.36	1.00	1.02	1.16	1.68
51	P15880	RPS2	31.32	1.15	1.14	1.23	1.89	1.00	0.99	1.08	1.65
52	P30838	ALDH3A1	50.39	0.96	0.97	1.10	1.58	1.00	1.00	1.15	1.65
53	Q99584	S100A13	11.47	0.80	0.98	0.86	1.32	1.00	1.22	1.08	1.64
54	P41567	EIF1	12.73	1.24	1.31	1.59	2.01	1.00	1.06	1.28	1.62
55	P62995	TRA2B	33.67	1.17	1.45	1.37	1.90	1.00	1.24	1.17	1.62
56	Q99729	HNRNPAB	36.22	1.10	1.37	1.72	1.77	1.00	1.25	1.57	1.62
57	P08670	VIM	53.65	1.82	1.10	1.62	2.94	1.00	0.61	0.89	1.62
58	P43490	NAMPT	55.52	1.46	1.45	1.69	2.35	1.00	1.00	1.16	1.61
59	P27797	CALR	48.14	1.07	1.06	1.54	1.72	1.00	1.00	1.44	1.61
60	P30101	PDIA3	56.78	1.02	1.05	2.32	1.64	1.00	1.03	2.28	1.61

61	P13667	PDIA4	72.93	1.12	0.94	0.91	1.80	1.00	0.84	0.81	1.61
62	Q8NDH3	NPEPL1	55.86	0.45	0.62	0.70	0.73	1.00	1.35	1.53	1.60
63	Q8N357	SLC35F6	40.21	0.64	0.74	1.27	1.01	1.00	1.17	2.00	1.59
64	P61158	ACTR3	47.37	0.62	0.83	0.72	0.98	1.00	1.35	1.17	1.58
65	P60510	PPP4C	35.08	1.07	1.11	0.38	1.67	1.00	1.04	0.36	1.56
66	Q9H6Z4	RANBP3	60.21	0.98	1.65	1.18	1.52	1.00	1.68	1.20	1.55
67	P62244	RPS15A	14.84	1.20	1.15	1.74	1.85	1.00	0.96	1.45	1.53
68	P21333	FLNA	280.74	0.80	0.76	1.29	1.22	1.00	0.96	1.62	1.53
69	P62854	RPS26	13.02	1.22	1.10	1.20	1.88	1.00	0.90	0.98	1.53
70	P09651	HNRNPA1	38.75	1.19	0.94	1.35	1.82	1.00	0.80	1.14	1.53
71	Q8IZ83	ALDH16A1	85.13	0.67	1.41	3.01	1.02	1.00	2.11	4.49	1.52
72	P31937	HIBADH	35.33	0.49	0.77	0.69	0.75	1.00	1.57	1.40	1.52
73	P09972	ALDOC	39.46	0.73	1.13	0.98	1.10	1.00	1.55	1.35	1.51
74	O75348	ATP6V1G1	13.76	1.03	1.03	1.15	1.54	1.00	1.00	1.11	1.49
75	Q99598	TSNAX	33.11	0.36	100.00	1.17	0.54	1.00	276.24	3.24	1.49
76	Q9BY32	ITPA	21.45	0.45	0.99	0.81	0.67	1.00	2.21	1.81	1.49
77	Q9Y3C1	NOP16	21.19	1.28	1.32	0.94	1.90	1.00	1.03	0.73	1.48
78	P05386	RPLP1	11.51	0.87	1.33	1.19	1.29	1.00	1.52	1.36	1.47
79	P08238	HSP90AB1	83.26	1.14	0.99	100.00	1.67	1.00	0.87	88.11	1.47
80	Q01581	HMGCS1	57.29	1.79	1.71	2.22	2.63	1.00	0.96	1.24	1.47
81	Q00839	HNRNPU	90.58	1.28	0.76	1.46	1.87	1.00	0.59	1.14	1.46
82	P83731	RPL24	17.78	1.14	1.18	1.26	1.66	1.00	1.03	1.11	1.45
83	P05387	RPLP2	11.66	1.07	1.28	1.33	1.55	1.00	1.20	1.24	1.45
84	O14745	SLC9A3R1	38.87	1.23	1.45	1.55	1.78	1.00	1.18	1.26	1.45
85	Q9HA64	FN3KRP	34.41	0.71	1.11	0.88	1.03	1.00	1.56	1.24	1.45
86	P31939	ATIC	64.62	1.08	1.04	1.12	1.56	1.00	0.96	1.04	1.45
87	P62701	RPS4X	29.60	1.20	1.25	1.16	1.74	1.00	1.04	0.96	1.45
88	Q9NVA2	SEPT11	49.40	0.66	0.86	0.90	0.95	1.00	1.31	1.37	1.45
89	P14625	HSP90B1	92.47	1.07	0.92	0.93	1.54	1.00	0.86	0.87	1.44
90	Q14019	COTL1	15.94	0.94	1.01	0.78	1.34	1.00	1.08	0.83	1.44
91	P04083	ANXA1	38.71	1.03	1.04	1.73	1.48	1.00	1.01	1.68	1.44
92	Q00169	PITPNA	31.81	0.87	0.99	1.00	1.25	1.00	1.14	1.15	1.44

93	Q13404	UBE2V1	16.49	1.06	1.08	1.19	1.52	1.00	1.02	1.13	1.44
94	P07355	ANXA2	38.60	0.97	0.97	1.42	1.39	1.00	1.01	1.46	1.43
95	P14314	PRKCSH	59.42	1.08	1.00	1.07	1.54	1.00	0.93	0.99	1.43
96	Q06830	PRDX1	22.11	0.95	0.95	1.02	1.35	1.00	0.99	1.07	1.42
97	O94760	DDAH1	31.12	0.59	3.59	0.79	0.84	1.00	6.09	1.34	1.42
98	P50213	IDH3A	39.59	1.09	1.19	1.06	1.54	1.00	1.09	0.98	1.41
99	P60983	GMFB	16.71	0.76	1.21	1.06	1.07	1.00	1.59	1.40	1.41
100	P62753	RPS6	28.68	1.11	1.22	1.17	1.56	1.00	1.10	1.06	1.41
101	P11908	PRPS2	34.77	1.18	1.60	0.42	1.65	1.00	1.35	0.36	1.39
102	Q86VP6	CAND1	136.37	1.10	1.04	1.08	1.53	1.00	0.95	0.98	1.39
103	P60953	CDC42	21.26	0.79	1.02	0.65	1.10	1.00	1.29	0.83	1.39
104	P07741	APRT	19.61	1.12	0.99	1.28	1.55	1.00	0.89	1.14	1.38
105	Q14978	NOLC1	73.60	1.21	1.15	1.53	1.67	1.00	0.95	1.27	1.38
106	Q9H0D6	XRN2	108.58	0.85	1.42	1.26	1.18	1.00	1.66	1.49	1.38
107	P38606	ATP6V1A	68.30	0.93	0.88	1.09	1.28	1.00	0.95	1.17	1.38
108	P20962	PTMS	11.53	0.71	0.76	0.94	0.98	1.00	1.07	1.31	1.38
109	Q8NCW5	NAXE	31.67	0.90	1.22	0.99	1.23	1.00	1.36	1.10	1.37
110	Q15084	PDIA6	48.12	1.19	1.19	1.12	1.62	1.00	1.00	0.95	1.37
111	P22626	HNRNPA2B1	37.43	1.26	0.95	1.61	1.72	1.00	0.75	1.28	1.37
112	O95994	AGR2	19.98	0.85	0.73	0.87	1.15	1.00	0.87	1.03	1.36
113	Q9NQP4	PFDN4	15.31	1.05	0.95	2.21	1.43	1.00	0.90	2.10	1.36
114	P12004	PCNA	28.77	1.47	1.70	1.82	2.00	1.00	1.15	1.24	1.36
115	O94992	HEXIM1	40.62	1.05	1.63	1.49	1.43	1.00	1.55	1.42	1.36
116	Q92973	TNPO1	102.35	1.27	1.60	2.49	1.72	1.00	1.26	1.97	1.36
117	Q9NQC3	RTN4	129.93	0.83	1.01	0.98	1.12	1.00	1.23	1.18	1.35
118	O95336	PGLS	27.55	0.66	0.77	0.80	0.89	1.00	1.16	1.22	1.35
119	P62266	RPS23	15.81	1.22	1.29	1.29	1.64	1.00	1.06	1.06	1.35
120	P51149	RAB7A	23.49	0.74	0.99	0.96	0.99	1.00	1.34	1.29	1.34
121	O95817	BAG3	61.59	0.97	0.92	1.28	1.29	1.00	0.94	1.32	1.33
122	O00154	ACOT7	41.80	0.89	1.06	1.12	1.17	1.00	1.19	1.27	1.33
123	Q16204	CCDC6	53.29	1.26	0.92	0.98	1.67	1.00	0.73	0.78	1.32
124	O95810	SDPR	47.17	0.54	0.55	0.77	0.71	1.00	1.01	1.43	1.32

125	Q99714	HSD17B10	26.92	1.08	1.04	1.03	1.42	1.00	0.96	0.95	1.31
126	Q96EM0	L3HYPDH	38.14	0.74	0.77	0.90	0.97	1.00	1.04	1.23	1.31
127	O43633	CHMP2A	25.10	0.64	0.75	0.99	0.83	1.00	1.17	1.55	1.30
128	P16401	HIST1H1B	22.58	1.10	1.17	1.10	1.43	1.00	1.07	1.00	1.30
129	P49368	CCT3	60.53	1.24	1.23	1.20	1.62	1.00	0.99	0.96	1.30
130	P98179	RBM3	17.17	0.62	1.06	0.80	0.81	1.00	1.70	1.29	1.30
131	P62841	RPS15	17.04	1.21	1.34	1.53	1.57	1.00	1.11	1.26	1.30
132	P55769	SNU13	14.17	0.85	0.85	0.96	1.10	1.00	1.00	1.12	1.30
133	P07305	H1F0	20.86	0.58	0.67	0.57	0.75	1.00	1.15	0.99	1.29
134	P17931	LGALS3	26.15	0.72	0.79	0.96	0.93	1.00	1.10	1.32	1.28
135	P30520	ADSS	50.10	0.93	1.07	1.09	1.20	1.00	1.14	1.16	1.28
136	P53999	SUB1	14.40	0.96	0.94	1.19	1.23	1.00	0.98	1.24	1.28
137	P39687	ANP32A	28.59	1.08	1.15	1.23	1.38	1.00	1.07	1.14	1.28
138	P62263	RPS14	16.27	1.21	1.25	1.03	1.54	1.00	1.03	0.86	1.27
139	Q8N8S7	ENAH	66.51	0.79	0.94	0.81	1.00	1.00	1.19	1.03	1.27
140	P60891	PRPS1	34.83	1.30	1.26	1.33	1.65	1.00	0.97	1.03	1.27
141	P47756	CAPZB	31.35	0.87	1.26	1.17	1.10	1.00	1.45	1.35	1.27
142	P55786	NPEPPS	103.28	0.78	0.84	0.83	0.99	1.00	1.08	1.06	1.27
143	Q15293	RCN1	38.89	0.77	0.91	0.93	0.98	1.00	1.17	1.20	1.27
144	P04156	PRNP	27.66	0.54	0.72	0.84	0.68	1.00	1.35	1.57	1.27
145	Q8IV08	PLD3	54.70	0.67	0.80	0.97	0.85	1.00	1.19	1.45	1.27
146	P25705	ATP5A1	59.75	0.84	1.00	1.01	1.06	1.00	1.18	1.19	1.26
147	P62979	RPS27A	17.96	1.08	1.18	1.25	1.36	1.00	1.10	1.16	1.26
148	P08758	ANXA5	35.94	0.79	0.71	0.95	0.99	1.00	0.90	1.20	1.26
149	P23381	WARS	53.16	1.02	1.29	1.15	1.28	1.00	1.26	1.12	1.26
150	P78371	CCT2	57.49	1.50	1.50	1.61	1.89	1.00	1.00	1.07	1.26
151	P17987	TCP1	60.34	1.25	1.25	1.35	1.56	1.00	1.00	1.09	1.25
152	P48637	GSS	52.38	0.73	0.78	0.89	0.91	1.00	1.07	1.23	1.24
153	P26038	MSN	67.82	0.88	0.86	1.10	1.10	1.00	0.98	1.25	1.24
154	Q12797	ASPH	85.86	1.70	1.20	1.52	2.12	1.00	0.70	0.89	1.24
155	P17812	CTPS1	66.69	1.06	1.11	1.17	1.32	1.00	1.04	1.10	1.24
156	P13929	ENO3	46.99	0.76	0.81	0.91	0.95	1.00	1.05	1.19	1.24

157	Q99471	PFDN5	17.33	0.96	1.63	1.24	1.19	1.00	1.70	1.29	1.24
158	O75533	SF3B1	145.83	1.08	0.75	1.11	1.34	1.00	0.69	1.03	1.24
159	Q9BRA2	TXNDC17	13.94	1.16	1.15	1.20	1.43	1.00	0.99	1.03	1.23
160	Q9HB07	C12orf10	42.45	0.93	1.14	1.01	1.14	1.00	1.22	1.08	1.23
161	P15559	NQO1	30.87	0.96	0.94	0.84	1.18	1.00	0.98	0.88	1.23
162	P28074	PSMB5	28.48	1.22	1.54	1.41	1.50	1.00	1.25	1.15	1.22
163	P13473	LAMP2	44.96	0.57	0.61	0.52	0.69	1.00	1.08	0.91	1.22
164	P84085	ARF5	20.53	0.83	0.97	0.54	1.01	1.00	1.17	0.66	1.22
165	Q13616	CUL1	89.68	1.08	1.13	1.73	1.32	1.00	1.05	1.60	1.22
166	P16403	HIST1H1C	21.36	0.91	1.09	1.02	1.11	1.00	1.20	1.12	1.22
167	P36578	RPL4	47.70	1.19	1.22	1.25	1.45	1.00	1.03	1.05	1.22
168	P32320	CDA	16.18	0.50	0.52	0.50	0.60	1.00	1.05	1.00	1.21
169	P35237	SERPINB6	42.62	0.83	0.85	0.86	1.00	1.00	1.03	1.04	1.21
170	Q9UMX0	UBQLN1	62.52	1.22	1.33	1.18	1.47	1.00	1.09	0.97	1.21
171	P61586	RHOA	21.77	1.01	1.10	1.27	1.22	1.00	1.09	1.26	1.21
172	P61916	NPC2	16.57	0.27	0.29	0.35	0.33	1.00	1.06	1.27	1.21
173	P16949	STMN1	17.30	1.02	1.12	1.17	1.23	1.00	1.10	1.15	1.20
174	Q92890	UFD1L	34.50	1.40	1.29	1.48	1.69	1.00	0.92	1.06	1.20
175	Q9Y281	CFL2	18.74	0.99	1.01	0.79	1.19	1.00	1.02	0.80	1.20
176	Q6NZI2	PTRF	43.48	0.62	0.69	0.78	0.74	1.00	1.12	1.26	1.20
177	P23193	TCEA1	33.97	1.63	1.63	1.70	1.95	1.00	1.00	1.04	1.20
178	Q9HD42	CHMP1A	21.70	0.83	1.00	0.95	0.99	1.00	1.20	1.14	1.19
179	P09936	UCHL1	24.82	0.85	0.87	1.03	1.02	1.00	1.02	1.20	1.19
180	P13798	APEH	81.22	0.93	0.86	1.07	1.11	1.00	0.92	1.14	1.19
181	P36871	PGM1	61.45	1.01	1.06	1.19	1.21	1.00	1.05	1.18	1.19
182	P63104	YWHAZ	27.74	0.98	1.01	1.03	1.16	1.00	1.04	1.05	1.19
183	P50991	CCT4	57.92	1.29	1.34	1.39	1.53	1.00	1.04	1.08	1.19
184	P00441	SOD1	15.94	0.98	1.01	0.58	1.16	1.00	1.03	0.60	1.19
185	P68032	ACTC1	42.02	0.78	1.04	0.93	0.93	1.00	1.33	1.19	1.19
186	P61224	RAP1B	20.82	0.99	0.90	0.93	1.17	1.00	0.91	0.94	1.18
187	Q9NRX4	PHPT1	13.83	0.97	0.94	1.03	1.15	1.00	0.97	1.06	1.18
188	P62891	RPL39	6.41	1.14	1.42	1.22	1.34	1.00	1.25	1.07	1.17

189	P47914	RPL29	17.75	1.09	1.27	1.26	1.28	1.00	1.16	1.15	1.17
190	Q9H1E3	NUCKS1	27.30	0.83	0.87	1.00	0.98	1.00	1.04	1.19	1.17
191	P99999	CYCS	11.75	0.71	0.74	1.19	0.83	1.00	1.04	1.68	1.17
192	P61221	ABCE1	67.31	0.86	0.57	1.01	1.00	1.00	0.66	1.17	1.17
193	P26373	RPL13	24.26	1.30	1.26	1.38	1.51	1.00	0.98	1.07	1.17
194	Q9BQE3	TUBA1C	49.89	1.05	1.11	1.12	1.22	1.00	1.06	1.07	1.16
195	Q14103	HNRNPD	38.43	1.03	1.11	1.13	1.20	1.00	1.08	1.10	1.16
196	P25787	PSMA2	25.90	1.11	1.26	1.24	1.29	1.00	1.14	1.12	1.16
197	P42765	ACAA2	41.92	0.89	0.99	1.14	1.02	1.00	1.12	1.29	1.15
198	P22307	SCP2	58.99	0.65	0.72	0.82	0.75	1.00	1.11	1.26	1.15
199	P68366	TUBA4A	49.92	1.17	1.14	1.16	1.35	1.00	0.98	1.00	1.15
200	P07339	CTSD	44.55	0.50	0.56	0.65	0.57	1.00	1.13	1.30	1.15
201	P50914	RPL14	23.43	1.17	1.29	1.20	1.35	1.00	1.10	1.02	1.15
202	P27816	MAP4	121.00	0.79	0.86	0.87	0.91	1.00	1.08	1.09	1.15
203	P04632	CAPNS1	28.32	0.83	0.80	0.79	0.96	1.00	0.96	0.94	1.15
204	Q07955	SRSF1	27.74	1.11	1.18	1.14	1.27	1.00	1.06	1.02	1.14
205	P42677	RPS27	9.46	1.16	1.16	1.57	1.33	1.00	1.00	1.35	1.14
206	Q9Y230	RUVBL2	51.16	1.10	1.23	1.32	1.26	1.00	1.12	1.19	1.14
207	P30048	PRDX3	27.69	0.85	0.87	0.92	0.97	1.00	1.02	1.08	1.14
208	O43175	PHGDH	56.65	1.15	1.22	1.19	1.31	1.00	1.06	1.03	1.14
209	P40227	CCT6A	58.02	1.26	1.23	1.46	1.43	1.00	0.98	1.16	1.14
210	Q92734	TFG	43.45	0.96	1.10	1.01	1.09	1.00	1.15	1.06	1.14
211	P14854	COX6B1	10.19	0.67	0.67	0.82	0.76	1.00	1.01	1.23	1.14
212	P61758	VBP1	22.66	1.37	1.57	1.32	1.55	1.00	1.15	0.97	1.13
213	P23588	EIF4B	69.15	1.24	1.34	1.54	1.41	1.00	1.08	1.24	1.13
214	P05114	HMGN1	10.66	1.06	1.06	0.83	1.19	1.00	1.01	0.79	1.13
215	P07858	CTSB	37.82	0.41	0.44	0.46	0.47	1.00	1.07	1.12	1.13
216	P62888	RPL30	12.78	1.24	1.27	1.30	1.40	1.00	1.02	1.05	1.13
217	Q9UNS2	COPS3	47.87	1.65	2.02	100.00	1.85	1.00	1.23	60.79	1.13
218	Q96AE4	FUBP1	67.56	1.19	1.26	1.29	1.34	1.00	1.06	1.09	1.13
219	Q01130	SRSF2	25.48	1.11	1.13	1.17	1.25	1.00	1.02	1.05	1.13
220	P39019	RPS19	16.06	1.28	1.25	1.29	1.44	1.00	0.98	1.01	1.12

221	Q6PUV4	CPLX2	15.39	1.93	2.09	1.80	2.17	1.00	1.08	0.93	1.12
222	Q9Y2B0	CNPY2	20.65	1.15	1.01	1.97	1.30	1.00	0.88	1.71	1.12
223	P07437	TUBB	49.67	1.11	1.13	1.21	1.24	1.00	1.02	1.10	1.12
224	P37802	TAGLN2	22.39	0.74	0.70	0.82	0.83	1.00	0.95	1.11	1.12
225	P04792	HSPB1	22.78	0.57	0.55	0.76	0.63	1.00	0.97	1.35	1.12
226	P09429	HMGB1	24.89	1.21	1.27	1.36	1.36	1.00	1.04	1.12	1.12
227	P00813	ADA	40.76	0.88	0.94	0.85	0.99	1.00	1.06	0.96	1.12
228	Q9NQR4	NIT2	30.61	1.09	0.95	0.70	1.21	1.00	0.88	0.64	1.12
229	Q04917	YWHAH	28.22	0.85	0.85	0.87	0.95	1.00	1.01	1.03	1.12
230	Q15637	SF1	68.33	1.23	1.11	1.27	1.38	1.00	0.90	1.03	1.12
231	P68036	UBE2L3	17.86	1.20	1.26	1.10	1.34	1.00	1.05	0.92	1.12
232	P84077	ARF1	20.70	0.91	1.02	1.02	1.02	1.00	1.12	1.12	1.12
233	P68371	TUBB4B	49.83	1.08	1.07	1.09	1.20	1.00	0.99	1.01	1.12
234	Q99832	CCT7	59.37	1.24	1.32	1.38	1.38	1.00	1.06	1.11	1.12
235	Q15029	EFTUD2	109.43	1.20	0.88	1.17	1.34	1.00	0.73	0.97	1.11
236	P02545	LMNA	74.14	1.45	1.25	1.13	1.62	1.00	0.86	0.78	1.11
237	Q9UBQ0	VPS29	20.51	0.88	0.92	0.98	0.98	1.00	1.04	1.11	1.11
238	Q9NR28	DIABLO	27.13	1.22	0.94	1.15	1.35	1.00	0.77	0.94	1.11
239	P78330	PSPH	25.01	0.92	1.10	1.00	1.02	1.00	1.19	1.09	1.11
240	P48643	CCT5	59.67	1.35	1.34	1.46	1.50	1.00	0.99	1.08	1.11
241	P62937	PPIA	18.01	1.00	0.99	1.19	1.11	1.00	0.98	1.18	1.11
242	P80303	NUCB2	50.20	1.07	1.14	1.17	1.19	1.00	1.07	1.09	1.11
243	Q16629	SRSF7	27.37	1.28	1.20	1.22	1.42	1.00	0.94	0.95	1.11
244	P49902	NT5C2	64.97	0.65	0.59	0.62	0.72	1.00	0.91	0.95	1.11
245	P61981	YWHAG	28.30	0.92	0.85	0.99	1.01	1.00	0.93	1.08	1.10
246	Q92945	KHSRP	73.12	1.12	1.12	1.08	1.24	1.00	1.00	0.96	1.10
247	O60220	TIMM8A	11.00	1.57	0.89	1.56	1.73	1.00	0.56	0.99	1.10
248	P10619	CTSA	54.47	0.39	0.20	0.45	0.43	1.00	0.51	1.16	1.10
249	P04406	GAPDH	36.05	1.23	1.23	1.21	1.36	1.00	0.99	0.98	1.10
250	Q07812	BAX	21.18	0.63	0.40	0.66	0.70	1.00	0.63	1.04	1.10
251	O00151	PDLIM1	36.07	0.58	0.53	0.63	0.64	1.00	0.91	1.08	1.10
252	P06748	NPM1	32.57	1.24	1.24	1.28	1.36	1.00	0.99	1.03	1.10

253	Q13630	TSTA3	35.89	0.79	0.77	0.97	0.86	1.00	0.98	1.24	1.10
254	P55327	TPD52	24.33	1.09	1.09	1.16	1.20	1.00	1.00	1.07	1.10
255	P07951	TPM2	32.85	0.73	0.77	0.79	0.80	1.00	1.05	1.08	1.09
256	P36957	DLST	48.75	0.92	0.90	0.94	1.00	1.00	0.98	1.02	1.09
257	P14618	PKM	57.94	1.12	1.17	1.16	1.22	1.00	1.04	1.03	1.09
258	P21291	CSRP1	20.57	0.69	0.69	0.73	0.76	1.00	0.99	1.05	1.09
259	Q13162	PRDX4	30.54	1.14	1.11	1.16	1.25	1.00	0.97	1.02	1.09
260	P06753	TPM3	32.95	0.91	0.95	1.02	0.99	1.00	1.04	1.12	1.09
261	Q53FA7	TP53I3	35.54	1.03	1.07	1.09	1.12	1.00	1.04	1.05	1.09
262	Q9UQN3	CHMP2B	23.91	1.01	0.90	1.07	1.10	1.00	0.88	1.06	1.08
263	Q96G03	PGM2	68.28	0.86	0.92	0.94	0.93	1.00	1.07	1.09	1.08
264	P62081	RPS7	22.13	1.15	0.92	1.18	1.25	1.00	0.80	1.03	1.08
265	P05388	RPLP0	34.27	1.26	1.20	1.25	1.37	1.00	0.95	1.00	1.08
266	P04179	SOD2	24.72	0.63	0.66	0.67	0.68	1.00	1.05	1.07	1.08
267	P60900	PSMA6	27.40	1.18	1.31	1.22	1.28	1.00	1.11	1.03	1.08
268	P62906	RPL10A	24.83	1.33	1.29	1.34	1.44	1.00	0.97	1.00	1.08
269	Q92599	KIAA0202	55.76	0.86	0.99	0.94	0.92	1.00	1.15	1.10	1.08
270	Q01813	PFKP	85.60	1.24	1.27	1.38	1.33	1.00	1.03	1.11	1.08
271	Q04837	SSBP1	17.26	0.88	1.18	1.23	0.95	1.00	1.34	1.40	1.08
272	P31949	S100A11	11.74	0.83	0.85	0.86	0.89	1.00	1.03	1.04	1.08
273	Q14247	CTTN	61.59	0.78	0.80	0.86	0.84	1.00	1.03	1.10	1.08
274	P04908	HIST1H2AB	14.14	1.12	1.11	1.08	1.20	1.00	0.99	0.97	1.07
275	Q01105	SET	33.49	1.06	1.05	1.15	1.13	1.00	1.00	1.09	1.07
276	O43684	BUB3	37.15	1.14	1.11	1.10	1.23	1.00	0.97	0.96	1.07
277	P30040	ERP29	28.99	1.05	1.02	0.98	1.12	1.00	0.97	0.94	1.07
278	P18124	RPL7	29.23	1.28	1.26	1.14	1.38	1.00	0.98	0.89	1.07
279	P09493	TPM1	32.71	0.72	0.73	0.76	0.77	1.00	1.01	1.05	1.07
280	P25786	PSMA1	29.56	1.18	1.28	1.23	1.27	1.00	1.08	1.04	1.07
281	Q9Y3B8	REXO2	26.83	0.87	0.77	1.00	0.93	1.00	0.89	1.15	1.07
282	P06454	PTMA	12.20	1.35	1.37	1.40	1.44	1.00	1.02	1.04	1.07
283	Q14116	IL18	22.33	0.66	0.65	0.71	0.70	1.00	1.00	1.08	1.07
284	Q969Q0	RPL36AL	12.47	1.23	1.22	1.20	1.31	1.00	0.99	0.98	1.07

285	Q16775	HAGH	33.81	0.99	1.03	0.82	1.06	1.00	1.04	0.83	1.07
286	P61604	HSPE1	10.93	1.02	1.07	1.07	1.09	1.00	1.04	1.05	1.07
287	P00491	PNP	32.12	1.12	1.15	1.13	1.19	1.00	1.03	1.01	1.07
288	P06703	S100A6	10.18	1.01	1.00	1.00	1.07	1.00	0.99	0.99	1.07
289	Q00688	FKBP3	25.18	1.00	1.00	1.09	1.06	1.00	1.01	1.09	1.07
290	Q14444	CAPRIN1	78.37	0.99	1.13	1.12	1.05	1.00	1.14	1.13	1.06
291	P31946	YWHAB	28.08	0.93	0.95	0.99	0.99	1.00	1.02	1.06	1.06
292	P51858	HDGF	26.79	0.99	0.95	1.10	1.05	1.00	0.96	1.11	1.06
293	P36551	CPOX	50.15	1.03	0.88	1.06	1.10	1.00	0.85	1.03	1.06
294	P60660	MYL6	16.93	0.79	0.89	0.68	0.84	1.00	1.12	0.85	1.06
295	Q53H82	LACTB2	32.81	0.74	0.91	0.80	0.78	1.00	1.22	1.08	1.06
296	P0CAP2	POLR2M	41.74	1.12	1.46	1.35	1.18	1.00	1.31	1.21	1.06
297	Q8NC51	SERBP1	44.97	1.37	1.31	1.35	1.45	1.00	0.96	0.99	1.06
298	P00505	GOT2	47.52	0.99	0.95	1.05	1.05	1.00	0.95	1.06	1.06
299	P63000	RAC1	21.45	0.93	0.86	0.92	0.99	1.00	0.92	0.99	1.06
300	P30046	DDT	12.71	1.10	1.33	1.22	1.16	1.00	1.21	1.10	1.06
301	P61353	RPL27	15.80	1.21	1.19	1.16	1.28	1.00	0.98	0.95	1.06
302	Q02878	RPL6	32.73	1.22	1.24	1.22	1.29	1.00	1.01	1.00	1.06
303	Q9Y3X0	CCDC9	59.70	1.21	1.16	1.87	1.28	1.00	0.95	1.54	1.06
304	P62304	SNRPE	10.80	1.13	0.94	1.17	1.19	1.00	0.84	1.03	1.05
305	Q9H2U2	PPA2	37.92	0.82	0.95	0.62	0.87	1.00	1.16	0.76	1.05
306	Q06323	PSME1	28.72	0.75	0.72	0.64	0.79	1.00	0.95	0.85	1.05
307	P60981	DSTN	18.51	0.81	0.76	0.55	0.85	1.00	0.94	0.68	1.05
308	P28066	PSMA5	26.41	1.18	1.33	1.17	1.24	1.00	1.13	0.99	1.05
309	Q16881	TXNRD1	70.86	1.03	1.07	1.10	1.09	1.00	1.03	1.07	1.05
310	P42126	ECI1	32.82	0.80	0.93	0.85	0.84	1.00	1.16	1.06	1.05
311	P06733	ENO1	47.17	0.97	0.95	1.00	1.02	1.00	0.98	1.04	1.05
312	P52943	CRIP2	22.49	0.91	0.88	0.91	0.96	1.00	0.96	0.99	1.05
313	Q15056	EIF4H	27.38	1.08	1.17	1.23	1.13	1.00	1.08	1.14	1.05
314	P54727	RAD23B	43.17	1.12	1.19	1.19	1.18	1.00	1.07	1.06	1.05
315	P62633	CNBP	19.46	1.11	1.04	1.13	1.16	1.00	0.93	1.02	1.05
316	P62750	RPL23A	17.69	1.20	1.21	1.28	1.26	1.00	1.01	1.07	1.05

317	P68104	EEF1A1	50.14	1.14	1.07	1.19	1.20	1.00	0.94	1.05	1.05
318	Q32MZ4	LRRFIP1	89.25	1.22	1.26	1.27	1.28	1.00	1.03	1.04	1.05
319	O60814	HIST1H2BK	13.89	1.07	1.01	1.03	1.12	1.00	0.94	0.96	1.05
320	P60866	RPS20	13.37	1.17	0.95	1.16	1.23	1.00	0.82	0.99	1.05
321	P28072	PSMB6	25.36	1.24	1.35	1.30	1.30	1.00	1.09	1.05	1.05
322	P53004	BLVRA	33.43	0.71	0.76	0.80	0.75	1.00	1.06	1.13	1.05
323	Q05682	CALD1	93.23	0.56	0.75	0.62	0.59	1.00	1.33	1.09	1.05
324	P62140	PPP1CB	37.19	1.09	0.98	1.00	1.14	1.00	0.90	0.92	1.05
325	P50238	CRIP1	8.53	0.92	0.84	0.81	0.96	1.00	0.92	0.88	1.05
326	O15347	HMGB3	22.98	1.27	1.28	1.58	1.33	1.00	1.00	1.24	1.05
327	Q6GMV3	PTRHD1	15.81	0.93	1.59	0.91	0.97	1.00	1.71	0.98	1.05
328	Q9H299	SH3BGRL3	10.44	1.20	1.08	1.19	1.25	1.00	0.90	1.00	1.04
329	Q9H444	CHMP4B	24.95	0.82	0.80	0.94	0.85	1.00	0.98	1.15	1.04
330	P35241	RDX	68.56	0.99	1.00	1.05	1.03	1.00	1.01	1.06	1.04
331	Q8IVF2	AHNAK2	616.62	0.81	0.73	0.81	0.84	1.00	0.90	1.01	1.04
332	P62258	YWHAE	29.17	1.07	1.14	1.34	1.11	1.00	1.06	1.25	1.04
333	P62857	RPS28	7.84	1.21	1.29	1.29	1.26	1.00	1.06	1.07	1.04
334	P23528	CFL1	18.50	1.02	0.96	0.86	1.06	1.00	0.94	0.84	1.04
335	O14737	PDCD5	14.28	1.41	1.38	1.47	1.46	1.00	0.98	1.04	1.04
336	P46779	RPL28	15.75	1.18	1.49	1.10	1.23	1.00	1.26	0.93	1.04
337	Q96AT9	RPE	24.93	1.12	1.09	1.02	1.16	1.00	0.97	0.91	1.04
338	Q16719	KYNU	52.35	1.18	1.17	1.13	1.23	1.00	0.99	0.95	1.04
339	Q09666	AHNAK	629.10	0.93	0.85	0.87	0.97	1.00	0.91	0.93	1.04
340	Q13867	BLMH	52.56	1.18	1.26	1.25	1.23	1.00	1.07	1.06	1.04
341	P06576	ATP5B	56.56	1.04	1.00	1.08	1.08	1.00	0.96	1.04	1.04
342	Q04760	GLO1	20.78	1.03	1.13	1.08	1.06	1.00	1.10	1.05	1.04
343	P29401	TKT	67.88	0.77	0.80	0.81	0.80	1.00	1.03	1.04	1.03
344	P10253	GAA	105.32	0.42	0.58	0.44	0.44	1.00	1.38	1.05	1.03
345	P50990	CCT8	59.62	1.31	1.34	1.32	1.35	1.00	1.02	1.00	1.03
346	Q9HCC0	MCCC2	61.33	1.01	0.77	0.81	1.04	1.00	0.76	0.80	1.03
347	Q16555	DPYSL2	62.29	1.02	1.05	1.05	1.05	1.00	1.03	1.03	1.03
348	Q9UNX3	RPL26L1	17.26	1.13	1.17	1.17	1.17	1.00	1.03	1.04	1.03

349	P10599	TXN	11.74	0.92	0.88	0.90	0.94	1.00	0.96	0.98	1.03
350	O43809	NUDT21	26.23	1.41	1.10	1.09	1.45	1.00	0.78	0.78	1.03
351	P14174	MIF	12.48	0.95	0.96	0.98	0.98	1.00	1.01	1.03	1.03
352	P27105	STOM	31.73	0.59	0.51	0.67	0.61	1.00	0.86	1.14	1.03
353	P14678	SNRPB	24.61	1.13	1.19	1.19	1.16	1.00	1.05	1.06	1.03
354	Q13509	TUBB3	50.43	1.25	1.23	1.25	1.28	1.00	0.99	1.00	1.03
355	P84103	SRSF3	19.33	1.30	1.20	1.25	1.33	1.00	0.92	0.97	1.03
356	O43707	ACTN4	104.85	0.78	0.78	0.78	0.80	1.00	1.01	1.00	1.02
357	O75874	IDH1	46.66	0.72	0.72	0.70	0.74	1.00	1.00	0.98	1.02
358	P63261	ACTG1	41.79	0.82	0.89	0.89	0.84	1.00	1.09	1.08	1.02
359	P00352	ALDH1A1	54.86	0.80	0.76	0.78	0.81	1.00	0.96	0.97	1.02
360	P62241	RPS8	24.21	1.23	1.27	1.12	1.26	1.00	1.03	0.91	1.02
361	O15212	PFDN6	14.58	1.46	1.42	1.37	1.49	1.00	0.97	0.93	1.02
362	P67936	TPM4	28.52	0.63	0.63	0.65	0.64	1.00	1.00	1.03	1.02
363	Q15942	ZYX	61.28	0.74	0.74	0.70	0.75	1.00	0.99	0.94	1.02
364	O75347	TBCA	12.85	1.27	1.29	1.28	1.29	1.00	1.02	1.01	1.02
365	O43570	CA12	39.45	1.89	1.96	1.71	1.92	1.00	1.04	0.91	1.02
366	P09104	ENO2	47.27	1.07	1.00	1.07	1.08	1.00	0.94	1.00	1.02
367	P23526	AHCY	47.72	1.25	1.23	1.17	1.26	1.00	0.99	0.94	1.01
368	O60701	UGDH	55.02	0.91	0.91	0.85	0.92	1.00	1.00	0.94	1.01
369	P27348	YWHAQ	27.76	1.05	1.05	1.08	1.07	1.00	1.00	1.03	1.01
370	P52907	CAPZA1	32.92	0.96	0.89	1.09	0.97	1.00	0.92	1.13	1.01
371	Q99536	VAT1	41.92	0.60	0.60	0.63	0.61	1.00	1.00	1.04	1.01
372	P46776	RPL27A	16.56	1.28	1.22	1.36	1.29	1.00	0.96	1.07	1.01
373	Q15181	PPA1	32.66	1.12	1.04	0.91	1.13	1.00	0.92	0.81	1.01
374	P25789	PSMA4	29.48	1.21	1.28	1.26	1.23	1.00	1.06	1.04	1.01
375	P40429	RPL13A	23.58	1.14	1.11	1.23	1.16	1.00	0.97	1.08	1.01
376	P23141	CES1	62.52	0.80	0.81	0.80	0.80	1.00	1.01	1.01	1.01
377	P00390	GSR	56.26	1.07	1.01	1.05	1.08	1.00	0.95	0.98	1.01
378	P07737	PFN1	15.05	1.06	1.10	1.04	1.07	1.00	1.04	0.98	1.01
379	P18669	PGAM1	28.80	1.22	1.21	1.23	1.24	1.00	0.99	1.00	1.01
380	P09525	ANXA4	35.88	0.56	0.64	0.73	0.57	1.00	1.14	1.30	1.01

381	P40926	MDH2	35.50	1.03	0.98	1.00	1.04	1.00	0.96	0.98	1.01
382	P07195	LDHB	36.64	1.11	1.10	1.10	1.12	1.00	0.99	0.99	1.01
383	Q7Z4V5	HDGFRP2	74.32	1.21	1.09		1.22	1.00	0.90	0.00	1.01
384	Q9H773	DCTPP1	18.68	1.49	1.37	1.50	1.50	1.00	0.92	1.01	1.01
385	P00492	HPRT1	24.58	1.24	1.18	1.30	1.25	1.00	0.95	1.05	1.01
386	Q9UNN8	PROCR	26.67	0.92	1.03	0.94	0.93	1.00	1.11	1.02	1.01
387	P80723	BASP1	22.69	0.94	0.84	0.97	0.95	1.00	0.89	1.03	1.01
388	Q9BWD1	ACAT2	41.35	1.10	0.96	1.13	1.11	1.00	0.87	1.03	1.00
389	O00233	PSMD9	24.68	0.98	0.97	0.90	0.98	1.00	0.99	0.92	1.00
390	P09382	LGALS1	14.72	1.12	1.07	1.27	1.13	1.00	0.95	1.13	1.00
391	Q8NFJ5	GPRC5A	40.25	0.61	0.56	0.64	0.62	1.00	0.91	1.04	1.00
392	P62805	HIST1H4A	11.37	1.27	1.17	1.08	1.27	1.00	0.92	0.85	1.00
393	Q15907	RAB11B	24.49	1.07	1.03	0.98	1.08	1.00	0.96	0.91	1.00
394	P43034	PAFAH1B1	46.64	1.21	1.15	1.21	1.21	1.00	0.95	1.00	1.00
395	P62269	RPS18	17.72	1.19	1.21	1.18	1.19	1.00	1.02	0.99	1.00
396	P40925	MDH1	36.43	0.85	0.85	0.82	0.85	1.00	1.00	0.97	1.00
397	P48163	ME1	64.15	0.85	0.91	0.87	0.85	1.00	1.07	1.02	1.00
398	P10155	TROVE2	60.67	0.86	0.81	0.85	0.86	1.00	0.95	0.99	1.00
399	O60869	EDF1	16.37	1.00	1.20	1.08	1.00	1.00	1.21	1.09	1.00
400	P61970	NUTF2	14.48	1.00	1.01	0.96	1.00	1.00	1.01	0.96	1.00
401	Q00796	SORD	38.32	1.12	1.25	1.52	1.12	1.00	1.11	1.36	1.00
402	P07602	PSAP	58.11	0.48	0.46	0.48	0.48	1.00	0.97	1.01	1.00
403	P30050	RPL12	17.82	1.25	1.20	1.26	1.25	1.00	0.96	1.00	1.00
404	P46821	MAP1B	270.63	1.01	1.05	0.90	1.01	1.00	1.04	0.89	1.00
405	P15531	NME1	17.15	1.22	1.19	1.23	1.22	1.00	0.97	1.00	1.00
406	P30086	PEBP1	21.06	0.84	0.80	0.87	0.84	1.00	0.95	1.04	1.00
407	P62310	LSM3	11.85	1.10	1.22	1.09	1.10	1.00	1.11	0.99	1.00
408	Q99497	PARK7	19.89	1.01	1.01	1.04	1.00	1.00	1.00	1.03	1.00
409	P06899	HIST1H2BJ	13.90	1.19	1.06	1.11	1.18	1.00	0.89	0.93	1.00
410	O43399	TPD52L2	22.24	0.93	0.94	0.96	0.92	1.00	1.01	1.03	0.99
411	P60174	TPI1	30.79	1.14	1.16	1.09	1.13	1.00	1.02	0.96	0.99
412	P62917	RPL8	28.02	1.26	1.22	1.18	1.25	1.00	0.96	0.94	0.99

413	P61247	RPS3A	29.94	1.20	1.23	1.07	1.19	1.00	1.03	0.89	0.99
414	Q92804	TAF15	61.83	1.17	1.18	1.16	1.16	1.00	1.01	0.99	0.99
415	P09622	DLD	54.18	1.00	0.97	0.94	0.99	1.00	0.96	0.93	0.99
416	Q9UJZ1	STOML2	38.53	1.18	1.36	1.31	1.17	1.00	1.15	1.11	0.99
417	P49006	MARCKSL1	19.53	1.03	0.91	0.98	1.02	1.00	0.88	0.95	0.99
418	P04075	ALDOA	39.42	1.41	1.37	1.40	1.39	1.00	0.98	0.99	0.99
419	Q12905	ILF2	43.06	1.10	1.29	1.05	1.09	1.00	1.17	0.96	0.99
420	P42766	RPL35	14.55	1.17	1.19	1.15	1.16	1.00	1.02	0.99	0.99
421	Q13011	ECH1	35.82	0.80	0.83	0.76	0.79	1.00	1.04	0.96	0.99
422	P31948	STIP1	62.64	1.23	1.22	1.27	1.22	1.00	0.99	1.03	0.99
423	P62899	RPL31	14.46	1.16	1.12	1.16	1.14	1.00	0.97	1.01	0.99
424	P18206	VCL	123.80	0.84	0.80	0.82	0.83	1.00	0.96	0.98	0.99
425	P67809	YBX1	35.92	1.53	1.62	1.46	1.50	1.00	1.06	0.96	0.98
426	O14818	PSMA7	27.89	1.23	1.56	1.24	1.21	1.00	1.27	1.01	0.98
427	Q9UKY7	CDV3	27.33	1.04	1.38	1.15	1.02	1.00	1.33	1.11	0.98
428	P48059	LIMS1	37.25	0.79	0.85	0.70	0.78	1.00	1.07	0.88	0.98
429	P17858	PFKL	85.02	1.01	0.74	0.88	0.99	1.00	0.73	0.87	0.98
430	P09496	CLTA	27.08	1.10	1.07	1.05	1.09	1.00	0.96	0.95	0.98
431	P35754	GLRX	11.78	0.57	0.55	0.54	0.56	1.00	0.96	0.96	0.98
432	P13639	EEF2	95.34	1.29	1.28	1.27	1.27	1.00	0.99	0.98	0.98
433	P59998	ARPC4	19.67	0.93	0.98	1.08	0.91	1.00	1.06	1.17	0.98
434	P62136	PPP1CA	37.51	1.06	1.02	1.16	1.04	1.00	0.96	1.10	0.98
435	Q07020	RPL18	21.63	1.15	1.20	1.23	1.12	1.00	1.05	1.07	0.98
436	P35269	GTF2F1	58.24	1.29	1.41	1.50	1.27	1.00	1.09	1.16	0.98
437	P49748	ACADVL	70.39	0.96	0.99	0.98	0.94	1.00	1.03	1.02	0.98
438	Q9BRF8	CPPED1	35.55	0.80	0.74	0.71	0.78	1.00	0.93	0.89	0.98
439	P00338	LDHA	36.69	1.46	1.46	1.35	1.42	1.00	1.00	0.92	0.98
440	Q9C0G6	DNAH6	475.98	1.28	1.22	0.87	1.25	1.00	0.95	0.68	0.98
441	P61457	PCBD1	12.00	1.10	1.03	1.14	1.08	1.00	0.93	1.04	0.98
442	Q9Y3F4	STRAP	38.44	1.57	1.87	1.49	1.53	1.00	1.19	0.95	0.98
443	O14950	MYL12B	19.78	0.76	0.97	0.60	0.74	1.00	1.28	0.79	0.98
444	P08237	PFKM	85.18	1.03	0.81	0.94	1.01	1.00	0.79	0.91	0.98

445	P07384	CAPN1	81.89	0.84	0.77	0.78	0.82	1.00	0.92	0.92	0.98
446	P11021	HSPA5	72.33	1.16	1.15	0.97	1.13	1.00	0.99	0.84	0.98
447	P16989	YBX3	40.09	1.30	1.11	1.02	1.26	1.00	0.85	0.78	0.97
448	P12814	ACTN1	103.06	0.82	0.80	0.80	0.80	1.00	0.97	0.98	0.97
449	Q9GZP8	IMUP	10.90	0.95	0.84	1.27	0.92	1.00	0.88	1.34	0.97
450	P47755	CAPZA2	32.95	0.54	0.01	0.84	0.53	1.00	0.02	1.55	0.97
451	P51148	RAB5C	23.48	1.11	1.05	1.06	1.08	1.00	0.94	0.95	0.97
452	P30044	PRDX5	22.09	0.75	0.71	0.77	0.73	1.00	0.94	1.03	0.97
453	O00764	PDXK	35.10	0.71	0.76	0.71	0.69	1.00	1.07	1.01	0.97
454	P62280	RPS11	18.43	1.28	1.22	1.26	1.24	1.00	0.95	0.98	0.97
455	P25788	PSMA3	28.43	1.28	1.11	1.31	1.24	1.00	0.87	1.02	0.97
456	A6NCE7	MAP1LC3B2	14.63	0.68	0.70	1.08	0.65	1.00	1.04	1.60	0.97
457	Q14847	LASP1	29.72	0.90	0.99	0.86	0.87	1.00	1.10	0.96	0.97
458	Q16531	DDB1	126.97	1.00	0.97	1.00	0.97	1.00	0.97	0.99	0.97
459	P62158	CALM1	16.84	0.91	0.94	0.91	0.88	1.00	1.04	1.00	0.97
460	P0C0S5	H2AFZ	13.55	1.16	1.07	1.07	1.12	1.00	0.92	0.92	0.97
461	P49773	HINT1	13.80	0.93	0.97	1.00	0.90	1.00	1.04	1.07	0.97
462	O15511	ARPC5	16.32	0.96	0.93	0.92	0.93	1.00	0.97	0.95	0.96
463	O14979	HNRNPDL	46.44	1.16	1.57	1.59	1.11	1.00	1.36	1.37	0.96
464	Q9ULC4	MCTS1	20.56	1.27	1.24	0.99	1.22	1.00	0.98	0.78	0.96
465	P61289	PSME3	29.51	1.44	1.52	1.47	1.38	1.00	1.05	1.02	0.96
466	P46781	RPS9	22.59	1.24	1.23	1.20	1.20	1.00	0.98	0.96	0.96
467	P26447	S100A4	11.73	0.78	0.79	0.78	0.75	1.00	1.01	0.99	0.96
468	O43768	ENSA	13.39	1.41	1.22	1.24	1.36	1.00	0.86	0.88	0.96
469	O15382	BCAT2	44.29	1.01	1.03	0.89	0.97	1.00	1.02	0.89	0.96
470	P11166	SLC2A1	54.08	1.65	1.42	1.60	1.58	1.00	0.86	0.97	0.96
471	Q9Y3C8	UFC1	19.46	1.01	0.95	0.98	0.97	1.00	0.94	0.97	0.96
472	P61313	RPL15	24.15	1.25	1.20	1.26	1.20	1.00	0.96	1.01	0.96
473	Q8IUE6	HIST2H2AB	14.00	1.23	1.15	1.07	1.18	1.00	0.94	0.87	0.96
474	Q9UK76	HN1	16.01	1.64	1.52	2.15	1.56	1.00	0.93	1.31	0.96
475	Q9NNW7	TXNRD2	56.46	0.90	0.89	0.75	0.86	1.00	0.99	0.83	0.96
476	P43487	RANBP1	23.31	1.41	1.43	1.65	1.34	1.00	1.02	1.18	0.95

477	O60664	PLIN3	47.07	0.77	0.86	0.98	0.74	1.00	1.11	1.27	0.95
478	P37108	SRP14	14.57	1.29	1.07	1.19	1.23	1.00	0.83	0.92	0.95
479	P55072	VCP	89.32	1.20	1.11	1.07	1.14	1.00	0.92	0.89	0.95
480	P08708	RPS17	15.55	1.24	1.38	1.20	1.18	1.00	1.11	0.97	0.95
481	P63173	RPL38	8.22	1.42	1.41	1.24	1.35	1.00	0.99	0.88	0.95
482	P56537	EIF6	26.60	1.05	1.09	0.97	1.00	1.00	1.04	0.92	0.95
483	Q93052	LPP	65.75	0.55	0.59	0.58	0.52	1.00	1.08	1.07	0.95
484	P46926	GNPDA1	32.67	0.98	0.90	0.95	0.93	1.00	0.92	0.97	0.95
485	Q71DI3	HIST2H3A;	15.39	1.29	1.13	1.09	1.22	1.00	0.87	0.84	0.95
486	P62942	FKBP1A	11.95	1.14	1.03	1.10	1.08	1.00	0.90	0.96	0.95
487	P18084	ITGB5	88.05	0.57	0.46	0.74	0.54	1.00	0.80	1.30	0.95
488	P82979	SARNP	23.67	1.17	1.03	0.92	1.10	1.00	0.89	0.79	0.95
489	Q12906	ILF3	95.34	1.20	1.08	1.08	1.14	1.00	0.90	0.90	0.95
490	Q9Y520	PRRC2C	316.91	1.42	1.24	1.32	1.34	1.00	0.87	0.93	0.95
491	Q96M27	PRRC1	46.70	0.83	0.81		0.78	1.00	0.98	0.00	0.94
492	P23396	RPS3	26.69	1.11	1.12	1.11	1.05	1.00	1.01	1.00	0.94
493	P41250	GARS	83.16	1.23	1.33	1.29	1.16	1.00	1.08	1.05	0.94
494	P46976	GYG1	39.38	1.08	0.43	0.98	1.02	1.00	0.39	0.91	0.94
495	P20618	PSMB1	26.49	1.31	1.32	1.56	1.23	1.00	1.01	1.19	0.94
496	P62424	RPL7A	30.00	1.22	1.16	1.12	1.15	1.00	0.95	0.92	0.94
497	O95831	AIFM1	66.90	1.21	1.10	1.03	1.14	1.00	0.91	0.86	0.94
498	P22392	NME2	17.30	1.20	1.17	1.15	1.12	1.00	0.98	0.96	0.94
499	Q15404	RSU1	31.54	0.75	0.97	0.86	0.70	1.00	1.29	1.15	0.94
500	P62314	SNRPD1	13.28	1.23	1.16	1.23	1.15	1.00	0.95	1.00	0.93
501	P46778	RPL21	18.56	1.31	1.21	1.20	1.22	1.00	0.93	0.92	0.93
502	Q9UMX5	NENF	18.86	1.39	0.84	1.19	1.29	1.00	0.60	0.86	0.93
503	Q9Y4Z0	LSM4	15.35	1.12	1.00	1.32	1.04	1.00	0.89	1.17	0.93
504	P39023	RPL3	46.11	1.23	1.27	1.19	1.15	1.00	1.03	0.97	0.93
505	P62316	SNRPD2	13.53	1.17	1.36	1.11	1.08	1.00	1.16	0.95	0.93
506	P17655	CAPN2	79.99	0.89	0.86	0.89	0.82	1.00	0.96	0.99	0.92
507	P22234	PAICS	47.08	1.49	1.41	1.38	1.37	1.00	0.95	0.93	0.92
508	Q15019	KIAA0158	41.49	0.97	0.82	0.88	0.90	1.00	0.85	0.91	0.92

509	O15143	ARPC1B	40.95	0.79	0.85	0.91	0.73	1.00	1.08	1.16	0.92
510	P17174	GOT1	46.25	1.46	1.43	1.27	1.35	1.00	0.98	0.87	0.92
511	Q9UPT8	ZC3H4	140.26	1.34	3.07	1.15	1.23	1.00	2.29	0.86	0.92
512	P67775	PPP2CA	35.59	1.26	1.27	1.10	1.16	1.00	1.01	0.88	0.92
513	P18077	RPL35A	12.54	1.22	0.94	1.11	1.12	1.00	0.77	0.91	0.92
514	P61006	RAB8A	23.67	1.08	1.31	1.09	0.99	1.00	1.22	1.02	0.92
515	Q14157	UBAP2L	114.53	1.38	0.96	1.34	1.27	1.00	0.70	0.98	0.92
516	P12955	PEPD	54.55	0.72	0.83	0.73	0.67	1.00	1.14	1.01	0.92
517	P07108	DBI	10.04	0.85	0.75	0.85	0.78	1.00	0.89	1.01	0.92
518	P61160	ACTR2	44.76	1.00	1.05	0.99	0.92	1.00	1.05	0.99	0.92
519	Q9BY43	CHMP4A	25.10	1.09	1.03	1.06	1.01	1.00	0.94	0.97	0.92
520	P20810	CAST	76.57	0.80	0.62	0.74	0.74	1.00	0.78	0.92	0.92
521	Q15075	EEA1	162.46	0.81	0.65	0.77	0.74	1.00	0.80	0.96	0.91
522	P35637	FUS	53.43	1.41	1.26	1.27	1.29	1.00	0.90	0.90	0.91
523	Q8NBS9	TXNDC5	47.63	0.97	0.96	0.80	0.89	1.00	0.99	0.82	0.91
524	P15121	AKR1B1	35.85	1.14	1.12	1.12	1.04	1.00	0.98	0.98	0.91
525	P62318	SNRPD3	13.92	1.17	1.12	1.12	1.07	1.00	0.96	0.95	0.91
526	A6NDG6	PGP	34.01	1.55	1.70	1.46	1.42	1.00	1.10	0.94	0.91
527	P78417	GSTO1	27.57	1.02	0.99	1.01	0.93	1.00	0.97	0.99	0.91
528	O15145	ARPC3	20.55	0.83	0.94	1.01	0.75	1.00	1.13	1.22	0.91
529	Q9NSE4	IARS2	113.79	1.26	1.20	1.11	1.15	1.00	0.95	0.88	0.91
530	O75608	LYPLA1	24.67	0.95	1.13	1.05	0.86	1.00	1.19	1.11	0.91
531	Q9UL25	RAB21	24.35	1.29	1.38	1.29	1.16	1.00	1.07	1.00	0.91
532	P53680	AP2S1	17.02	0.88	0.85	0.86	0.80	1.00	0.97	0.98	0.90
533	P62910	RPL32	15.86	1.19	1.19	1.10	1.07	1.00	1.00	0.93	0.90
534	P63208	SKP1	18.66	1.06	1.03	1.05	0.96	1.00	0.97	0.98	0.90
535	Q92522	H1FX	22.49	0.88	1.04	1.21	0.79	1.00	1.19	1.38	0.90
536	P10301	RRAS	23.48	0.87	0.87	0.70	0.78	1.00	1.01	0.81	0.90
537	P26583	HMGB2	24.03	1.35	1.18	1.65	1.21	1.00	0.88	1.22	0.90
538	P51659	HSD17B4	79.69	0.86	0.79	0.79	0.77	1.00	0.92	0.92	0.90
539	P56545	CTBP2	48.94	0.91	1.20	0.79	0.81	1.00	1.33	0.88	0.90
540	Q9H910	HN1L	20.06	1.34	1.21	1.27	1.20	1.00	0.91	0.95	0.90

541	P0DMV9	HSPA1B	70.05	0.91	0.90	0.85	0.82	1.00	0.99	0.94	0.90
542	P46783	RPS10	18.90	1.24	1.14	1.24	1.12	1.00	0.92	1.00	0.90
543	P35268	RPL22	14.79	1.21	1.19	1.13	1.09	1.00	0.98	0.93	0.90
544	P84090	ERH	12.26	1.29	1.04	1.09	1.15	1.00	0.81	0.85	0.90
545	P62913	RPL11	20.25	1.16	1.21	1.02	1.04	1.00	1.05	0.88	0.89
546	P62826	RAN	24.42	1.36	1.22	1.26	1.21	1.00	0.90	0.93	0.89
547	P62851	RPS25	13.74	1.18	1.14	1.17	1.05	1.00	0.97	0.99	0.89
548	P02794	FTH1	21.23	2.60	2.52	2.28	2.31	1.00	0.97	0.88	0.89
549	Q14914	PTGR1	35.87	0.93	0.88	0.88	0.83	1.00	0.94	0.94	0.89
550	Q9BXV9	C14orf142	10.86	1.41	1.37	1.30	1.26	1.00	0.97	0.92	0.89
551	P62277	RPS13	17.22	1.31	1.24	1.25	1.16	1.00	0.95	0.96	0.89
552	P07237	P4HB	57.12	1.03	1.01	0.94	0.91	1.00	0.99	0.91	0.89
553	Q9Y333	LSM2	10.83	1.27	1.56	3.05	1.12	1.00	1.23	2.40	0.88
554	P11413	G6PD	59.26	0.91	0.90	0.89	0.81	1.00	0.99	0.98	0.88
555	P63244	RACK1	35.08	1.26	1.30	1.20	1.11	1.00	1.03	0.95	0.88
556	Q9NP81	SARS2	58.28	0.78	0.68	0.68	0.69	1.00	0.88	0.87	0.88
557	P61026	RAB10	22.54	1.16	1.00	1.03	1.01	1.00	0.87	0.89	0.88
558	Q2TAA2	IAH1	27.60	0.92	0.50	0.01	0.80	1.00	0.54	0.01	0.87
559	Q969E4	TCEAL3	22.50	0.79	0.73	0.64	0.69	1.00	0.91	0.80	0.87
560	P46782	RPS5	22.88	1.13	1.18	1.12	0.99	1.00	1.04	0.99	0.87
561	Q16181	CDC10	50.68	1.05	0.94	0.85	0.91	1.00	0.89	0.80	0.87
562	O95571	ETHE1	27.87	0.76	0.67	0.53	0.66	1.00	0.87	0.69	0.87
563	O60256	PRPSAP2	40.93	2.01	1.73	1.74	1.74	1.00	0.86	0.86	0.87
564	Q02543	RPL18A	20.76	1.22	1.46	1.04	1.05	1.00	1.20	0.85	0.86
565	P61513	RPL37A	10.28	1.24	1.29	1.24	1.07	1.00	1.04	1.00	0.86
566	P11216	PYGB	96.70	0.98	0.95	0.72	0.84	1.00	0.97	0.73	0.86
567	P62847	RPS24	15.42	1.38	1.28	1.18	1.19	1.00	0.93	0.86	0.86
568	P08865	RPSA	32.85	1.32	1.37	1.25	1.14	1.00	1.03	0.94	0.86
569	P29218	IMPA1	30.19	1.22	1.08	1.13	1.05	1.00	0.88	0.92	0.86
570	P38117	ETFB	27.84	0.97	0.94	0.95	0.84	1.00	0.96	0.97	0.86
571	P61163	ACTR1A	42.61	1.00	1.04	1.13	0.86	1.00	1.04	1.12	0.86
572	Q14192	FHL2	32.19	0.60	0.50	0.51	0.52	1.00	0.83	0.85	0.86

573	P09211	GSTP1	23.36	0.82	0.85	0.72	0.71	1.00	1.03	0.87	0.86
574	O43852	CALU	37.11	1.43	1.14	1.18	1.23	1.00	0.80	0.83	0.86
575	P07711	CTSL	37.56	3.52	12.23	4.14	3.00	1.00	3.48	1.18	0.85
576	O00193	SMAP	20.33	1.20	0.88	0.73	1.02	1.00	0.74	0.61	0.85
577	P62714	PPP2CB	35.57	1.34	1.21	1.08	1.14	1.00	0.90	0.81	0.85
578	P09417	QDPR	25.79	1.06	1.00	0.94	0.90	1.00	0.95	0.89	0.85
579	P55263	ADK	40.55	1.12	1.41	0.92	0.95	1.00	1.26	0.83	0.85
580	O15144	ARPC2	34.33	1.12	0.98	0.92	0.95	1.00	0.87	0.82	0.84
581	Q96IZ0	PAWR	36.57	0.67	0.44	0.55	0.56	1.00	0.65	0.82	0.84
582	P09110	ACAA1	44.29	1.06	0.85	1.03	0.89	1.00	0.80	0.97	0.84
583	Q96B97	SH3KBP1	73.13	2.20	1.83	3.35	1.85	1.00	0.83	1.52	0.84
584	P37837	TALDO1	37.54	0.82	0.80	0.70	0.69	1.00	0.97	0.86	0.84
585	P10768	ESD	31.46	0.96	0.73	0.76	0.80	1.00	0.77	0.79	0.84
586	P49207	RPL34	13.29	1.45	1.17	1.19	1.21	1.00	0.81	0.82	0.84
587	P11766	ADH5	39.72	1.11	0.26	0.85	0.93	1.00	0.23	0.77	0.83
588	Q15631	TSN	26.18	1.54	1.25	1.15	1.28	1.00	0.81	0.75	0.83
589	Q9Y237	PIN4	13.81	1.14	0.91	1.02	0.95	1.00	0.80	0.89	0.83
590	P34897	SHMT2	55.99	1.23	1.18	1.07	1.02	1.00	0.96	0.87	0.83
591	P49903	SEPHS1	42.91	1.54	1.25	1.30	1.27	1.00	0.81	0.84	0.83
592	Q9UKK9	NUDT5	24.33	1.28	1.35	1.11	1.06	1.00	1.06	0.87	0.83
593	P16070	CD44	81.54	1.43	1.26	1.25	1.18	1.00	0.88	0.87	0.83
594	Q86V81	ALYREF	26.89	1.36	1.26	1.06	1.12	1.00	0.92	0.78	0.82
595	P49720	PSMB3	22.95	1.49	1.74	1.54	1.23	1.00	1.17	1.04	0.82
596	Q13907	IDI1	26.32	1.34	1.10	1.23	1.11	1.00	0.82	0.91	0.82
597	P55145	MANF	20.70	1.45	1.17	1.33	1.19	1.00	0.80	0.92	0.82
598	P31153	MAT2A	43.66	1.11	0.90	0.92	0.91	1.00	0.81	0.83	0.82
599	P05067	APP	86.94	3.48	2.59	3.12	2.84	1.00	0.75	0.90	0.82
600	Q05639	EEF1A2	50.47	1.47	1.24	1.08	1.20	1.00	0.84	0.73	0.82
601	P34896	SHMT1	53.08	1.36	1.04	1.05	1.11	1.00	0.76	0.77	0.82
602	P61019	RAB2A	23.55	1.35	1.02	1.06	1.10	1.00	0.75	0.78	0.81
603	P05556	ITGB1	88.41	1.05	0.70	0.91	0.86	1.00	0.67	0.86	0.81
604	O60218	AKR1B10	36.02	1.08	1.14	1.02	0.87	1.00	1.05	0.94	0.81

605	P23434	GCSH	18.88	1.30	0.86	1.05	1.05	1.00	0.66	0.81	0.81
606	Q99436	PSMB7	29.97	1.65	1.63	1.32	1.33	1.00	0.98	0.80	0.80
607	P06744	GPI	63.15	1.11	1.11	1.09	0.89	1.00	1.00	0.98	0.80
608	P11142	HSPA8	70.90	1.07	1.04	0.98	0.86	1.00	0.97	0.91	0.80
609	Q9Y3U8	RPL36	12.25	1.35	1.37	1.25	1.08	1.00	1.02	0.93	0.80
610	P13804	ETFA	35.08	1.04	0.97	0.99	0.83	1.00	0.94	0.96	0.80
611	Q13428	TCOF1	152.10	2.11	1.51	1.33	1.69	1.00	0.71	0.63	0.80
612	P20042	EIF2S2	38.39	1.88	1.14	1.76	1.49	1.00	0.61	0.94	0.79
613	Q9Y3I0	RTCB	55.21	0.81	0.87	0.56	0.64	1.00	1.08	0.69	0.79
614	P46777	RPL5	34.36	1.27	1.20	1.14	1.00	1.00	0.95	0.90	0.79
615	P42330	AKR1C3	36.85	1.02	0.83	0.81	0.80	1.00	0.81	0.80	0.79
616	Q09028	RBBP4	47.66	1.36	1.29	1.28	1.08	1.00	0.95	0.94	0.79
617	Q9Y617	PSAT1	40.42	1.59	1.55	1.34	1.24	1.00	0.98	0.84	0.78
618	P55957	BID	21.99	1.30	0.83	1.05	1.01	1.00	0.64	0.81	0.78
619	P00367	GLUD1	61.40	0.83	0.69	0.65	0.63	1.00	0.84	0.79	0.77
620	P25398	RPS12	14.51	1.46	1.21	1.23	1.12	1.00	0.83	0.84	0.77
621	Q9NZL9	MAT2B	37.55	1.19	1.05	1.06	0.91	1.00	0.88	0.89	0.77
622	P08195	SLC3A2	67.99	1.81	1.21	1.48	1.38	1.00	0.67	0.82	0.76
623	P54819	AK2	26.48	1.26	1.15	1.15	0.95	1.00	0.91	0.91	0.75
624	P29966	MARCKS	31.55	1.41	0.88	0.85	1.05	1.00	0.62	0.60	0.74
625	P62249	RPS16	16.45	1.31	1.25	1.13	0.97	1.00	0.96	0.86	0.74
626	P61106	RAB14	23.90	1.18	1.02	1.01	0.87	1.00	0.86	0.86	0.74
627	P00558	PGK1	44.61	0.92	0.96	1.00	0.67	1.00	1.04	1.09	0.73
628	E9PAV3	NACA	205.42	1.17	1.06	1.28	0.86	1.00	0.90	1.09	0.73
629	Q9UJ70	NAGK	37.38	0.85	0.73	0.58	0.61	1.00	0.86	0.69	0.72
630	O95861	BPNT1	33.39	0.92	0.65	0.60	0.66	1.00	0.71	0.65	0.72
631	Q9BTZ2	DHRS4	29.54	0.84	1.07	0.66	0.60	1.00	1.27	0.79	0.72
632	Q96HN2	AHCYL2	66.72	1.38	1.07	0.81	0.99	1.00	0.78	0.59	0.72
633	Q96CT7	CCDC124	25.84	1.58	1.70	1.49	1.13	1.00	1.08	0.95	0.71
634	Q9NVS9	PNPO	29.99	1.15	1.31	0.96	0.82	1.00	1.13	0.84	0.71
635	P18621	RPL17	21.40	1.12	1.13	1.14	0.80	1.00	1.01	1.01	0.71
636	Q96KP4	CNDP2	52.88	0.95	0.89	0.90	0.68	1.00	0.93	0.94	0.71

637	P28070	PSMB4	29.20	2.19	1.79	1.45	1.53	1.00	0.82	0.66	0.70
638	P84098	RPL19	23.47	1.03	1.06	0.99	0.72	1.00	1.02	0.96	0.70
639	P63151	PPP2R2A	51.69	1.91	1.27	1.37	1.31	1.00	0.67	0.72	0.69
640	O43598	DNPH1	19.11	1.05	0.65	0.69	0.72	1.00	0.62	0.66	0.69
641	Q5TFE4	NT5DC1	51.84	1.48	1.60	1.09	1.01	1.00	1.09	0.74	0.68
642	P30566	ADSL	54.89	1.37	4.31	1.30	0.93	1.00	3.14	0.95	0.68
643	Q04828	AKR1C1	36.79	1.13	0.85	0.83	0.76	1.00	0.75	0.73	0.67
644	P63220	RPS21	9.11	1.54	1.35	1.16	1.02	1.00	0.88	0.75	0.67
645	P52895	AKR1C2	36.73	1.13	0.79	0.98	0.75	1.00	0.70	0.87	0.66
646	P21281	ATP6V1B2	56.50	1.95	1.09	1.09	1.29	1.00	0.56	0.56	0.66
647	Q01844	EWSR1	68.48	1.40	1.48	1.26	0.91	1.00	1.06	0.90	0.65
648	P26006	ITGA3	116.61	1.47	0.61	1.31	0.95	1.00	0.42	0.89	0.65
649	P49189	ALDH9A1	53.80	1.45	1.02	1.08	0.94	1.00	0.70	0.74	0.65
650	P30042	C21orf33	28.17	1.21	0.84	0.80	0.76	1.00	0.69	0.66	0.63
651	Q9UHV9	PFDN2	16.65	2.38	1.30	1.50	1.47	1.00	0.55	0.63	0.62
652	Q9H0U4	RAB1B	22.17	1.38	0.98	0.94	0.84	1.00	0.71	0.68	0.61
653	P16152	CBR1	30.37	1.03	0.96	0.84	0.63	1.00	0.93	0.81	0.61
654	P62306	SNRPF	9.73	1.81	1.26	1.31	1.10	1.00	0.70	0.72	0.61
655	Q5RKV6	EXOSC6	28.23	2.12	1.27	1.42	1.29	1.00	0.60	0.67	0.61
656	P49721	PSMB2	22.84	1.89	1.37	1.36	1.13	1.00	0.73	0.72	0.60
657	Q92820	GGH	35.96	0.97	0.36	0.92	0.58	1.00	0.37	0.95	0.59
658	P52788	SMS	41.27	1.36	1.26	0.81	0.80	1.00	0.92	0.60	0.59
659	P52815	MRPL12	21.35	1.99	1.78	1.03	1.16	1.00	0.89	0.52	0.59
660	P28838	LAP3	56.17	1.11	0.82	0.61	0.65	1.00	0.74	0.55	0.58
661	Q93099	HGD	49.96	2.15	1.24	1.21	1.24	1.00	0.58	0.56	0.58
662	Q12860	CNTN1	113.32	1.15	0.84	0.84	0.66	1.00	0.73	0.73	0.57
663	P38646	HSPA9	73.68	1.13	1.11	0.94	0.64	1.00	0.98	0.83	0.56
664	Q9Y265	RUVBL1	50.23	2.02	1.15	1.02	1.13	1.00	0.57	0.50	0.56
665	Q13442	PDAP1	20.63	1.85	1.43	1.43	1.02	1.00	0.77	0.77	0.55
666	P07099	EPHX1	52.95	1.61	0.30	0.95	0.84	1.00	0.19	0.59	0.52
667	Q07021	C1QBP	31.36	1.47	1.23		0.76	1.00	0.84	0.00	0.52
668	P42166	TMPO	75.49	1.67	0.78	0.56	0.83	1.00	0.47	0.33	0.50

669	Q9BX68	HINT2	17.16	1.57	0.75	1.02	0.78	1.00	0.48	0.65	0.50
670	Q16698	DECR1	36.07	1.23	0.77	0.66	0.61	1.00	0.62	0.53	0.50
671	O43143	DHX15	90.93	1.71	1.31	1.06	0.84	1.00	0.76	0.62	0.49
672	Q96GK7	FAHD2A	34.60	1.40	0.55	0.65	0.68	1.00	0.40	0.47	0.48
673	P55036	PSMD4	40.74	1.62	1.03	1.41	0.78	1.00	0.64	0.87	0.48
674	Q7Z4H3	HDDC2	23.39	2.71	0.94	0.91	1.30	1.00	0.35	0.34	0.48
675	Q08257	CRYZ	35.21	0.93	0.82	0.85	0.44	1.00	0.89	0.91	0.47
676	P52888	THOP1	78.84	3.77	1.92	1.54	1.74	1.00	0.51	0.41	0.46
677	Q08209	PPP3CA	58.69	1.06	0.99	0.84	0.49	1.00	0.93	0.79	0.46
678	P06737	PYGL	97.15	1.39	0.80	1.44	0.59	1.00	0.58	1.04	0.43
679	Q9ULA0	DNPEP	52.43	2.28	2.14	1.01	0.96	1.00	0.94	0.45	0.42
680	Q15843	NEDD8	9.07	2.55	1.06	1.18	1.06	1.00	0.41	0.46	0.42
681	Q9HB90	RRAGC	44.22	2.14	0.72	0.66	0.85	1.00	0.34	0.31	0.40
682	Q8WWM7	ATXN2L	113.37	1.65	1.67	1.35	0.65	1.00	1.01	0.82	0.40
683	Q13510	ASAH1	44.66	1.39	0.54	0.49	0.55	1.00	0.39	0.35	0.39
684	P04181	OAT	48.53	1.07	1.29	0.96	0.42	1.00	1.21	0.90	0.39
685	P60842	EIF4A1	46.15	1.24	1.12	0.76	0.47	1.00	0.90	0.62	0.38
686	P11310	ACADM	46.59	2.03	0.68	0.66	0.70	1.00	0.34	0.32	0.34
687	P49023	PXN	64.50	1.86	0.97	0.44	0.62	1.00	0.52	0.24	0.33
688	P06865	HEXA	60.70	1.14	0.64	0.54	0.33	1.00	0.56	0.48	0.29
689	P35611	ADD1	80.95	0.57	1.61	1.22	0.11	1.00	2.82	2.14	0.20
690	P09012	SNRPA	31.28	14.15	22.16	30.38	1.75	1.00	1.57	2.15	0.12
691	Q9BYT8	NLN	80.65	0.89	0.85	0.30	0.10	1.00	0.96	0.34	0.11
692	O75940	SMNDC1	26.71	9.42	0.30	1.07	0.98	1.00	0.03	0.11	0.10
693	O00625	PIR	32.11	0.89	1.02		0.01	1.00	1.14	0.00	0.01
694	P41227	NAA10	26.46	0.46	1.46	100.00		1.00	3.17	216.92	0.00
695	Q9BXJ9	NAA15	101.27	0.49	1.36	100.00		1.00	2.81	205.76	0.00
696	P08727	KRT19	44.11	0.72	3.97	100.00		1.00	5.54	139.47	0.00
697	Q15365	PCBP1	37.50	1.01	1.04	100.00		1.00	1.03	99.01	0.00
698	Q00610	CLTC	191.61	1.02	1.07	100.00		1.00	1.05	98.14	0.00
699	O60506	SYNCRIP	69.60	1.19	1.28	100.00		1.00	1.07	84.03	0.00
700	O95881	TXNDC12	19.21	1.28	1.27	100.00		1.00	0.99	78.00	0.00

701	P29692	EEF1D	31.12	1.62	1.90	100.00		1.00	1.17	61.80	0.00
702	P13995	MTHFD2	37.89	2.57	0.83	100.00		1.00	0.32	38.87	0.00
703	Q13501	SQSTM1	47.69	2.92	3.65	50.65		1.00	1.25	17.33	0.00
704	P22061	PCMT1	24.64	0.96	1.16	3.79		1.00	1.20	3.93	0.00
705	P15170	GSPT1	55.76	1.42	1.29	4.72		1.00	0.91	3.32	0.00
706	P31689	DNAJA1	44.87	1.04	1.19	2.99		1.00	1.14	2.88	0.00
707	Q16543	CDC37	44.47	1.50	1.35	4.08		1.00	0.90	2.72	0.00
708	Q9Y295	DRG1	40.54	1.04	1.02	2.36		1.00	0.98	2.28	0.00
709	P06730	EIF4E	25.10	1.53	1.32	3.33		1.00	0.86	2.18	0.00
710	O00410	IPO5	123.63	1.85	1.02	3.98		1.00	0.55	2.16	0.00
711	P04080	CSTB	11.14	0.75	0.92	1.54		1.00	1.23	2.07	0.00
712	O95373	IPO7	119.52	1.49	1.74	2.80		1.00	1.16	1.88	0.00
713	Q03426	MVK	42.45	0.47	1.12	0.86		1.00	2.38	1.83	0.00
714	Q9NQW7	XPNPEP1	69.92	1.45	1.06	2.60		1.00	0.73	1.79	0.00
715	P61086	UBE2K	22.41	1.11	1.55	1.85		1.00	1.40	1.66	0.00
716	Q96EV8	DTNBP1	39.49	0.88	1.01	1.32		1.00	1.14	1.50	0.00
717	Q15185	PTGES3	18.70	1.31	1.08	1.97		1.00	0.82	1.50	0.00
718	P61088	UBE2N	17.14	1.06	1.02	1.59		1.00	0.95	1.50	0.00
719	P10515	DLAT	69.00	0.68	0.90	0.97		1.00	1.33	1.44	0.00
720	P30084	ECHS1	31.39	0.75	0.86	1.06		1.00	1.14	1.41	0.00
721	O00299	CLIC1	26.92	1.01	0.93	1.42		1.00	0.92	1.40	0.00
722	Q16658	FSCN1	54.53	0.83	0.84	1.16		1.00	1.02	1.40	0.00
723	Q14376	GALE	38.28	0.72	0.75	1.00		1.00	1.04	1.39	0.00
724	Q96EP5	DAZAP1	43.38	0.94	2.80	1.30		1.00	2.96	1.37	0.00
725	P30626	SRI	21.68	0.76	0.81	1.02		1.00	1.06	1.33	0.00
726	P80217	IFI35	31.55	0.88	0.75	1.17		1.00	0.85	1.33	0.00
727	Q9GZZ1	NAA50	19.40	1.53	1.42	2.01		1.00	0.93	1.32	0.00
728	P62191	PSMC1	49.18	0.94	1.06	1.22		1.00	1.13	1.30	0.00
729	P31150	GDI1	50.58	1.02	0.95	1.32		1.00	0.93	1.29	0.00
730	O43242	PSMD3	60.98	1.30	1.52	1.66		1.00	1.17	1.28	0.00
731	P48556	PSMD8	39.61	1.10	1.41	1.37		1.00	1.28	1.24	0.00
732	P05198	EIF2S1	36.11	1.16	1.27	1.43		1.00	1.09	1.24	0.00

733	O00231	PSMD11	47.46	1.16	1.19	1.43	1.00	1.02	1.23	0.00
734	P52565	ARHGDIA	23.21	1.10	1.12	1.36	1.00	1.02	1.23	0.00
735	P43686	PSMC4	47.37	1.10	1.50	1.34	1.00	1.37	1.22	0.00
736	P36776	LONP1	106.49	0.89	0.91	1.09	1.00	1.02	1.22	0.00
737	Q96AC1	FERMT2	77.86	0.61	1.00	0.73	1.00	1.63	1.20	0.00
738	Q13838	DDX39B	48.99	1.20	1.19	1.43	1.00	1.00	1.20	0.00
739	P11172	UMPS	52.22	1.54	1.29	1.84	1.00	0.84	1.20	0.00
740	P21980	TGM2	77.33	0.39	0.43	0.46	1.00	1.11	1.19	0.00
741	P30837	ALDH1B1	57.21	0.54	0.63	0.64	1.00	1.17	1.18	0.00
742	Q13247	SRSF6	39.59	1.28	1.18	1.49	1.00	0.93	1.17	0.00
743	Q13177	PAK2	58.04	1.15	1.22	1.34	1.00	1.06	1.16	0.00
744	O75368	SH3BGRL	12.77	0.40	0.62	0.46	1.00	1.56	1.16	0.00
745	P62829	RPL23	14.87	1.21	1.19	1.38	1.00	0.99	1.14	0.00
746	Q16630	CPSF6	59.21	0.89	1.33	1.00	1.00	1.49	1.13	0.00
747	P51608	MECP2	52.44	0.67	0.74	0.75	1.00	1.10	1.11	0.00
748	O00232	PSMD12	52.90	1.22	1.27	1.35	1.00	1.05	1.11	0.00
749	P12931	SRC	59.83	0.62	0.60	0.69	1.00	0.96	1.11	0.00
750	P47813	EIF1AX	16.46	1.10	1.15	1.22	1.00	1.05	1.11	0.00
751	P33316	DUT	26.56	1.96	6.75	2.15	1.00	3.44	1.09	0.00
752	Q99460	PSMD1	105.84	1.25	1.56	1.36	1.00	1.24	1.09	0.00
753	Q96C01	FAM136A	15.64	1.16	1.01	1.25	1.00	0.87	1.08	0.00
754	P13693	TPT1	19.60	1.39	1.39	1.50	1.00	1.00	1.08	0.00
755	P08621	SNRNP70	51.56	1.36	1.26	1.47	1.00	0.93	1.08	0.00
756	Q9BZA7	PCDH11X	147.56	0.79	0.75	0.84	1.00	0.95	1.06	0.00
757	Q13885	TUBB2A	49.91	1.38	1.07	1.46	1.00	0.77	1.06	0.00
758	Q15257	PPP2R4	40.67	0.89	0.86	0.94	1.00	0.96	1.06	0.00
759	P32322	PYCR1	33.36	0.73	1.07	0.77	1.00	1.48	1.06	0.00
760	Q13200	PSMD2	100.20	1.23	1.29	1.29	1.00	1.05	1.05	0.00
761	O43837	IDH3B	42.18	1.06	0.95	1.10	1.00	0.90	1.04	0.00
762	P62333	PSMC6	44.17	1.24	1.31	1.28	1.00	1.05	1.03	0.00
763	O75436	VPS26A	38.17	0.91	1.18	0.93	1.00	1.30	1.02	0.00
764	O95989	NUDT3	19.47	0.83	0.86	0.85	1.00	1.04	1.02	0.00

765	O00487	PSMD14	34.58	1.28	1.39	1.30		1.00	1.08	1.01	0.00
766	Q7L9L4	MOB1B	25.09	0.99	0.87	1.01		1.00	0.87	1.01	0.00
767	Q9UNM6	PSMD13	42.95	1.20	1.21	1.20		1.00	1.01	1.00	0.00
768	P15927	RPA2	29.25	1.03	0.78	1.03		1.00	0.76	1.00	0.00
769	P62330	ARF6	20.08	0.81	0.89	0.81		1.00	1.09	1.00	0.00
770	Q969T9	WBP2	28.09	0.70	0.92	0.69		1.00	1.32	1.00	0.00
771	P62861	FAU	6.65	1.26	1.22	1.25		1.00	0.97	0.99	0.00
772	P35998	PSMC2	48.63	1.28	1.33	1.27		1.00	1.03	0.99	0.00
773	P62273	RPS29	6.68	1.12	1.26	1.10		1.00	1.13	0.98	0.00
774	P68400	CSNK2A1	45.14	1.29	1.25	1.26		1.00	0.97	0.98	0.00
775	Q9UQ80	PA2G4	43.79	1.34	1.25	1.30		1.00	0.94	0.97	0.00
776	P23246	SFPQ	76.15	1.25	0.87	1.21		1.00	0.69	0.97	0.00
777	Q14696	MESDC2	26.08	1.45	1.23	1.40		1.00	0.85	0.97	0.00
778	Q9Y376	CAB39	39.87	1.09	1.49	1.06		1.00	1.37	0.97	0.00
779	P23919	DTYMK	23.82	1.30	1.44	1.26		1.00	1.10	0.97	0.00
780	P30041	PRDX6	25.03	0.98	0.98	0.95		1.00	1.00	0.96	0.00
781	O14602	EIF1AY	16.44	1.35	1.34	1.30		1.00	0.99	0.96	0.00
782	P62195	PSMC5	45.63	1.22	1.35	1.15		1.00	1.10	0.94	0.00
783	Q96HE7	ERO1A	54.39	1.10	1.16	1.03		1.00	1.05	0.94	0.00
784	P08134	RHOC	22.01	1.40	1.12	1.32		1.00	0.80	0.94	0.00
785	P05091	ALDH2	56.38	0.70	0.75	0.65		1.00	1.08	0.94	0.00
786	P24752	ACAT1	45.20	0.83	0.87	0.77		1.00	1.05	0.92	0.00
787	P46063	RECQL	73.46	1.46	1.11	1.34		1.00	0.76	0.92	0.00
788	P17980	PSMC3	49.20	1.16	1.38	1.06		1.00	1.19	0.91	0.00
789	Q8NAF0	ZNF579	60.51	0.97	0.84	0.87		1.00	0.87	0.90	0.00
790	Q96FQ6	S100A16	11.80	0.79	1.76	0.70		1.00	2.24	0.89	0.00
791	P09960	LTA4H	69.28	1.03	0.98	0.91		1.00	0.95	0.88	0.00
792	Q9Y305	ACOT9	49.90	1.02	0.94	0.90		1.00	0.92	0.88	0.00
793	O75390	CS	51.71	1.16	1.35	1.00		1.00	1.16	0.86	0.00
794	Q9P0V9	SEPT10	52.59	1.12	1.15	0.96		1.00	1.02	0.86	0.00
795	P41091	EIF2S3	51.11	1.60	1.41	1.35		1.00	0.88	0.84	0.00
796	P67870	CSNK2B	24.94	1.73	1.33	1.45		1.00	0.77	0.84	0.00

797	Q9Y5Z4	HEBP2	22.88	1.16	1.08	0.97		1.00	0.93	0.83	0.00
798	P40261	NNMT	29.57	0.46	0.46	0.38		1.00	1.01	0.83	0.00
799	P13797	PLS3	70.81	0.92	0.90	0.75		1.00	0.99	0.82	0.00
800	Q9UHD8	KIAA0991	65.40	1.01	0.99	0.82		1.00	0.98	0.81	0.00
801	Q9NUQ9	FAM49B	36.75	1.34	1.11	1.08		1.00	0.82	0.81	0.00
802	P49419	ALDH7A1	58.49	0.89	0.68	0.71		1.00	0.76	0.79	0.00
803	Q9NY33	DPP3	82.59	0.99	0.88	0.78		1.00	0.89	0.79	0.00
804	Q15008	PSMD6	45.53	1.25	1.41	0.96		1.00	1.12	0.77	0.00
805	P31327	CPS1	164.94	0.93	0.98	0.71		1.00	1.06	0.76	0.00
806	Q86X55	CARM1	65.85	1.09	0.77	0.81		1.00	0.70	0.74	0.00
807	Q9BQA1	WDR77	36.72	1.61	1.41	1.18		1.00	0.88	0.74	0.00
808	P63279	UBE2I	18.01	1.13	1.14	0.82		1.00	1.00	0.73	0.00
809	P12081	HARS	57.41	1.20	1.40	0.85		1.00	1.17	0.71	0.00
810	O75223	GGCT	21.01	1.24	1.24	0.87		1.00	1.00	0.70	0.00
811	O15498	YKT6	22.42	1.56	1.51	1.08		1.00	0.97	0.69	0.00
812	P35579	MYH9	226.53	0.67	0.88	0.45		1.00	1.32	0.68	0.00
813	P32969	RPL9	21.86	1.59	1.06	1.05		1.00	0.67	0.66	0.00
814	P30622	CLIP1	162.24	1.91	1.66	1.26		1.00	0.87	0.66	0.00
815	P49591	SARS	58.78	1.00	1.16	0.65		1.00	1.16	0.65	0.00
816	Q16836	HADH	34.29	1.19	0.91	0.75		1.00	0.77	0.63	0.00
817	O14744	PRMT5	72.68	1.00	1.82	0.60		1.00	1.83	0.60	0.00
818	O75367	H2AFY	39.62	1.47	1.16	0.87		1.00	0.79	0.59	0.00
819	O75340	PDCD6	21.87	1.15	1.01	0.68		1.00	0.88	0.59	0.00
820	P54652	HSPA2	70.02	0.52	0.54	0.27		1.00	1.04	0.53	0.00
821	O75083	WDR1	66.19	0.91	0.82	0.46		1.00	0.91	0.50	0.00
822	Q06210	GFPT1	78.81	1.12	0.90	0.55		1.00	0.80	0.49	0.00
823	Q15046	KARS	68.05	1.23	1.20	0.54		1.00	0.98	0.44	0.00
824	P68402	PAFAH1B2	25.57	1.24	1.05	0.46		1.00	0.84	0.37	0.00
825	Q5JWF2	GNAS	111.02	3.21	0.87	0.82		1.00	0.27	0.26	0.00
826	O75323	GBAS	33.74	2.24	1.20	0.48		1.00	0.54	0.22	0.00
827	Q96FV2	SCRN2	46.60	0.83	100.00			1.00	121.07	0.00	0.00
828	P27824	CANX	67.57	0.87	100.00			1.00	115.21	0.00	0.00

829	Q9Y6I3	EPN1	60.29	1.00	100.00	1.00	100.00	0.00	0.00
830	O43390	HNRNPR	70.94	1.10	100.00	1.00	91.32	0.00	0.00
831	Q9Y224	C14orf166	28.07	1.12	100.00	1.00	89.29	0.00	0.00
832	Q92783	STAM	59.18	1.12	100.00	1.00	89.21	0.00	0.00
833	Q9HC35	EML4	108.92	1.14	100.00	1.00	87.57	0.00	0.00
834	O75821	EIF3G	35.61	1.23	100.00	1.00	81.17	0.00	0.00
835	Q9BZE9	ASPSCR1	60.18	1.35	100.00	1.00	74.07	0.00	0.00
836	Q96P70	IPO9	115.96	2.07	100.00	1.00	48.29	0.00	0.00
837	Q9BQ67	GRWD1	49.42	3.78	100.00	1.00	26.47	0.00	0.00
838	Q6PKG0	LARP1	123.51	1.61	14.38	1.00	8.93	0.00	0.00
839	Q9NR12	PDLIM7	49.84	0.42	1.96	1.00	4.73	0.00	0.00
840	Q9UBV8	PEF1	30.38	0.23	0.86	1.00	3.80	0.00	0.00
841	Q9NZL4	HSPBP1	39.47	1.52	4.19	1.00	2.75	0.00	0.00
842	Q16650	TBR1	74.05	0.61	1.65	1.00	2.70	0.00	0.00
843	Q7Z6Z7	HUWE1	481.89	1.05	2.84	1.00	2.69	0.00	0.00
844	P23921	RRM1	90.07	1.66	4.16	1.00	2.51	0.00	0.00
845	P33991	MCM4	96.56	1.36	3.01	1.00	2.22	0.00	0.00
846	Q9HAV7	GRPEL1	24.28	0.74	1.64	1.00	2.21	0.00	0.00
847	Q9BUF5	TUBB6	49.86	1.14	2.45	1.00	2.15	0.00	0.00
848	P50570	DNM2	98.06	1.35	2.85	1.00	2.11	0.00	0.00
849	Q9Y285	FARSA	57.56	1.18	2.29	1.00	1.94	0.00	0.00
850	P49915	GMPS	76.71	1.27	2.29	1.00	1.80	0.00	0.00
851	Q969G6	RFK	17.62	0.27	0.48	1.00	1.79	0.00	0.00
852	Q9P287	BCCIP	35.98	1.71	3.03	1.00	1.77	0.00	0.00
853	Q9NYU2	UGGT1	177.19	0.58	0.98	1.00	1.70	0.00	0.00
854	Q96GG9	DCUN1D1	30.12	0.82	1.38	1.00	1.69	0.00	0.00
855	Q02218	OGDH	115.93	0.76	1.27	1.00	1.67	0.00	0.00
856	Q9Y3E7	CHMP3	25.07	0.74	1.24	1.00	1.67	0.00	0.00
857	Q9UG63	ABCF2	71.29	1.04	1.68	1.00	1.61	0.00	0.00
858	P53041	PPP5C	56.88	0.91	1.42	1.00	1.57	0.00	0.00
859	P45974	USP5	95.79	0.69	1.08	1.00	1.57	0.00	0.00
860	P22314	UBA1	117.85	0.98	1.52	1.00	1.56	0.00	0.00

861	Q9UBQ5	EIF3K	25.06	1.33	2.06	1.00	1.55	0.00	0.00
862	Q7L576	CYFIP1	145.18	0.87	1.27	1.00	1.46	0.00	0.00
863	P52594	AGFG1	58.26	0.79	1.12	1.00	1.42	0.00	0.00
864	P12956	XRCC6	69.84	1.11	1.56	1.00	1.40	0.00	0.00
865	Q9UNF0	PACSIN2	55.74	0.86	1.21	1.00	1.40	0.00	0.00
866	Q9H4A4	RNPEP	72.60	0.77	1.07	1.00	1.40	0.00	0.00
867	Q15369	TCEB1	12.47	1.16	1.61	1.00	1.39	0.00	0.00
868	Q9BZZ5	API5	59.00	1.11	1.54	1.00	1.38	0.00	0.00
869	Q13310	PABPC4	70.78	1.40	1.92	1.00	1.38	0.00	0.00
870	Q9BR76	CORO1B	54.23	1.22	1.67	1.00	1.37	0.00	0.00
871	Q92598	HSPH1	96.86	1.39	1.90	1.00	1.37	0.00	0.00
872	Q14697	GANAB	106.87	1.17	1.58	1.00	1.36	0.00	0.00
873	P22087	FBL	33.78	0.90	1.18	1.00	1.31	0.00	0.00
874	Q9BV86	NTMT1	25.39	1.81	2.36	1.00	1.30	0.00	0.00
875	Q9UN86	G3BP2	54.12	0.87	1.13	1.00	1.30	0.00	0.00
876	P11586	MTHFD1	101.56	0.99	1.28	1.00	1.30	0.00	0.00
877	Q13492	PICALM	70.75	0.38	0.49	1.00	1.29	0.00	0.00
878	P26641	EEF1G	50.12	1.37	1.76	1.00	1.28	0.00	0.00
879	O60749	SNX2	58.47	1.63	2.07	1.00	1.27	0.00	0.00
880	O75937	DNAJC8	29.84	0.85	1.07	1.00	1.26	0.00	0.00
881	Q15691	MAPRE1	30.00	0.81	1.02	1.00	1.25	0.00	0.00
882	Q15121	PEA15	15.04	0.70	0.87	1.00	1.25	0.00	0.00
883	Q96A72	MAGOHB	17.28	1.99	2.48	1.00	1.25	0.00	0.00
884	O95359	TACC2	309.42	1.19	1.48	1.00	1.24	0.00	0.00
885	P62495	ETF1	49.03	1.18	1.46	1.00	1.24	0.00	0.00
886	Q9Y383	LUC7L2	46.51	0.98	1.20	1.00	1.23	0.00	0.00
887	P82933	MRPS9	45.83	0.91	1.12	1.00	1.23	0.00	0.00
888	Q9BS40	LXN	25.75	0.39	0.48	1.00	1.23	0.00	0.00
889	Q15382	RHEB	20.50	1.05	1.29	1.00	1.22	0.00	0.00
890	P61081	UBE2M	20.90	1.15	1.40	1.00	1.22	0.00	0.00
891	P51665	PSMD7	37.03	1.24	1.51	1.00	1.22	0.00	0.00
892	P26640	VARS	140.47	1.06	1.29	1.00	1.21	0.00	0.00

893	O75822	EIF3J	29.06	0.95	1.15	1.00	1.21	0.00	0.00
894	Q14764	MVP	99.33	0.58	0.70	1.00	1.21	0.00	0.00
895	Q9Y5X3	SNX5	46.82	0.76	0.90	1.00	1.19	0.00	0.00
896	P24534	EEF1B2	24.76	1.34	1.60	1.00	1.19	0.00	0.00
897	Q14974	KPNB1	97.17	1.15	1.35	1.00	1.18	0.00	0.00
898	O00429	DNM1L	81.88	1.09	1.28	1.00	1.17	0.00	0.00
899	P35080	PFN2	15.05	0.94	1.10	1.00	1.17	0.00	0.00
900	P53621	COPA	138.34	0.89	1.03	1.00	1.16	0.00	0.00
901	Q99798	ACO2	85.42	0.64	0.74	1.00	1.16	0.00	0.00
902	Q96C23	GALM	37.77	0.51	0.58	1.00	1.15	0.00	0.00
903	P14324	FDPS	48.28	1.08	1.25	1.00	1.15	0.00	0.00
904	P14550	AKR1A1	36.57	0.60	0.69	1.00	1.15	0.00	0.00
905	P40939	HADHA	83.00	1.06	1.22	1.00	1.14	0.00	0.00
906	Q07960	ARHGAP1	50.44	0.59	0.67	1.00	1.14	0.00	0.00
907	Q07157	TJP1	195.46	1.22	1.39	1.00	1.14	0.00	0.00
908	P12268	IMPDH2	55.80	1.21	1.37	1.00	1.14	0.00	0.00
909	Q6IBS0	TWF2	39.55	1.04	1.18	1.00	1.13	0.00	0.00
910	P63167	DYNLL1	10.37	1.00	1.13	1.00	1.13	0.00	0.00
911	O95433	AHSA1	38.27	1.18	1.33	1.00	1.13	0.00	0.00
912	P46087	NOP2	89.30	1.35	1.52	1.00	1.13	0.00	0.00
913	P40121	CAPG	38.50	0.77	0.87	1.00	1.12	0.00	0.00
914	O94925	GLS	73.46	1.04	1.17	1.00	1.12	0.00	0.00
915	P20073	ANXA7	52.74	0.73	0.82	1.00	1.12	0.00	0.00
916	Q9NQ88	TIGAR	30.06	0.97	1.09	1.00	1.12	0.00	0.00
917	Q9Y266	NUDC	38.24	1.52	1.68	1.00	1.11	0.00	0.00
918	P08559	PDHA1	43.30	0.84	0.92	1.00	1.10	0.00	0.00
919	P54920	NAPA	33.23	0.80	0.88	1.00	1.10	0.00	0.00
920	P18085	ARF4	20.51	0.95	1.04	1.00	1.10	0.00	0.00
921	Q6GMV2	SMYD5	47.34	1.25	1.37	1.00	1.10	0.00	0.00
922	Q13409	DYNC1I2	71.46	1.08	1.18	1.00	1.10	0.00	0.00
923	Q96CW1	AP2M1	49.65	0.82	0.89	1.00	1.09	0.00	0.00
924	P50552	VASP	39.83	0.66	0.72	1.00	1.09	0.00	0.00

925	Q9H3K6	BOLA2	10.12	2.15	2.35	1.00	1.09	0.00	0.00
926	P55209	NAP1L1	45.37	1.39	1.51	1.00	1.09	0.00	0.00
927	Q9BT78	COPS4	46.27	1.18	1.28	1.00	1.09	0.00	0.00
928	Q12931	TRAP1	80.11	1.21	1.32	1.00	1.08	0.00	0.00
929	Q02790	FKBP4	51.80	1.46	1.58	1.00	1.08	0.00	0.00
930	Q02750	MAP2K1	43.44	0.98	1.04	1.00	1.07	0.00	0.00
931	Q8ND56	LSM14A	50.53	1.11	1.19	1.00	1.07	0.00	0.00
932	Q15435	PPP1R7	41.56	0.92	0.98	1.00	1.06	0.00	0.00
933	Q9UHD1	CHORDC1	37.49	1.40	1.49	1.00	1.06	0.00	0.00
934	P49321	NASP	85.24	1.77	1.88	1.00	1.06	0.00	0.00
935	P22570	FDXR	53.84	0.83	0.87	1.00	1.05	0.00	0.00
936	Q96FW1	OTUB1	31.28	1.00	1.05	1.00	1.05	0.00	0.00
937	P17252	PRKCA	76.75	1.30	1.37	1.00	1.05	0.00	0.00
938	Q14204	DYNC1H1	532.40	1.09	1.14	1.00	1.05	0.00	0.00
939	Q9UBT2	UBA2	71.22	1.13	1.19	1.00	1.05	0.00	0.00
940	O60443	DFNA5	54.55	0.35	0.37	1.00	1.05	0.00	0.00
941	Q9Y5K6	CD2AP	71.45	0.67	0.70	1.00	1.05	0.00	0.00
942	O95819	MAP4K4	142.10	1.11	1.16	1.00	1.05	0.00	0.00
943	P53396	ACLY	120.84	1.08	1.12	1.00	1.04	0.00	0.00
944	P30740	SERPINB1	42.74	0.74	0.77	1.00	1.04	0.00	0.00
945	O43776	NARS	62.94	1.06	1.10	1.00	1.04	0.00	0.00
946	Q12792	TWF1	40.28	0.97	1.00	1.00	1.04	0.00	0.00
947	P25685	DNAJB1	38.04	1.09	1.13	1.00	1.03	0.00	0.00
948	Q9BVG4	PBDC1	26.06	1.71	1.77	1.00	1.03	0.00	0.00
949	O95793	STAU1	63.18	0.84	0.87	1.00	1.03	0.00	0.00
950	P30153	PPP2R1A	65.31	1.10	1.14	1.00	1.03	0.00	0.00
951	P58546	MTPN	12.89	1.01	1.04	1.00	1.03	0.00	0.00
952	O00469	PLOD2	84.69	1.25	1.28	1.00	1.02	0.00	0.00
953	Q13283	G3BP1	52.16	1.23	1.26	1.00	1.02	0.00	0.00
954	P26599	PTBP1	57.22	1.13	1.16	1.00	1.02	0.00	0.00
955	Q92499	DDX1	82.43	0.95	0.97	1.00	1.02	0.00	0.00
956	Q9NRN7	AASDHPPT	35.78	1.13	1.15	1.00	1.02	0.00	0.00

957	P48444	ARCN1	57.21	1.18	1.20	1.00	1.02	0.00	0.00
958	Q9Y570	PPME1	42.32	0.72	0.73	1.00	1.01	0.00	0.00
959	Q14651	PLS1	70.25	0.85	0.87	1.00	1.01	0.00	0.00
960	P36404	ARL2	20.88	0.99	1.00	1.00	1.01	0.00	0.00
961	P26639	TARS	83.43	1.09	1.10	1.00	1.01	0.00	0.00
962	Q9HAB8	PPCS	34.00	1.66	1.68	1.00	1.01	0.00	0.00
963	Q9NSD9	FARSB	66.11	1.15	1.16	1.00	1.01	0.00	0.00
964	P27695	APEX1	35.55	0.92	0.93	1.00	1.01	0.00	0.00
965	O76021	RSL1D1	54.97	1.61	1.62	1.00	1.01	0.00	0.00
966	Q16186	ADRM1	42.15	1.58	1.58	1.00	1.00	0.00	0.00
967	P11940	PABPC1	70.67	1.22	1.22	1.00	1.00	0.00	0.00
968	P26368	U2AF2	53.50	1.26	1.26	1.00	1.00	0.00	0.00
969	Q9NR45	NANS	40.31	0.88	0.88	1.00	1.00	0.00	0.00
970	P49327	FASN	273.42	0.87	0.86	1.00	0.99	0.00	0.00
971	Q96I99	SUCLG2	46.51	0.82	0.81	1.00	0.99	0.00	0.00
972	P48735	IDH2	50.91	0.94	0.94	1.00	0.99	0.00	0.00
973	O43765	SGTA	34.06	1.44	1.43	1.00	0.99	0.00	0.00
974	O60841	EIF5B	138.83	1.28	1.27	1.00	0.99	0.00	0.00
975	Q9Y316	MEMO1	33.73	0.99	0.98	1.00	0.99	0.00	0.00
976	P27708	CAD	242.98	1.66	1.64	1.00	0.99	0.00	0.00
977	P48506	GCLC	72.77	1.45	1.43	1.00	0.99	0.00	0.00
978	P35221	CTNNA1	100.07	0.47	0.46	1.00	0.99	0.00	0.00
979	O43237	DYNC1LI2	54.10	1.01	0.99	1.00	0.98	0.00	0.00
980	P56192	MARS	101.11	1.24	1.22	1.00	0.98	0.00	0.00
981	P49354	FNTA	44.41	1.46	1.44	1.00	0.98	0.00	0.00
982	P33176	KIF5B	109.68	1.13	1.11	1.00	0.98	0.00	0.00
983	P49589	CARS	85.47	0.96	0.94	1.00	0.98	0.00	0.00
984	O75828	CBR3	30.85	1.05	1.02	1.00	0.98	0.00	0.00
985	Q9Y6E0	STK24	49.31	1.15	1.12	1.00	0.97	0.00	0.00
986	Q12874	SF3A3	58.85	1.26	1.23	1.00	0.97	0.00	0.00
987	P08729	KRT7	51.39	0.98	0.95	1.00	0.97	0.00	0.00
988	Q96TA1	FAM129B	84.14	0.81	0.78	1.00	0.97	0.00	0.00

989	Q9HD15	SRA1	25.67	1.17	1.13			1.00	0.97	0.00	0.00
990	O15067	PFAS	144.73	1.48	1.43			1.00	0.97	0.00	0.00
991	Q16401	PSMD5	56.20	0.74	0.72			1.00	0.97	0.00	0.00
992	P53597	SUCLG1	36.25	0.77	0.75			1.00	0.97	0.00	0.00
993	Q9HB71	CACYBP	26.21	1.42	1.37			1.00	0.96	0.00	0.00
994	P46940	IQGAP1	189.25	0.88	0.85			1.00	0.96	0.00	0.00
995	Q01085	TIAL1	41.59	1.11	1.07			1.00	0.96	0.00	0.00
996	P24666	ACP1	18.04	1.25	1.20			1.00	0.96	0.00	0.00
997	Q9UHY7	ENOPH1	28.93	1.23	1.18			1.00	0.96	0.00	0.00
998	O15372	EIF3H	39.93	1.17	1.12			1.00	0.96	0.00	0.00
999	Q96QK1	VPS35	91.71	0.98	0.93			1.00	0.96	0.00	0.00
1000	Q08211	DHX9	140.96	1.38	1.32			1.00	0.96	0.00	0.00
1001	P55084	HADHB	51.29	0.75	0.71			1.00	0.95	0.00	0.00
1002	Q9H0E2	TOLLIP	30.28	1.04	0.99			1.00	0.95	0.00	0.00
1003	Q9Y696	CLIC4	28.77	0.76	0.72			1.00	0.95	0.00	0.00
1004	Q712K3	UBE2R2	27.17	1.70	1.62			1.00	0.95	0.00	0.00
1005	P34932	HSPA4	94.33	1.26	1.20			1.00	0.95	0.00	0.00
1006	Q13596	SNX1	59.07	0.77	0.73			1.00	0.95	0.00	0.00
1007	Q99873	PRMT1	41.52	1.21	1.15			1.00	0.95	0.00	0.00
1008	P08243	ASNS	64.37	2.23	2.10			1.00	0.94	0.00	0.00
1009	Q9HC38	GLOD4	34.79	1.02	0.96			1.00	0.94	0.00	0.00
1010	Q9Y490	TLN1	269.76	0.88	0.82			1.00	0.94	0.00	0.00
1011	P78527	PRKDC	469.08	1.05	0.99			1.00	0.94	0.00	0.00
1012	Q6YN16	HSDL2	45.39	1.22	1.13			1.00	0.93	0.00	0.00
1013	Q99829	CPNE1	59.06	0.98	0.91			1.00	0.93	0.00	0.00
1014	Q15370	TCEB2	13.13	1.32	1.22			1.00	0.92	0.00	0.00
1015	P19367	HK1	102.49	1.28	1.18			1.00	0.92	0.00	0.00
1016	O43252	PAPSS1	70.83	1.33	1.22			1.00	0.92	0.00	0.00
1017	P12429	ANXA3	36.37	0.60	0.55			1.00	0.92	0.00	0.00
1018	O00159	MYO1C	121.68	0.78	0.71			1.00	0.92	0.00	0.00
1019	P35606	COPB2	102.49	1.38	1.27			1.00	0.92	0.00	0.00
1020	P51991	HNRNPA3	39.60	1.09	1.00			1.00	0.92	0.00	0.00

1021	Q6FI81	CIAPIN1	33.58	2.72	2.48	1.00	0.91	0.00	0.00
1022	P53618	COPB1	107.14	1.33	1.21	1.00	0.91	0.00	0.00
1023	O60884	DNAJA2	45.75	1.28	1.17	1.00	0.91	0.00	0.00
1024	P33993	MCM7	81.31	1.31	1.19	1.00	0.91	0.00	0.00
1025	Q15436	SEC23A	86.16	0.94	0.85	1.00	0.91	0.00	0.00
1026	Q99426	TBCB	27.33	0.60	0.54	1.00	0.90	0.00	0.00
1027	Q9Y2Q3	GSTK1	25.50	0.72	0.65	1.00	0.90	0.00	0.00
1028	Q13561	DCTN2	44.23	1.14	1.03	1.00	0.90	0.00	0.00
1029	O00170	AIP	37.64	1.01	0.91	1.00	0.90	0.00	0.00
1030	O95782	AP2A1	107.54	0.95	0.85	1.00	0.90	0.00	0.00
1031	Q9UHX1	PUF60	59.88	1.04	0.93	1.00	0.90	0.00	0.00
1032	P49736	MCM2	101.90	1.58	1.40	1.00	0.89	0.00	0.00
1033	P30085	CMPK1	22.22	0.77	0.68	1.00	0.89	0.00	0.00
1034	Q9UBE0	SAE1	38.45	1.27	1.12	1.00	0.88	0.00	0.00
1035	Q8WXX5	DNAJC9	29.91	1.63	1.44	1.00	0.88	0.00	0.00
1036	Q01082	SPTBN1	274.61	1.28	1.13	1.00	0.88	0.00	0.00
1037	Q06124	PTPN11	68.44	1.41	1.24	1.00	0.88	0.00	0.00
1038	P63010	AP2B1	104.55	0.87	0.76	1.00	0.88	0.00	0.00
1039	P30419	NMT1	56.81	1.24	1.09	1.00	0.88	0.00	0.00
1040	P26196	DDX6	54.42	0.89	0.78	1.00	0.87	0.00	0.00
1041	P49588	AARS	106.81	1.07	0.93	1.00	0.87	0.00	0.00
1042	P55060	CSE1L	110.42	1.40	1.20	1.00	0.86	0.00	0.00
1043	P54886	ALDH18A1	87.30	0.88	0.76	1.00	0.86	0.00	0.00
1044	Q14533	KRT81	54.93	1.08	0.93	1.00	0.86	0.00	0.00
1045	P50502	ST13	41.33	0.99	0.85	1.00	0.86	0.00	0.00
1046	Q15366	PCBP2	38.58	1.16	0.99	1.00	0.85	0.00	0.00
1047	P40123	CAP2	52.82	0.69	0.58	1.00	0.85	0.00	0.00
1048	Q92905	COPS5	37.58	1.14	0.97	1.00	0.85	0.00	0.00
1049	P26885	FKBP2	15.65	1.01	0.85	1.00	0.84	0.00	0.00
1050	O95340	PAPSS2	69.50	0.78	0.65	1.00	0.84	0.00	0.00
1051	Q6NVY1	HIBCH	43.48	0.77	0.65	1.00	0.84	0.00	0.00
1052	Q8WX93	PALLD	150.56	0.79	0.66	1.00	0.84	0.00	0.00

1053	Q96HC4	PDLIM5	63.94	0.73	0.62	1.00	0.84	0.00	0.00
1054	Q9UL46	PSME2	27.40	0.98	0.82	1.00	0.84	0.00	0.00
1055	Q9NZU5	LMCD1	40.83	0.59	0.50	1.00	0.84	0.00	0.00
1056	P13489	RNH1	49.97	1.17	0.97	1.00	0.83	0.00	0.00
1057	Q96E11	MRRF	29.28	2.17	1.80	1.00	0.83	0.00	0.00
1058	Q9NTK5	OLA1	44.74	1.32	1.09	1.00	0.83	0.00	0.00
1059	O43396	TXNL1	32.25	1.12	0.92	1.00	0.83	0.00	0.00
1060	Q12765	SCRN1	46.38	0.85	0.70	1.00	0.82	0.00	0.00
1061	Q9Y678	COPG1	97.72	0.92	0.75	1.00	0.82	0.00	0.00
1062	Q9UGI8	TES	48.00	1.01	0.83	1.00	0.82	0.00	0.00
1063	P14868	DARS	57.14	1.00	0.82	1.00	0.82	0.00	0.00
1064	Q15393	SF3B3	135.58	1.26	1.02	1.00	0.81	0.00	0.00
1065	Q99733	NAP1L4	42.82	1.40	1.14	1.00	0.81	0.00	0.00
1066	Q8NBJ7	SUMF2	33.84	1.21	0.97	1.00	0.81	0.00	0.00
1067	Q5JRX3	PITRM1	117.41	1.33	1.07	1.00	0.80	0.00	0.00
1068	P05455	SSB	46.84	1.07	0.86	1.00	0.80	0.00	0.00
1069	P06396	GSN	85.70	0.51	0.40	1.00	0.79	0.00	0.00
1070	Q92841	DDX17	80.27	0.98	0.77	1.00	0.78	0.00	0.00
1071	Q9BWJ5	SF3B5	10.14	1.88	1.47	1.00	0.78	0.00	0.00
1072	Q9ULV4	CORO1C	53.25	1.52	1.18	1.00	0.78	0.00	0.00
1073	Q14566	MCM6	92.89	2.20	1.71	1.00	0.78	0.00	0.00
1074	Q13620	CUL4B	103.98	1.45	1.12	1.00	0.77	0.00	0.00
1075	Q9Y2A7	NCKAP1	128.79	1.09	0.84	1.00	0.77	0.00	0.00
1076	P49411	TUFM	49.54	0.97	0.75	1.00	0.77	0.00	0.00
1077	O75131	CPNE3	60.13	0.94	0.72	1.00	0.77	0.00	0.00
1078	P15374	UCHL3	26.18	1.39	1.06	1.00	0.76	0.00	0.00
1079	P15428	HPGD	28.98	1.35	1.03	1.00	0.76	0.00	0.00
1080	P54578	USP14	56.07	1.14	0.86	1.00	0.76	0.00	0.00
1081	O43681	ASNA1	38.79	1.10	0.83	1.00	0.75	0.00	0.00
1082	Q9H4M9	EHD1	60.63	1.12	0.84	1.00	0.75	0.00	0.00
1083	P19623	SRM	33.82	1.73	1.30	1.00	0.75	0.00	0.00
1084	Q9UMY4	SNX12	19.73	0.88	0.66	1.00	0.75	0.00	0.00

1085	Q13435	SF3B2	100.23	1.43	1.07	1.00	0.75	0.00	0.00
1086	O95232	LUC7L3	51.47	1.79	1.33	1.00	0.75	0.00	0.00
1087	O76003	GLRX3	37.43	1.58	1.18	1.00	0.75	0.00	0.00
1088	Q13557	CAMK2D	56.37	0.90	0.67	1.00	0.74	0.00	0.00
1089	P51452	DUSP3	20.48	1.10	0.79	1.00	0.72	0.00	0.00
1090	Q9Y5L0	TNPO3	104.20	2.00	1.44	1.00	0.72	0.00	0.00
1091	Q13618	CUL3	88.93	1.69	1.21	1.00	0.72	0.00	0.00
1092	Q15459	SF3A1	88.89	0.89	0.63	1.00	0.71	0.00	0.00
1093	P08133	ANXA6	75.87	0.72	0.51	1.00	0.71	0.00	0.00
1094	O95757	HSPA4L	94.51	2.87	2.03	1.00	0.71	0.00	0.00
1095	O14562	UBFD1	33.38	1.47	1.04	1.00	0.70	0.00	0.00
1096	Q9H074	PAIP1	53.52	1.18	0.83	1.00	0.70	0.00	0.00
1097	Q99848	EBNA1BP2	34.85	2.26	1.58	1.00	0.70	0.00	0.00
1098	P51570	GALK1	42.27	1.03	0.72	1.00	0.70	0.00	0.00
1099	P11177	PDHB	39.23	0.95	0.65	1.00	0.69	0.00	0.00
1100	P55010	EIF5	49.22	1.61	1.11	1.00	0.69	0.00	0.00
1101	Q9P258	RCC2	56.08	2.49	1.71	1.00	0.69	0.00	0.00
1102	P50995	ANXA11	54.39	0.85	0.58	1.00	0.68	0.00	0.00
1103	P33992	MCM5	82.28	2.32	1.59	1.00	0.68	0.00	0.00
1104	Q8NE71	ABCF1	95.93	1.43	0.97	1.00	0.68	0.00	0.00
1105	P30038	ALDH4A1	61.72	0.64	0.43	1.00	0.68	0.00	0.00
1106	Q99447	PCYT2	43.84	0.79	0.53	1.00	0.67	0.00	0.00
1107	Q5SSJ5	HP1BP3	61.21	0.72	0.48	1.00	0.67	0.00	0.00
1108	Q16822	PCK2	70.73	0.96	0.64	1.00	0.67	0.00	0.00
1109	Q9BY44	EIF2A	64.99	1.88	1.24	1.00	0.66	0.00	0.00
1110	Q13098	GPS1	55.54	1.48	0.94	1.00	0.64	0.00	0.00
1111	Q8IWE2	FAM114A1	60.74	1.07	0.68	1.00	0.64	0.00	0.00
1112	P35580	MYH10	229.00	1.00	0.63	1.00	0.63	0.00	0.00
1113	P25205	MCM3	90.98	1.55	0.97	1.00	0.62	0.00	0.00
1114	Q8TAT6	NPLOC4	68.12	1.65	1.01	1.00	0.61	0.00	0.00
1115	Q9P2R7	SUCLA2	50.32	1.06	0.65	1.00	0.61	0.00	0.00
1116	Q13185	CBX3	20.81	1.21	0.74	1.00	0.61	0.00	0.00

1117	P06493	CDK1	34.10	1.86	1.12	1.00	0.60	0.00	0.00
1118	P62256	UBE2H	20.66	2.56	1.52	1.00	0.59	0.00	0.00
1119	Q9NP79	VTA1	33.88	1.38	0.82	1.00	0.59	0.00	0.00
1120	Q9NRV9	HEBP1	21.10	0.97	0.56	1.00	0.58	0.00	0.00
1121	P61077	UBE2D3	16.69	1.73	1.00	1.00	0.58	0.00	0.00
1122	O75688	PPM1B	52.64	0.84	0.48	1.00	0.57	0.00	0.00
1123	Q9BXT2	CACNG6	28.13	0.79	0.45	1.00	0.57	0.00	0.00
1124	P50454	SERPINH1	46.44	1.28	0.70	1.00	0.55	0.00	0.00
1125	Q9BVJ7	DUSP23	16.59	1.07	0.57	1.00	0.54	0.00	0.00
1126	O14964	HGS	86.19	2.10	1.11	1.00	0.53	0.00	0.00
1127	Q9BS26	ERP44	46.97	1.35	0.71	1.00	0.53	0.00	0.00
1128	P41223	BUD31	17.00	1.41	0.73	1.00	0.52	0.00	0.00
1129	O75643	SNRNP200	244.51	1.80	0.92	1.00	0.51	0.00	0.00
1130	Q9H832	UBE2Z	38.21	1.68	0.84	1.00	0.50	0.00	0.00
1131	Q96RS6	NUDCD1	66.76	1.47	0.73	1.00	0.50	0.00	0.00
1132	Q07866	KLC1	65.31	1.21	0.57	1.00	0.47	0.00	0.00
1133	Q9UPQ0	LIMCH1	121.87	0.51	0.24	1.00	0.47	0.00	0.00
1134	Q9UKD2	MRTO4	27.56	3.24	1.43	1.00	0.44	0.00	0.00
1135	Q9H4G0	EPB41L1	98.50	1.17	0.47	1.00	0.40	0.00	0.00
1136	Q9Y262	EIF3L	66.73	1.18	0.47	1.00	0.40	0.00	0.00
1137	Q96DG6	CMBL	28.05	0.95	0.38	1.00	0.40	0.00	0.00
1138	Q92597	NDRG1	42.84	1.63	0.64	1.00	0.39	0.00	0.00
1139	Q8IYB3	SRRM1	102.33	1.97	0.74	1.00	0.38	0.00	0.00
1140	Q9H3P7	ACBD3	60.59	1.41	0.53	1.00	0.37	0.00	0.00
1141	O43488	AKR7A2	39.59	1.00	0.37	1.00	0.37	0.00	0.00
1142	Q9NZN4	EHD2	61.16	0.69	0.25	1.00	0.36	0.00	0.00
1143	P51553	IDH3G	42.79	1.94	0.55	1.00	0.28	0.00	0.00
1144	Q9Y547	HSPB11	16.30	19.44	3.39	1.00	0.17	0.00	0.00
1145	P19784	CSNK2A2	41.21	1.53	0.18	1.00	0.12	0.00	0.00
1146	P46108	CRK	33.83	1.06	0.01	1.00	0.01	0.00	0.00
1147	Q6P2Q9	PRPF8	273.60	25.27	0.01	1.00	0.00	0.00	0.00

Table S4. Sequence of primers for qPCR, vector constrastion and Oligonucleotides.

qPCR-Primers	Sense (F)	Antisense (R)
DR5	5’-CGCTGCACCAGGTGTGATT-3’	5’-GTGCCTTCTTCGCACTGACA-3’
Actin	5’-GAAGAGCTACGAGCTGCCTGA-3’	5’-CAGACAGCACTGTGTTGGCG-3’

Oligonucleotides	Sequence
sh-CHOP#1	CCGGGCCAATGATGTGACCCTCAATCTCGAGATTGAGGGTCACATCATTGGCTTTTT
sh-CHOP#2	CCGGCCTGGAAATGAAGAGGAAGAACTCGAGTTCTTCCTCTTCATTTCCAGGTTTTT
sh-DR5#1	CCGGGCAGTCTCATTTGCACCCATACTCGAGTATGGGTGCAAATGAGACTGCTTTTT
sh-DR5#2	CCGGCCACAAAGAATCAGGTACAAACTCGAGTTTGTACCTGATTCTTTGTGGTTTTT
si-NQO2#1	GGCTGACCTAGTGATATTT
si-NQO2#2	CCGGTTTGCTCCAGGGTAA

Recombinant DNA	Vector	Primers
DR5-P(-552/+3)	pGL3	F: 5’-ATTTCTCTATCGATA GGTACC AGAGAACAGAAGGGGCAGG-3’<br>R: 5’-CAGTACCGGAATGCC AAGCTT GGCGGTAGGGAACGCTCTTATAG-3’
DR5-P(mtCHOP)	pGL3	F: 5’-TTGGACGCGCTTGCGGAGGACATAGTTGACGAGA-3’<br>R: 5’-TATGTCCTCCGCAAGCGCGTCCAAGTGGGGAGGG-3’
KO-NQO2	CRISPR/Cas9	F: 5’-GGCCCTCGGCTCAAGGTTCAGTTTTAGAGCTAGAAATAGCAAGTT-3’<br>R: 5’-TGAACCTTGAGCCGAGGGCCCGGTGTTTCGTCCTTTCCACAAGAT-3’
NQO2-WT	pLenti-CMV	F: 5’-CGGAATTCATGGCAGGTAAGAAAGTACTCATTGTC-3’<br>R: 5’-GCTCTAGATTACTTATCGTCGTCATCCTTGTAATCTTGCCCGAAGTGCCAGTGGGCTGT-3’
NQO2-WT	pGEX-4T-1	F: 5’-CCGCGTGGATCCCCGGAATTCATGGCAGGTAAGAAAGTA-3’<br>R: 5’-CTCGAGTCGACCCGGGAATTCTTACTTATCGTCGTCATC-3’
NQO2-F126A	pLenti-CMV, pGEX-4T-1	F: 5’-GGGCTTTGCCGCTGACATCCCAGGATTCTACGATTC-3’<br>R: 5’-GGGATGTCAGCGGCAAAGCCCTGGCACAGCACCCTA-3’
NQO2-I128A	pLenti-CMV, pGEX-4T-1	F: 5’-TGCCTTTGACGCCCCAGGATTCTACGATTCCGGTTT-3’<br>R: 5’-AATCCTGGGGCGTCAAAGGCAAAGCCCTGGCACAGC-3’
NQO2-N161A	pLenti-CMV, pGEX-4T-1	F: 5’-ACAGGAGTCGCTGGAGATTCTCGATACTTCCTGTGG-3’<br>R: 5’-AGAATCTCCAGCGACTCCTGTCTTCGTGTACATCTC-3’
NQO2-F178A	pLenti-CMV, pGEX-4T-1	F: 5’-GCACATTACACGCCTGTGGATTTAAAGTCCTTGCCC-3’<br>R: 5’-ATCCACAGGCGTGTAATGTGCCATGCTGGAGTGGCC-3’
